# Trends and projections of age-appropriate vaccination coverage in 41 low- and middle- income countries in Asia and Sub-Saharan Africa, 2000–2030

**DOI:** 10.3389/fpubh.2024.1371258

**Published:** 2024-05-09

**Authors:** Md Rashedul Islam, Md Mizanur Rahman, Md Shafiur Rahman, Sarah Krull Abe, Manas K. Akmatov, Masahiro Hashizume

**Affiliations:** ^1^Hitotsubashi Institute for Advanced Study, Hitotsubashi University, Tokyo, Japan; ^2^Department of Global Health Policy, Graduate School of Medicine, The University of Tokyo, Tokyo, Japan; ^3^Research Centre for Child Mental Development, Hamamatsu University School of Medicine, Hamamatsu, Japan; ^4^United Graduate School of Child Development, Osaka University, Kanazawa University, Hamamatsu University School of Medicine, Chiba University and University of Fukui, Osaka, Japan; ^5^Division of Prevention, Institute for Cancer Control, National Cancer Center Japan, Tokyo, Japan; ^6^Department of Epidemiology and Health Care Atlas, Central Research Institute of Ambulatory Health Care, Berlin, Germany

**Keywords:** age-appropriate childhood vaccination, coverage, VPDs, trends, projection, Bayesian model

## Abstract

**Introduction:**

Routine immunization programs have focused on increasing vaccination coverage, which is equally important for decreasing vaccine-preventable diseases (VPDs), particularly in low- and lower-middle-income countries (LMICs). We estimated the trends and projections of age-appropriate vaccination coverage at the regional and national levels, as well as place of residence and wealth index in LMICs.

**Methods:**

In total, 174 nationally representative household surveys from 2000 to 2020 from 41 LMICs were included in this study. Bayesian hierarchical regression models were used to estimate trends and projections of age-appropriate vaccination.

**Results:**

The trend in coverage of age-appropriate Bacillus Calmette-Guérin (BCG), third dose of diphtheria, tetanus, and pertussis (DTP3), third dose of polio (polio3), and measles-containing vaccine (MCV) increased rapidly from 2000 to 2020 in LMICs. Findings indicate substantial increases at the regional and national levels, and by area of residence and socioeconomic status between 2000 and 2030. The largest rise was observed in East Africa, followed by South and Southeast Asia. However, out of the 41 countries, only 10 countries are estimated to achieve 90% coverage of the BCG vaccine by 2030, five of DTP3, three of polio3, and none of MCV. Additionally, by 2030, wider pro-urban and -rich inequalities are expected in several African countries.

**Conclusion:**

Significant progress in age-appropriate vaccination coverage has been made in LMICs from 2000 to 2020. Despite this, projections show many countries will not meet the 2030 coverage goals, with persistent urban–rural and socioeconomic disparities. Therefore, LMICs must prioritize underperforming areas and reduce inequalities through stronger health systems and increased community engagement to ensure high coverage and equitable vaccine access.

## 1 Introduction

Childhood immunization is considered one of the most proven, successful, and cost-effective child survival public health interventions (1). Globally, significant progress has been made in vaccination coverage since 1974 after the initiation of the Expanded Program on Immunization (EPI), which dramatically reduced 2–3 million annual avertable child deaths from vaccine-preventable diseases (VPDs) (2). Yet, according to the World Health Organization (WHO), an estimated 1.5 million children die every year from VPDs, mostly in low- and lower-middle income countries (LMICs) of Africa and Asia (3), indicating that compliance with the recommended vaccination schedule is a challenge for fragile healthcare systems. Hence, the Global Vaccine Action Plan (GVAP) 2011–20 set a target to reach 90% coverage of EPI vaccines at the national level by 2020, following which the Immunization Agenda 2030 (IA2030) also aims to leave no one behind routine immunization in each country by 2030 (4). Despite these commitments, various persistent and new challenges, such as lack of infrastructure and/or required human resources, high dropouts, shortage of supplies, vaccine stock out, cold chain breakages, lack of immunization culture, and even the emergence of coronavirus disease (COVID-19) (5–7), result in delayed, incomplete, and low vaccination coverage, especially in LMICs. Thus, to evaluate the progress towards achieving these targets, it is important to clearly understand the coverage gaps both before and during the onset of COVID-19.

Age-appropriate vaccines have, thus, been identified as an essential health intervention for child protection against mortality and morbidity, which has been closely looked at in many countries in recent years (8–12). The design of its schedule is based on when the immune system can safely respond to the vaccine and age-related diseases that can impact child morbidity and mortality, through which it can reduce the risk of infection of VPDs among infants and children in early life (13). Thus, incorrectly timed vaccinations may partially explain the persistence and reemergence of VPDs (11, 14). However, some reviews reported lack of concordance in the definition of age-appropriate vaccination (10, 11). Previous studies suggest that only a few children receive timely vaccines in their recommended age, even with high vaccination coverage (11, 12, 15–18). In addition, a secondary analysis of Demographic and Health Surveys (DHS) and Multiple Indicator Cluster Surveys (MICS) also indicated maternal education, place of residence and socioeconomic status as most common factors associated with lower vaccination coverage (12, 19). However, studies revealing coverage trends combined with projections in coverage among different LMICs are scarce.

To date, no study has assessed the progress towards age-appropriate vaccinations in LMICs at regional and national levels, as well as by place of residence and wealth index. Since the emergence of COVID-19, national immunization programs in most LMICs have been affected, resulting in an additional burden on the health systems, halting their progress, and putting the provision of age-appropriate vaccination coverage at risk. Moreover, owing to economic constraints, health systems in LMICs are expected to experience a reduction in development assistance for health (DAH) and health worker density to some extent. Therefore, the objective of this study is to estimate recent trends in the coverage of age-appropriate vaccinations and to derive projections of these indicators up to 2030 for 41 LMICs at regional and national levels, as well as by place of residence and wealth index. Second, we evaluated the impact of decreasing DAH and skilled health workforce (SHW) due to COVID-19 on age-appropriate vaccination coverage.

## 2 Methods

### 2.1 Data sources

This study includes LMICs from South Asia, Southeast Asia, and Sub-Saharan Africa regions according to the World Bank’s 2020 list of countries by income (20). To be included, at least two nationally representative household surveys (DHS and MICS) had to be conducted since 2000 and including date of birth of children. Vaccination data were extracted from 174 nationally representative household surveys conducted from 2000 to 2020. In addition, the countries were grouped into several regions based on the definition of the United Nations Statistical Division (21). The list of countries and surveys including percentage of vaccination card at national as well as age group are presented in the Supplementary Table S1.

### 2.2 Outcome variables

The outcome variables of this study comprised a single dose of Bacillus Calmette-Guérin (BCG); third dose of diphtheria, tetanus, and pertussis (DTP3); third dose of polio (polio3); and first dose of a measles-containing vaccine (MCV). WHO recommendations were used to assess age-appropriate vaccination in accordance with the respective national immunization schedules of each country (22). Timely or age-appropriate vaccination was defined as receiving a vaccine within four weeks of the recommended age specified in the national immunization schedule (Supplementary Table S2). The timing was determined by comparing the observed age at receipt to the recommended age for each vaccine dose. Age at vaccination was calculated based on documented birthdate and dates at vaccination. In case of series of DPT and polio vaccines, if the minimum interval was less than 28 days, then it was not considered as age-appropriate vaccination. A delay in vaccination was defined as those receiving the vaccine 4 weeks after the WHO recommended age in each country’s immunization schedule.

### 2.3 Independent variables

Based on previous literature, the country- and year-specific socio-demographic index (SDI), government spending on health (GSH), DAH, and skilled health workforce (SHW) were used to estimate the trends and projections of age-appropriate vaccination (23, 24). We extracted data on SDI, GSH, and DAH from the IHME database on the Global Health Data Exchange website.^1^ The density of SHW was defined as the number of doctors, nurses, and midwives per 1,000 people. Country-specific data on SHW for the period 2000–2017 (as of July 1, 2019) were extracted from the WHO Global Health Observatory Data Repository.^2^

### 2.4 Statistical analysis

Using the Kaplan–Meier method, coverage of age-appropriate vaccinations was estimated from micro-data. Thereafter, a Bayesian hierarchical linear regression model was applied to estimate the trends among 41 countries and develop projections of each outcome variable at the regional, national, area of residence, and wealth quintile levels. The proportion (coverage) of all outcome variables was transformed into logit scales before modeling. We fitted the models using the Bayesian approach, sampling from the posterior distribution of the parameters using Gibbs Monte Carlo, a Markov chain Monte Carlo (MCMC) method, as implemented in the algorithm in the JAGS open-source software (version 4.2). Detailed information on the model parameters and sensitivity analysis is presented in the Supplementary Appendix (e-method 1–2). To estimate the impact of COVID-19 on the coverage of age-appropriate vaccines, we projected three scenarios: (1) coverage under the current trends, (2) coverage with DAH reduction, and (3) coverage with SHW reduction. The models for both scenarios included the same covariate matrix that was used for the national-level model in the current trend scenario. In the DAH reduction scenario, we assumed that the level of DAH in 2020 would reduce by 10% from 2019, and that this level will remain constant until 2030. Similarly, in the SHW reduction scenario, we considered that the density of SHW in 2020 was reduced by 10% from the value in 2019 but will remain the same until 2030.

## 3 Results

### 3.1 Study characteristics

A total of 110 DHS and 64 MICS household surveys conducted during 2000–2020 in 41 LMICs were included in this study. The country-specific survey data points with survey types are presented in the Supplementary Figure S1 and Supplementary Table S1. The majority of the included countries (62%) had 4 or more data points for this analysis.

### 3.2 Regional level estimate of age-appropriate vaccination coverage

**Figure 1:**
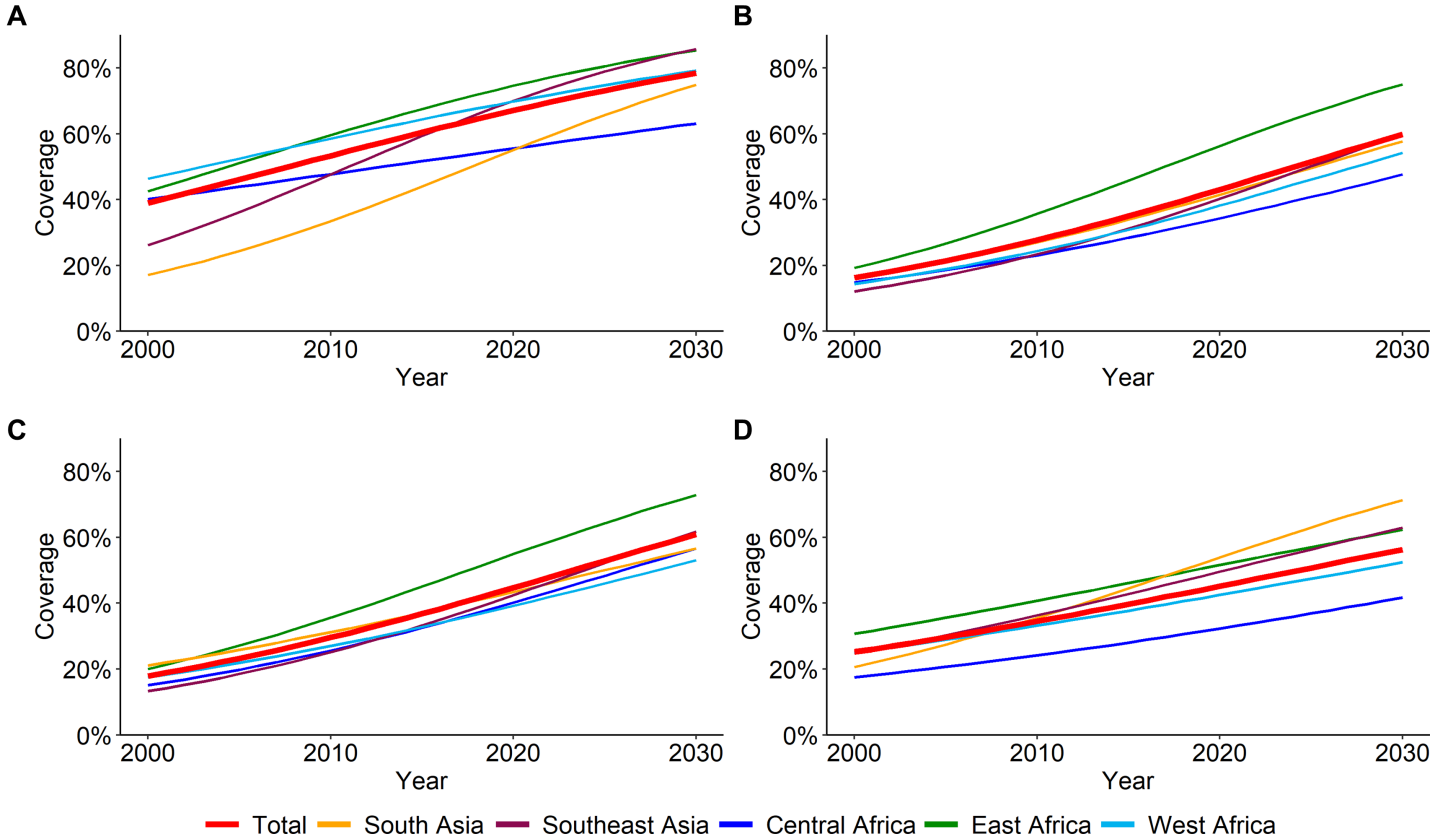
Overall and regional coverage of age-appropriate vaccines from 2000 to 2030. **(A)** BCG **(B)** DPT3 **(C)** Polio3 **(D)** MCV.

The predicted coverage with 95% credible interval (CrI), and the annual and required rates of change for age-appropriate BCG, DTP3, polio3, and MCV vaccines during 2000–2030 are presented in Figure 1 and Supplementary Table S3. Overall, the coverage of age-appropriate BCG vaccination in all 41 LMICs has increased from 39.0% in 2000 to 67.1% in 2020 and is predicted to reach 78.4% by 2030. The largest increase in BCG vaccine between 2000 and 2030 was observed in Southeast Asia (from 59.5% in 2000 and 26.2% in 2020 to 85.7% in 2030), followed by South Asia (from 57.8, 17.1 to 74.9%), and East Africa (from 42.8 to 42.5 to 85.3%). By 2030, the highest coverage of the DTP3 vaccine is predicted to be 75.0% in East Africa, 59.9% in Southeast Asia, and 57.8% in South Asia (Figure 1B; Supplementary Table S3). If the current trend continues, the coverage of polio3 vaccines in 41 Asian and African countries is expected to be 44.7 and 60.9% in 2030, respectively (Figure 1C; Supplementary Table S3). Region-wise, the highest predicted coverage of polio3 vaccine in 2030 would be in East Africa (72.8%) and Southeast Africa (61.8%). In the case of MCV, the largest coverage is projected to be in Southeast Asia (71.3%), followed by South Asia (62.9%), and East Africa (62.3%) in 2030. The overall coverage is predicted to increase from 45.2 to 56.3% between 2000 and 2030 (Figure 1D; Supplementary Table S3).

### 3.3 National level estimate of vaccination coverage

The coverage of age-appropriate BCG and DTP3 vaccines at the national level from 2000 to 2030 is presented in Table 1. In 2020, only five countries, Cambodia, Sao Tome and Principe, Burundi, Rwanda, and Zimbabwe, have reached over 90% coverage of BCG vaccination, but no country has achieved 90% DTP3 coverage. If the current trends continue, 11 out of 41 LMICs are predicted to achieve over 90% BCG coverage by 2030, whereas only five countries will achieve over 90% coverage of DTP3 vaccination. The highest annual rate of change for the BCG vaccine was observed in the Democratic Republic of Congo (11.8%) and Zimbabwe (17.2%) during 2000–2020, whereas slow progress was made in Benin (0.3%) and Cote d’Ivoire (0.3%) (Supplementary Table S4).

**Table 1:** National level coverage of age-appropriate BCG and DTP3 vaccine in 41 LMICs in Asia and Sub-Saharan Africa, 2000–2030.

Country	Predicted coverage (95% credible intervals)					
	BCG			DTP3		
	2000	2020	2030	2000	2020	2030
South Asia						
Bangladesh	11.8 (9.0–14.9)	34.5 (29.1–40.5)	49.8 (39.6–60.5)	25.9 (21.3–30.9)	50.7 (45.0–56.6)	63.8 (54.7–72.5)
India	23.3 (15.9–32.8)	69.5 (60.2–77.0)	85.2 (73.0–92.1)	20.2 (13.6–28.3)	31.5 (24.3–40.0)	39.1 (23.8–57.4)
Nepal	18.6 (14.6–23.4)	60.2 (53.3–66.7)	76.4 (65.3–84.8)	16.4 (13.2–19.9)	45.5 (39.1–52.8)	61.8 (49.7–73.9)
Pakistan	11.8 (0.5–53.6)	59.8 (50.6–68.4)	80.7 (63.4–91.9)	3.5 (0.0–20.4)	33.5 (25.2–42.6)	66.2 (42.1–85.4)
Southeast Asia						
Cambodia	18.7 (14.6–23.2)	90.9 (85.6–94.4)	97.9 (95.7–99.1)	10.0 (7.8–12.9)	74.7 (64.6–83.4)	92.1 (84.5–96.7)
Laos	13.6 (10.8–17.5)	66.4 (58.0–74.1)	86.8 (78.9–92.5)	6.9 (5.2–8.7)	33.3 (25.9–42.4)	56.6 (42.4–71.5)
Myanmar	47.6 (39.6–55.7)	58.1 (43.3–71.1)	63.4 (29.4–88.7)	12.8 (9.8–16.5)	25.5 (14.2–39.8)	35.0 (6.5–75.5)
Timor-Leste	24.3 (2.3–76.4)	61.7 (48.1–74.0)	79.0 (52.6–93.7)	18.2 (0.6–73.1)	45.4 (31.0–60.3)	70.0 (35.4–91.5)
Vietnam	29.5 (14.7–48.9)	68.0 (56.7–77.6)	80.3 (63.4–91.6)	30.8 (14.1–51.3)	25.9 (17.7–35.4)	29.1 (12.8–49.8)
Central Africa						
Angola	18.5 (12.4–25.4)	45.2 (34.5–56.5)	57.4 (41.0–72.5)	5.3 (3.1–8.1)	27.7 (19.5–37.2)	47.0 (30.6–64.2)
Cameroon	45.9 (38.6–52.7)	68.8 (62.7–74.1)	72.0 (55.6–83.8)	24.3 (19.8–29.2)	55.8 (49.7–61.5)	64.2 (46.9–77.8)
CAF	36.8 (30.5–43.6)	57.2 (49.7–64.2)	69.9 (49.8–85.2)	8.9 (6.8–11.1)	17.2 (13.6–21.4)	34.7 (15.4–57.2)
Chad	15.4 (12.3–19.3)	21.2 (14.0–29.1)	21.6 (12.6–33.1)	5.8 (4.6–7.4)	8.5 (5.4–12.5)	10.5 (6.3–16.2)
Congo	64.4 (37.6–84.8)	74.4 (59.7–85.9)	81.2 (56.0–94.8)	38.5 (16.0–64.9)	40.3 (23.1–61.7)	52.7 (19.7–85.7)
DRC	3.5 (2.6–4.7)	32.6 (25.5–40.2)	19.5 (9.7–32.5)	3.5 (2.7–4.5)	14.6 (11.0–18.8)	6.9 (3.4–12.1)
Sao TP	71.8 (65.8–77.0)	94.9 (93.4–96.1)	98.1 (97.1–98.8)	28.7 (23.5–34.1)	82.2 (78.2–85.6)	94.3 (91.9–96.3)
East Africa						
Burundi	44.1 (27.1–62.2)	93.0 (90.4–95.1)	97.0 (93.9–98.7)	11.7 (5.6–21.6)	76.9 (69.9–83.0)	88.6 (77.1–95.2)
Comoros	46.2 (38.7–53.8)	73.3 (52.0–88.0)	80.8 (51.1–95.8)	14.2 (10.8–18.2)	45.5 (20.7–71.6)	62.8 (22.8–92.1)
Ethiopia	10.7 (8.1–13.8)	26.3 (21.1–31.9)	39.5 (26.8–53.7)	10.7 (8.2–13.6)	31.1 (25.8–36.5)	54.4 (40.6–68.1)
Kenya	52.3 (45.5–58.8)	77.1 (66.3–86.7)	85.2 (66.3–96.2)	44.1 (38.1–50.2)	72.7 (59.2–83.3)	84.5 (61.6–96.0)
Madagascar	27.2 (21.8–33.2)	49.9 (41.3–58.5)	60.7 (47.2–73.9)	22.2 (17.5–26.9)	38.1 (30.0–46.3)	46.3 (33.3–59.5)
Malawi	24.6 (19.9–29.3)	82.8 (77.7–87.4)	92.6 (84.7–96.9)	16.4 (13.6–19.6)	57.4 (48.6–65.9)	73.6 (52.7–88.0)
Mozambique	56.0 (43.4–67.7)	78.3 (55.5–92.7)	79.8 (41.4–97.4)	12.7 (7.7–19.2)	39.3 (16.2–68.7)	54.9 (15.3–91.0)
Rwanda	67.1 (60.6–73.6)	95.5 (93.0–97.3)	98.2 (96.0–99.3)	45.1 (38.4–51.9)	85.5 (79.0–90.6)	92.9 (85.4–97.1)
Tanzania	52.7 (24.4–79.7)	64.0 (53.5–73.7)	64.4 (43.1–81.4)	24.0 (6.0–53.7)	57.7 (45.9–67.5)	72.0 (52.6–86.2)
Uganda	27.6 (20.5–35.3)	79.6 (71.1–86.6)	90.1 (76.7–96.7)	15.2 (10.9–20.3)	48.6 (35.3–61.0)	65.3 (34.6–87.0)
Zambia	50.6 (42.4–58.8)	81.8 (76.2–86.0)	90.0 (82.8–94.5)	9.4 (7.0–12.2)	50.6 (43.3–57.6)	76.0 (63.7–85.7)
Zimbabwe	18.0 (9.8–28.3)	90.1 (87.4–92.3)	96.7 (94.6–98.2)	2.9 (1.4–5.1)	69.8 (63.9–75.4)	92.0 (87.0–95.6)
West Africa						
Benin	74.0 (58.1–85.5)	78.7 (72.6–83.7)	74.2 (59.7–86.2)	28.9 (16.7–44.9)	37.6 (30.3–45)	45.2 (28.1–62.6)
Burkina Faso	39.4 (25.2–55.2)	84.9 (68.7–93.9)	90.0 (66.6–98.2)	10.5 (5.2–18.6)	51.4 (27.7–75.4)	69.1 (29.8–94.2)
Cote d’Ivoire	63.7 (56.4–69.9)	68.3 (58.1–77.5)	70.8 (46.5–87.9)	22.3 (17.4–27.2)	26.7 (18.3–36.7)	27.7 (9.4–53.5)
The Gambia	58.3 (51.1–65.1)	75.7 (71.1–79.9)	81.1 (70.7–89.8)	10.4 (8.1–13.4)	37.8 (32.5–43.3)	62.3 (44.5–76.7)
Ghana	46.1 (29.5–64.3)	79.4 (74.6–83.7)	89.0 (83.5–93.1)	19.2 (8.4–35.9)	64.3 (58.1–69.8)	83.7 (76.3–89.1)
Guinea	42.9 (27.0–60.3)	64.6 (54.0–73.8)	67.9 (44.5–84.6)	17.9 (8.0–33.4)	20.0 (13.7–27.4)	21.6 (7.7–42.8)
Liberia	23.3 (15.8–32.4)	71.2 (64.6–76.8)	82.8 (71.2–91.3)	7.7 (5.1–11.1)	36.1 (30.2–42.4)	56.3 (34.8–75.7)
Mali	28.2 (20.2–37.4)	49.4 (41.0–57.6)	52.4 (37.2–66.4)	11.6 (7.0–17.4)	26.1 (20.3–32.3)	30.1 (19.3–43.6)
Niger	38.4 (31.0–46.1)	46.3 (32.8–60.2)	52.7 (31.4–73.3)	18.2 (14.3–22.6)	22.5 (14.1–32.5)	30.0 (15.5–48.6)
Nigeria	8.8 (4.7–14.1)	48.8 (42.3–55.1)	80.1 (68.0–88.5)	3.8 (2.2–5.8)	29.9 (24.9–35.4)	64.2 (49.9–76.5)
Senegal	39.1 (27.8–51.8)	70.3 (65.6–74.4)	80.4 (72.5–86.4)	17.6 (11.0–26.3)	39.9 (35.1–44.8)	51.2 (40.8–61.7)
Sierra Leone	50.7 (43.7–57.8)	86.6 (83.9–89.1)	94.7 (92.4–96.6)	19.4 (15.8–23.8)	37.8 (32.9–42.9)	58.8 (48.9–68.5)
Togo	62.0 (55.7–67.6)	76.1 (69.5–81.7)	78.7 (64.0–89.5)	16.4 (13.3–19.8)	68.9 (60.4–76.1)	97.6 (93.7–99.4)

CAF, Central African Republic; DRC, Democratic Republic of the Congo; Sao TP, Sao Tome and Principe.

The predicted national coverage of age-appropriate polio3 and measles vaccinations from 2000 to 2030 is presented in Table 2. None of the countries included in this study achieved 90% coverage of age-appropriate polio3 or measles vaccinations by 2020. If the current trends continue, only three countries (Cambodia, Sao Tome and Principe, as well as Rwanda) will achieve over 90% coverage of the polio3 vaccine by 2030. For MCV, none of the countries was predicted to achieve the target by 2030. The highest annual rate of increase during 2000–2020 was in Zimbabwe (14.5%) for polio3 and Pakistan (13.4%) for measles. The lowest annual rate of change was observed in Vietnam for both polio3 and MCV (Supplementary Table S4).

**Table 2:** National level coverage of age-appropriate polio3 and MCV vaccine in 41 LMICs in Asia and Sub-Saharan Africa, 2000–2030.

Country	Predicted coverage (95% credible intervals)					
	polio3			MCV		
	2000	2020	2030	2000	2020	2030
South Asia						
Bangladesh	26.2 (21.8–30.9)	50.2 (44.5–55.7)	62.4 (53.6–70.1)	46.3 (40.3–52.6)	65.6 (60.0–70.6)	74.8 (66.9–81.4)
India	26.2 (18.6–34.8)	30.3 (22.8–38.2)	34.0 (21.2–48.8)	22.0 (15.2–30.0)	41.7 (32.9–50.8)	53.3 (36.8–69.1)
Nepal	29.9 (24.9–35.1)	45.6 (39.5–51.8)	54.4 (43.2–65.5)	17.0 (13.7–21.0)	63.4 (57.4–69.4)	81.8 (74.5–88.1)
Pakistan	6.8 (0.6–25.0)	40.4 (32.2–48.0)	68.2 (49.2–83.6)	3.2 (0.1–15.8)	39.4 (31.0–48.0)	69.8 (48.9–85.9)
Southeast Asia						
Cambodia	11.4 (9.1–13.9)	73.7 (64.5–81.4)	91.0 (84.3–95.6)	14.8 (11.8–18.3)	69.7 (58.8–79.0)	86.8 (76.1–93.6)
Laos	8.1 (6.3–10.2)	37.4 (30.6–45.2)	60.8 (48.8–72.4)	8.3 (6.4–10.4)	42.0 (33.9–50.3)	65.8 (52.2–76.9)
Myanmar	14.3 (11.2–18.0)	27.8 (18.8–37.8)	37.1 (14.3–63.0)	32.7 (26.9–39.1)	36.4 (25.4–48.7)	40.6 (15.8–70.6)
Timor-Leste	12.0 (1.3–41.2)	39.3 (28.4–51.1)	61.5 (34.3–84.2)	18.5 (1.8–60.3)	47.2 (35.5–59.7)	65.2 (37.9–86.5)
Vietnam	30.5 (17.3–46.9)	33.3 (24.3–42.2)	37.8 (21.2–55.9)	55.3 (36.2–75.5)	46.8 (35.6–57.5)	44.7 (24.1–65.3)
Central Africa						
Angola	7.1 (5.0–9.4)	25.9 (18.9–34.1)	39.8 (26.0–54.8)	6.4 (4.3–9.1)	26.9 (19.3–35.6)	41.7 (27.1–56.7)
Cameroon	22.8 (18.9–26.9)	60.0 (54.2–65.5)	63.7 (48.1–76.4)	33.6 (28.1–39.7)	50.3 (44.5–56.4)	49.9 (33.7–66.1)
CAF	8.7 (7.0–10.7)	24.9 (20.0–30.2)	42.8 (26.5–62.0)	11.6 (9.1–14.4)	17.1 (13.3–21.2)	24.5 (13.1–39.2)
Chad	7.1 (5.7–8.8)	12.3 (8.2–17.4)	16.9 (10.7–24.6)	5.0 (3.9–6.2)	14.1 (9.4–19.4)	20.1 (13.0–28.5)
Congo	27.9 (11.2–49.9)	39.9 (25.2–56.3)	46.6 (19.3–76.1)	16.4 (5.6–32.4)	38.2 (24.0–55.0)	43.3 (17.1–73.8)
DRC	3.2 (2.5–4.1)	27.7 (22.0–33.8)	21.9 (12.4–34.7)	3.6 (2.7–4.6)	13.7 (10.2–17.8)	6.1 (2.9–11.0)
Sao TP	26.0 (21.5–30.7)	81.1 (77.0–84.6)	94.0 (91.6–95.9)	37.0 (30.9–43.3)	71.6 (66.2–76.4)	84.0 (78.2–88.7)
East Africa						
Burundi	23.3 (13.7–34.0)	74.1 (67.6–79.9)	85.5 (75.3–92.4)	33.3 (20.0–49.6)	77.1 (69.8–82.6)	83.4 (69.7–92.1)
Comoros	13.7 (10.8–17.0)	46.0 (27.8–67.0)	64.1 (33.4–88.9)	22.1 (17.3–27.5)	38.2 (20.2–59.3)	47.0 (17.5–78.4)
Ethiopia	18.1 (14.5–22.1)	32.0 (26.9–37.7)	48.7 (36.2–61.9)	11.2 (9.0–14.1)	26.7 (22.1–32.1)	44.4 (31.1–58.6)
Kenya	36.6 (31.6–42.0)	69.6 (58.9–79.6)	80.4 (62.1–92.9)	39.4 (33.6–45.2)	53.1 (39.9–65.0)	58.9 (31.4–81.2)
Madagascar	20.7 (16.8–24.8)	42.3 (35.0–50.0)	52.9 (40.6–65.1)	27.3 (22.3–32.5)	35.0 (27.7–42.7)	37.5 (26.0–49.9)
Malawi	16.3 (13.7–19.2)	51.8 (44.1–59.4)	69.3 (52.4–83.3)	27.0 (22.7–31.8)	52.0 (44.2–60.5)	62.3 (44.7–79.2)
Mozambique	12.2 (8.0–17.6)	39.8 (17.7–64.2)	56.3 (17.2–88.2)	35.5 (25.0–46.7)	50.8 (27.8–73.1)	53.6 (19.0–85.4)
Rwanda	46.6 (40.6–52.9)	84.8 (79.4–89.6)	91.9 (85.2–96.4)	51.1 (44.5–57.5)	68.9 (47.8–85.4)	72.4 (37.6–93.4)
Tanzania	24.2 (7.7–49.0)	55.7 (45.2–65.2)	68.0 (49.7–82.7)	35.0 (13.2–63.1)	52.6 (42.5–62.9)	58.0 (39.0–75.8)
Uganda	17.4 (13.4–22.3)	44.8 (35.0–55.0)	58.7 (36.6–78.7)	20.3 (14.8–25.9)	45.4 (34.8–56.3)	55.7 (30.5–77.8)
Zambia	10.4 (8–13.1)	47.4 (40.7–54.2)	71.2 (59.9–80.3)	33.4 (26.9–40.5)	54.3 (47.4–61.2)	65.6 (53.3–77.4)
Zimbabwe	4.5 (2.3–7.6)	67.0 (61.3–72.2)	89.8 (84.3–93.8)	12.0 (6.7–19.5)	68.1 (62.1–73.5)	84.2 (76.0–90.4)
West Africa						
Benin	33.1 (20.6–46.8)	38.6 (32.7–45.1)	44.0 (31.1–58.4)	27.9 (16.4–42.1)	47.6 (40.5–54.6)	52.4 (36.3–68.2)
Burkina Faso	11.1 (6.9–16.5)	51.9 (33.5–70.3)	70.4 (40.0–90.5)	16.9 (9.4–26.6)	67.7 (44.2–85)	78.3 (43.1–95.6)
Cote d’Ivoire	22.8 (18.3–27.4)	35.5 (26.6–45.1)	39.2 (20.9–60.0)	31.9 (26.0–37.7)	33.7 (24.9–43.1)	33.0 (15.2–54.5)
The Gambia	13.4 (10.5–16.5)	37.1 (32.4–42.2)	55.4 (42.1–69.3)	42.4 (35.8–49.1)	58.5 (53.3–63.6)	65.1 (50.1–77.7)
Ghana	24.2 (14.9–35.6)	58.7 (52.5–65.1)	74.2 (65.5–81.9)	32.3 (19.8–47.1)	58.0 (51.7–64.0)	68.1 (57.7–77.2)
Guinea	18.0 (9.9–28.8)	26.9 (20.5–34.5)	33.1 (17.4–51.8)	21.0 (11.7–32.8)	20.4 (14.3–27.2)	20.5 (9.1–36.5)
Liberia	10.8 (7.7–15.1)	35.5 (30.1–41.3)	50.8 (34.2–66.3)	11.7 (8.1–16.4)	39.3 (33.2–45.5)	54.2 (37.1–71.5)
Mali	14.6 (10.4–19.7)	24.3 (19.1–30.1)	26.5 (17.8–36.8)	13.1 (8.6–18.2)	24.7 (19.3–30.1)	23.4 (14.3–34.3)
Niger	18.8 (15.1–23.0)	26.4 (17.5–36.7)	33.6 (18.8–50.7)	25.5 (20.5–31.2)	28.7 (18.5–40.3)	34.1 (18.1–52.7)
Nigeria	6.3 (3.8–9.5)	33.4 (28.6–38.6)	63.0 (50.7–75.3)	5.4 (3.3–8.2)	28.8 (24.1–34.1)	55.6 (40.8–69.1)
Senegal	18.9 (12.6–26.4)	39.3 (34.6–43.8)	50.4 (40.4–59.7)	23.7 (16.6–33.2)	52.2 (47.2–56.8)	63.4 (53.7–71.9)
Sierra Leone	27.3 (22.7–32.3)	36.8 (32.3–41.3)	49.5 (40.7–58.1)	23.5 (19.4–28.0)	45.6 (40.8–50.3)	63.3 (54.0–71.5)
Togo	18.3 (15.2–21.8)	61.3 (54.3–68.0)	86.6 (76.2–93.9)	21.5 (17.6–25.5)	50.4 (42.9–58.1)	62.1 (44.9–78.1)

CAF, Central African Republic; DRC, Democratic Republic of the Congo; Sao TP, Sao Tome and Principe.

### 3.4 Urban–rural disparities in vaccination coverage

**Figure 2:**
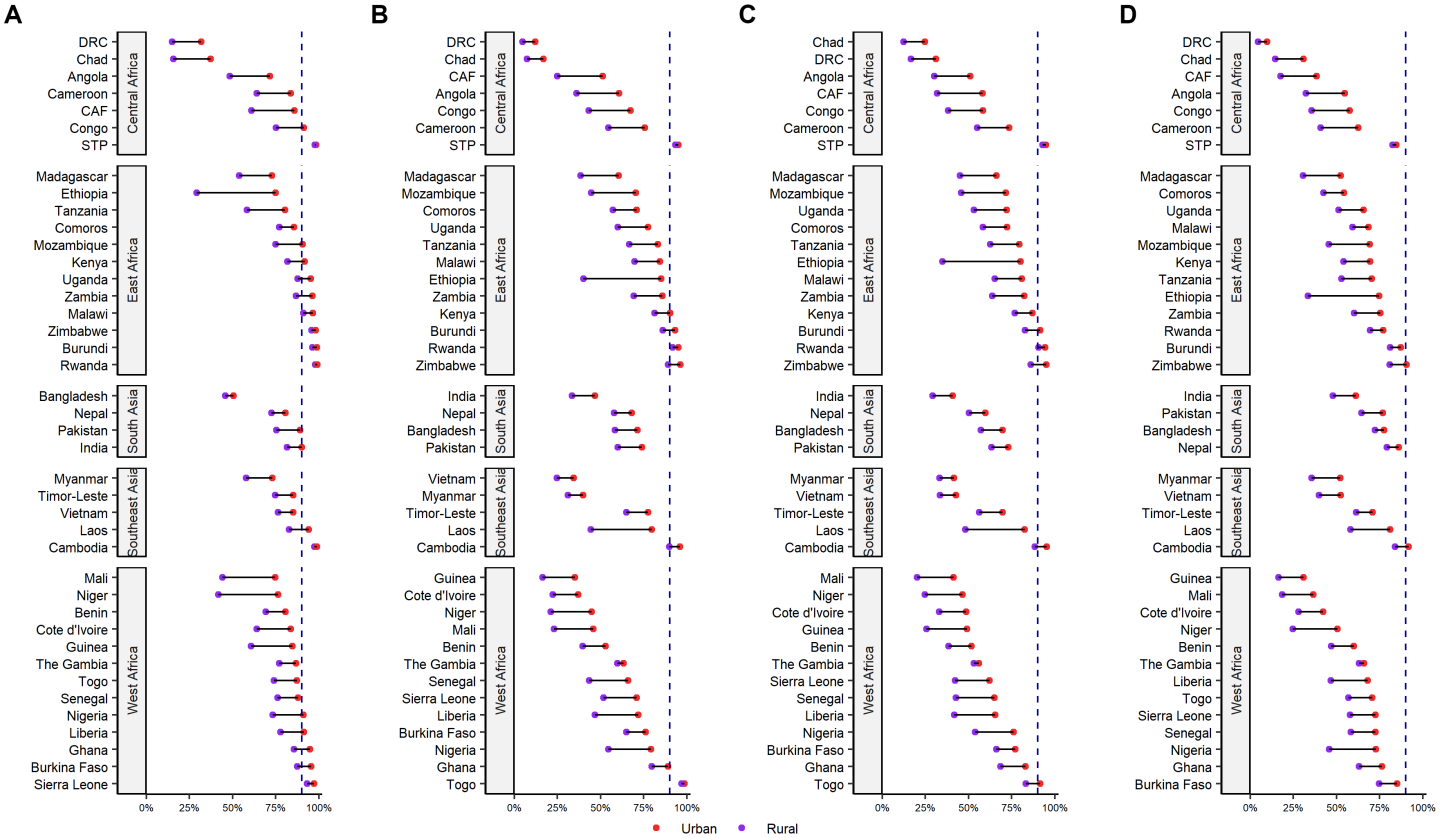
Coverage of age-appropriate vaccinations differentiated by the urban/rural place of residence in 41 LMICs in Asia and Sub-Saharan Africa, 2030. CAF, Central African Republic; DRC, Democratic Republic of the Congo; Sao TP, Sao Tome and Principe. **(A)** BCG **(B)** DPT3 **(C)** Polio3 **(D)** MCV.

Figure 2 presents the urban–rural specific coverage of age-appropriate BCG, DTP3, polio3, and MCV vaccines for all 41 LMICs by 2030. Overall, the coverage of all age-appropriate vaccines in urban areas was substantially higher than in rural areas. By 2030, a wider urban–rural disparity in BCG vaccination is observed in the Central African Republic, Ethiopia, Guinea, Niger, and Mali (Figure 2A; Supplementary Table S5). If the current trend continues, the coverage of BCG vaccination in both urban and rural areas is predicted to reach 90% coverage in nine countries by 2030 (Figure 2A). Meanwhile, a comparatively narrower coverage gap of DTP3 vaccine is expected in Sao Tome and Principe, Togo, Rwanda, and Cambodia, whereas a wider coverage gap is expected in Ethiopia, Laos, and the Central African Republic (Figure 2B; Supplementary Table S6). No country is projected to achieve 90% coverage of polio3 vaccines for both urban and rural areas by 2030 (Figure 2C; Supplementary Table S7). A wide urban–rural disparity in the coverage of MCV is observed in Ethiopia, Nigeria, Niger, and Mozambique in 2030 (Figure 2D; Supplementary Table S8).

### 3.5 Wealth-based inequalities In vaccination coverage

The coverage of age-appropriate vaccines by wealth index is presented in Table 3 and Supplementary Tables S9–S12. If the present trends continue, by 2030, the coverage of BCG vaccine in the richest quintile is predicted to be 90% or higher in 24 countries, whereas only seven countries are on track to achieve a 90% coverage target for the poorest quintile (Table 3; Supplementary Table S9). Similarly, the coverage of the DTP3 vaccine for both the poorest and richest quintiles is projected to be over 90% in three countries (Rwanda, Sao Tome and Principe, and Togo) by 2030 (Table 3; Supplementary Table S10). None of the countries from Southeast Asia, South Asia, or West Africa will achieve 90% coverage of polio3 vaccine among the poorest quintile by 2030 (Table 3; Supplementary Table S11). For the MCV, only five countries (Nepal, Cambodia, Burundi, Rwanda, and Burkina Faso) will have more than 90% coverage of MCV among the richest quintile by 2030, while none is predicted to be 90% or higher among the poorest quintile if recent trends continue (Table 3; Supplementary Table S12).

**Table 3:** Coverage of age-appropriate vaccines between poorest and richest quintiles in 41 LMICs in Asia and Sub-Saharan Africa, 2030.

Country	Predicted coverage (95% credible intervals)							
	BCG		DTP3		polio3		MCV	
	Poorest	Richest	Poorest	Richest	Poorest	Richest	Poorest	Richest
South Asia								
Bangladesh	46.7 (36.7–56.5)	49.3 (39.4–58.6)	57.3 (47.0–66.9)	79.9 (73.1–86.0)	56.1 (44.4–66.9)	79.3 (71.3–86.1)	69.6 (59.5–78.3)	84.9 (78.2–89.9)
India	82.0 (70.6–90.6)	95.7 (91.7–98.0)	31.5 (16.6–48.9)	67.5 (48.2–81.5)	27.2 (12.9–44.4)	59.1 (37.1–76.6)	46.6 (28.6–64.9)	79.1 (65.2–89.3)
Nepal	68.0 (56.5–78.0)	88.3 (82.3–92.6)	52.9 (39.6–65.5)	76.5 (65.1–84.8)	50.5 (39.0–62.2)	71.4 (61.1–79.8)	78.9 (70.3–85.3)	93.0 (89.7–95.5)
Pakistan	70.1 (49.2–86.4)	95.8 (91.1–98.5)	48.7 (24.7–72.4)	83.1 (66.5–93.8)	62.1 (35.1–83.6)	85.9 (68.0–95.3)	48.7 (26.3–70.4)	85.6 (71.2–94.1)
Southeast Asia								
Cambodia	96.2 (92.0–98.4)	99.1 (98.2–99.6)	84.3 (70.7–93.8)	96.9 (93.4–98.9)	89.2 (81.7–94.1)	97.8 (96.0–98.9)	88.9 (81.7–94.2)	97.5 (95.5–98.8)
Laos	81.1 (69.6–88.9)	96.1 (93.1–98.0)	32.3 (19.1–47.0)	81.3 (70.0–89.8)	38.0 (25.9–51.7)	83.1 (74.0–89.8)	56.5 (42.6–69.4)	88.1 (81.1–92.8)
Myanmar	56.8 (21.5–86.0)	78.0 (46.7–95.0)	32.8 (5.5–76.9)	49.0 (11.7–87.5)	27.8 (16.1–42.0)	47.0 (30.2–63.0)	30.1 (19.0–43.4)	59.2 (43.9–72.2)
Timor-Leste	68.5 (37.8–89.8)	91.9 (78.6–98.2)	52.2 (17.9–85.9)	81.3 (52.6–96.6)	46.4 (20.7–73.3)	75.7 (50.1–92.3)	51.0 (26.5–73.4)	77.8 (56.0–90.9)
Vietnam	74.7 (55.3–88.2)	92.0 (84.1–96.9)	19.5 (7.6–38.2)	38.3 (18.1–63.8)	24.3 (10.5–44.6)	41.8 (20.3–65.8)	43.2 (23.0–64.5)	69.3 (47.9–85.2)
Central Africa								
Angola	53.9 (35.3–71.2)	90.5 (82.5–95.6)	40.2 (23.7–58.1)	84.4 (72.0–92.6)	33.9 (19–50.6)	72.4 (54.5–85.0)	37.9 (24.3–52.7)	82.0 (70.6–90.0)
Cameroon	48.7 (31.8–65.0)	87.3 (78.4–93.3)	42.0 (26.3–58.9)	82.4 (70.1–90.6)	62.0 (50.9–72.3)	88.9 (84.0–92.8)	40.2 (29.7–50.7)	81.1 (73.8–87.2)
CAF	52.2 (30.3–72.0)	88.9 (78.0–95.4)	19.0 (7.6–36.5)	54.6 (31.3–76.6)	21.1 (13.3–30.4)	55.2 (41.9–68.4)	10.0 (6.5–14.9)	40.4 (28.5–52.4)
Chad	12.0 (7.3–18.6)	41.8 (28.9–54.2)	5.9 (3.3–9.4)	19.9 (12.4–29.4)	9.9 (5.3–16.7)	27.4 (15.9–41.5)	13.9 (8.2–22.3)	40.3 (27.5–54.9)
Congo	71.0 (36.2–93.4)	95.7 (86.0–99.3)	36.5 (9.2–75.9)	73.7 (35.1–95.4)	24.9 (12.7–40.5)	55.8 (36.9–74.0)	29.9 (16.5–45.9)	69.2 (51.5–82.9)
DRC	12.5 (6.6–20.9)	48.1 (31.8–64.5)	5.4 (2.5–9.7)	25.2 (14.0–39.2)	73.6 (60.2–82.9)	92.3 (87.3–95.5)	43.1 (31.7–55.3)	79.6 (70.4–86.9)
Sao TP	97.7 (96.6–98.5)	98.7 (9.08–99.2)	91.6 (88.0–94.2)	97.5 (96.1–98.4)	91.0 (86.7–94.4)	97.1 (95.4–98.3)	78.1 (69.1–85.2)	89.2 (83.6–93.2)
East Africa								
Burundi	97.0 (93.2–98.9)	99.0 (97.7–99.6)	85.8 (72.0–94.4)	93.2 (85.1–97.6)	88.2 (79.5–93.8)	95.0 (91.3–97.6)	86.6 (76.5–93.2)	93.1 (87.9–96.7)
Comoros	73.3 (34.2–95.3)	89.1 (62.1–98.6)	50.2 (13.5–89.7)	77.5 (39.0–97.2)	61.9 (41.1–79.4)	86.0 (72.5–94.3)	42.3 (23.8–60.4)	74.9 (58.1–87.3)
Ethiopia	24.5 (14.9–35.7)	75.3 (63.1–84.8)	41.2 (28.9–53.7)	89.8 (83.9–94.0)	24.9 (17.8–33.2)	77.4 (69.0–84.3)	25.9 (18.8–35.0)	74.8 (65.6–82.3)
Kenya	89.8 (73.1–97.8)	98.0 (94.2–99.6)	85.3 (62.1–97.0)	96.5 (89.2–99.3)	78.5 (62.5–89.3)	93.7 (87.3–97.5)	52.7 (34.3–69.3)	82.1 (69.1–90.8)
Madagascar	48.3 (33.7–61.5)	84.4 (75.1–90.7)	32.4 (21.4–45.6)	71.4 (59.0–82.1)	42.1 (28.6–55.7)	76.8 (65.3–86.3)	32.7 (22.0–44.9)	74.3 (63.6–83.4)
Malawi	89.4 (76.7–96.1)	96.0 (90.5–98.6)	66.1 (42.9–86.8)	84.9 (69.3–95.2)	62.6 (50.4–73.1)	83.2 (75.6–89.3)	59.9 (47.5–71.0)	75.2 (65.0–83.1)
Mozambique	68.4 (22.4–95.0)	93.4 (70.0–99.4)	33.5 (3.6–78.9)	73.6 (26.2–96.9)	18.5 (6.0–38.4)	59.4 (30.7–81.8)	39.8 (18.6–63.6)	79.7 (60.0–92.2)
Rwanda	98.4 (96.7–99.3)	99.4 (98.8–99.8)	92.1 (83.3–97.0)	96.3 (91.9–98.6)	92.5 (87.1–96.3)	96.8 (94.1–98.5)	82.8 (67.3–92.8)	90.9 (81.1–96.4)
Tanzania	52.0 (31.8–71.7)	86.0 (73.7–93.6)	60.7 (38.5–80.0)	84.8 (70.9–93.7)	55.6 (35.2–73.0)	80.2 (65.2–90.3)	48.3 (31.3–65.7)	76.8 (63.2–87.8)
Uganda	90.4 (75.8–97.6)	94.9 (87.0–98.7)	62.2 (31.9–88.3)	80.9 (55.3–95.4)	59.6 (44.9–72.3)	80.5 (70.6–88.5)	60.3 (46.8–73.5)	78.0 (66.5–86.6)
Zambia	86.4 (77.4–92.7)	97.9 (96.3–99.0)	67.0 (52.9–80.4)	91.0 (85.3–95.5)	62.6 (49.1–73.8)	88.5 (82.7–93.3)	57.9 (46.0–69.6)	83.2 (74.7–89.2)
Zimbabwe	95.2 (92.0–97.3)	98.7 (97.8–99.3)	86.1 (78.0–92.4)	97.2 (95.2–98.5)	99.8 (99.7–99.9)	100 (99.9–100)	74.2 (61.6–84.2)	91.2 (84.9–95.2)
West Africa								
Benin	60.4 (41.7–75.9)	88.3 (79.0–94.3)	33.4 (17.9–51.9)	65.1 (46.1–80.3)	31.2 (18.1–46.5)	63.7 (45.4–78.0)	45.5 (30.4–61.1)	76.8 (64.5–86.1)
Burkina Faso	88.5 (61.3–98.7)	97.3 (89.0–99.7)	66.0 (22.3–94.7)	81.4 (41.9–97.9)	76.3 (51.2–92.2)	89.1 (74.0–97.1)	89.2 (73.6–96.7)	96.2 (90.2–99.0)
Cote d’Ivoire	61.3 (32.8–82.4)	89.6 (74.5–96.8)	21.0 (6.4–48.1)	51.8 (23.3–80.7)	37.0 (23.8–53.2)	69.3 (55.0–81.7)	28.7 (17.7–42.5)	60.6 (45.8–74.9)
The Gambia	78.8 (64.9–88.6)	92.4 (86.1–96.2)	58.6 (41.5–74.5)	68.2 (52.4–81.5)	49.3 (39.3–59.6)	60.9 (50.5–70.9)	63.5 (53.9–71.7)	73.6 (66.0–81.1)
Ghana	84.8 (77.9–89.8)	96.9 (95.3–98.0)	78.4 (69.4–85.3)	93.2 (89.8–95.7)	67.6 (56.1–78.2)	90.2 (84.8–94.1)	62.3 (50.6–72.7)	85.0 (78.1–90.4)
Guinea	49.4 (25.2–71.2)	86.4 (71.8–94.8)	10.1 (2.8–23.5)	39.6 (15.0–66.9)	16.2 (8.4–27.9)	49.8 (31.9–67.8)	12.5 (6.5–21.1)	39.2 (23.8–56.3)
Liberia	69.7 (50.7–84.2)	93.4 (87.0–97.0)	39.9 (21.4–61.7)	78.8 (61.5–91.0)	43.9 (29.0–58.6)	78.9 (67.8–88.0)	48.7 (34.9–62.9)	82.5 (73.0–89.7)
Mali	35.9 (22.9–49.3)	80.3 (69.7–88.1)	20.3 (12.1–30.6)	55.2 (40.5–70.1)	30.1 (20.4–41.5)	66.7 (54.7–76.9)	36.0 (26.0–47.0)	72.3 (62.1–81.3)
Niger	35.2 (19.1–56.2)	78.2 (62.3–90.4)	18.2 (7.9–32.1)	47.7 (28.0–67.7)	23.6 (11.5–40.1)	52.7 (33.6–73.1)	27.9 (14.8–45.2)	65.7 (47.4–81.9)
Nigeria	62.9 (47.1–76.5)	96.6 (93.9–98.3)	36.9 (24.1–50.4)	87.3 (79.9–92.8)	25.7 (17.6–35.3)	73.4 (63.3–81.3)	23.4 (16.9–31.6)	77.8 (68.8–84.9)
Senegal	72.5 (63.5–80.0)	91.1 (87.3–94.0)	38.2 (28.3–48.7)	75.9 (67.5–83.5)	40.8 (31.1–50.5)	76.9 (69.3–83.3)	57.4 (47.9–67.0)	82.6 (76.3–88.2)
Sierra Leone	94.2 (91.8–96.0)	98.4 (97.6–98.9)	56.8 (47.3–66.9)	82.9 (76.1–88.3)	43.0 (33.2–53.5)	72.1 (63.3–80.1)	58.7 (49.4–68.2)	79.7 (72.7–86.0)
Togo	68.4 (48.8–84.0)	89.9 (80.5–95.8)	97.6 (94.1–99.4)	99.4 (98.5–99.9)	71.3 (59.5–81.0)	91.0 (85.8–94.6)	59.9 (47.9–71.8)	82.3 (74.4–88.6)

CAF, Central African Republic; DRC, Democratic Republic of the Congo; Sao TP, Sao Tome and Principe.

### 3.6 Scenario-based analysis

**Figure 3:**
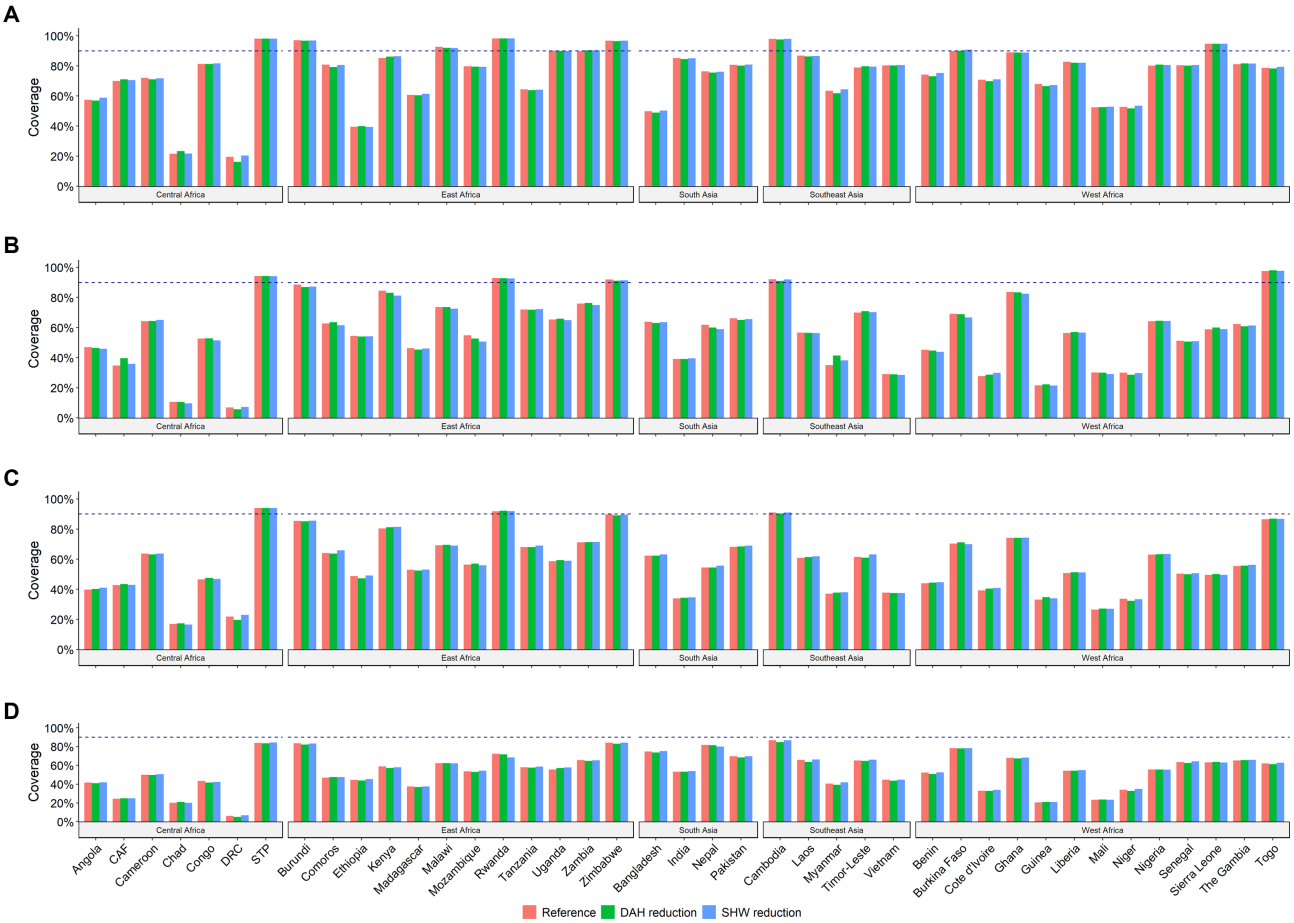
Projected coverage of age-appropriate vaccines in 2030 by different scenarios. Note: CAF, Central African Republic; DRC, Democratic Republic of the Congo; Sao TP, Sao Tome and Principe. **(A)** BCG **(B)** DPT3 **(C)** Polio3 **(D)** MCV.

The country-specific projected coverage of age-appropriate BCG, DTP3, polio3, and MCV vaccines in 2030 with alternative scenarios are presented in Figure 3 and Supplementary Tables S13–S16. In the DAH reduction scenario, none of the countries experienced a major change in age-appropriate vaccination coverage. More than a 2% reduction is predicted to be observed in the Democratic Republic of Congo for BCG and polio3, Mozambique for DTP3, and Cambodia and Laos for measles vaccines. In the SHW reduction scenario, no significant difference was predicted in vaccination coverage. However, a reduction of approximately 3% was observed in Mozambique and Kenya for DTP3 and Rwanda for measles by 2030.

### 3.7 Sensitivity analysis and model diagnostic

Excluding country-level predictors, the median absolute differences between the two sets of results were very small (Supplementary Tables S17, S18). After altering the prior distribution, no significant differences were observed in the results. The median absolute difference between the two sets of results was 0.0% from 2000 to 2030 (Supplementary Tables S19, S20). In the case of model diagnostics, the potential scale reduction factor (PSRF) values indicated the point estimate and upper limit of PSRF for age-appropriate vaccine models to be approximately 1 (Supplementary Tables S21, S22).

## 4 Discussion

This is the first study to provide a comprehensive assessment of age-appropriate vaccination coverage among children under 5 years in LMICs. The findings suggest that the coverage of age-appropriate vaccinations among children has increased substantially at the regional and national levels, as well as by area of residence and socioeconomic status in most countries between 2000 and 2030. The largest increase in vaccination coverage was observed in East Africa, followed by South and Southeast Asia. However, of the 41 LMICs, only 10 countries would achieve 90% coverage of the BCG vaccine by 2030, five of DTP3, three of polio3, and none of MCV vaccines. Wider coverage gaps between urban–rural and poorest-richest groups were observed in several African countries. However, a narrower coverage gap was found in Burundi, Cambodia, Rwanda, Sao Tome and Principe, as well as Sierra Leone.

Our study suggests that age-appropriate vaccination coverage is predicted to increase at national and regional levels between 2000 and 2030, corresponding to the initiation of EPI by the WHO and GVAP, as well as substantial financial support for vaccination programming generated by the introduction of Gavi, the Alliance vaccine, that helped in reducing VPDs and under-five mortality (25). Despite these investments and policies, our study predicted several countries from Africa and South Asia, to have relatively slower progress in age-appropriate vaccine coverage during 2020–2030. This finding is similar to WHO and UNICEF estimates of national immunization coverage (WUENIC) 2020, which showed that more than 60% of unvaccinated children live in 10 countries in the same regions (26). One possible reason could be the availability and acceptability dimensions, such as long distance to health facilities, immunization administration outside a health facility, vaccine hesitancy or refusal, and shortage of health workforce (27). Another reason for barriers to accessing vaccination in the listed countries within an appropriate time frame could be civil war, political instability, and ethnic conflict (28, 29). Hence, safe and better access to health facilities, greater provision of childhood immunization services, and improved attitudes and knowledge of mothers regarding immunization can enhance coverage and utilization of childhood immunization services in LMICs. Therefore, effective strategies must be adopted by stakeholders and policymakers to expand the coverage of age-appropriate vaccines, particularly for those living in rural and hard-to-reach areas.

Conversely, a high coverage of age-appropriate vaccines with relatively fewer inequalities has been observed in several countries, such as Burundi, Cambodia, Rwanda, Sao Tome and Principe, and Zimbabwe. In a short period of time, Bangladesh, Cambodia, Nepal, Rwanda, Gambia, and Zimbabwe have had successful vaccination coverage, which is attributable to a combination of different strategies such as multi-year national immunization plans, advocacy for better domestic funding for immunization, and evaluation of EPI activities including joint field missions, sensitization of health workers on VPD surveillance, tracking vaccination defaulters, and data quality improvement (30, 31). Cambodia, for instance, introduced a catch-up campaign aimed at children who had missed or had not received doses of vaccines for any reason (32). Thus, concerted efforts to mitigate accessibility through intensive community mobilization and expanding basic health services will effectively help narrow the inequality gaps in resource-constrained settings.

Immunization programs are highly vulnerable to unprecedented public health emergencies at the subnational, national, and/or global levels, which can hinder efforts by disrupting the normal function of nations and potentially derailing past gains. A recent study indicated that routine immunization services have decreased in early 2020 due to the COVID-19 pandemic (5). Inadequate human resources, shortage of vaccine supply, and strict lockdown may explain why (5, 33). Our scenario-based analysis elucidated that these reductions or disruptions are not projected to downgrade the trends of age-appropriate vaccination by 2030. Because, though the recent outbreak of COVID-19 hampered the immunization schedules, majority of the LMICs are now leveraging pre-existing program strengths and incorporating protocols for safer vaccine administration (5, 33). For instance, health workers in Cambodia were reported of providing “village to village” and “door to door” services to maintain routine immunization service and protect Cambodians from COVID-19 (34). Therefore, international organizations and national government must increase advocacy for ensuring timeliness of childhood vaccinations by overcoming resource mobilization, staff displacement, low health-seeking behavior of the public due to fear of getting the infection by visiting health facilities, etc.

This study has several strengths. First, this is the first comprehensive assessment of the trends and projections of age-appropriate vaccination coverage at the regional and national levels, as well as place of residence and wealth index in LMICs. Second, this study estimates the required rate of increase for a country to achieve a 90% target by 2030. Third, it used a large number of nationally representative surveys, which improved the model validity of the indicators. Lastly, it used robust statistical tools, such as the Bayesian model, which allowed better use of available data and provided better estimates, especially for low data points. The study also has some limitations, most information was from home-based records which can introduce several biases. Moreover, this study excluded children without vaccination card and older children have less percentage of card seen. There might be a possibility of selection bias. Thus, this study was unable to adjust for these biases. Previous studies have shown that the direction and magnitude of these biases vary substantially (35, 36). The projections were made on the assumption that there would be no major change in the existing health system, which may be too strict as countries might introduce new strategies or other forms of health financing to increase vaccination coverage. Therefore, these projections cannot account for the unknown forthcoming impact of national or international health policies or other emergencies. Lastly, this study focused only on the LMICs of South Asia, Southeast Asia, and Sub-Saharan Africa and is not representative of all countries in these regions due to the lack of information on the date of birth and vaccination. It also did not allow the model to adjust for the COVID-19 pandemic, ethnic conflict, and civil war issues due to lack of data, which could potentially affect the predictions.

## 5 Conclusion

Substantial improvements have been made in age-appropriate vaccination coverage in LMICs during the past decades. However, progress stalled from 2000 to 2020 in many countries. Except for the measles vaccine, only four countries (Cambodia, Sao Tome and Principe, Rwanda, and Zimbabwe) are predicted to meet 90% vaccination coverage targets for BCG, DTP3, and polio3 by 2030. Wide urban–rural and poor-rich disparities in age-appropriate vaccinations will remain in many countries, especially Ethiopia, Mali, Niger, and Nigeria. These findings will contribute to the improvement of age-appropriate vaccination coverage and support policymakers in developing necessary control strategies with respect to delayed vaccination. Improving vaccine availability, increasing interaction with community health workers, and decreasing the coverage gap in underserved children by introducing appropriate policies and strengthening the health system have the potential to improve child survival through enhanced vaccine coverage and timelines.

## Data availability statement

Publicly available datasets were analyzed in this study. This data can be found at: the data that support the findings of this study are publicly available from the DHS Program at https://dhsprogram.com/ and MICS at https://mics.unicef.org/. SDI, GSH, and DAH were extracted from the IHME database on the Global Health Data Exchange website (https://ghbx.healthdata.org) and SHW from the WHO Global Health Observatory Data Repository (https://www.int/data/gho). The data was extracted until September 2021. During this period, we extracted 174 databases from DHS and MICS.

## Ethics statement

This study was a secondary analysis; therefore, ethics approval and consent to participate was not required. The studies were conducted in accordance with the local legislation and institutional requirements. Written informed consent for participation in this study was provided by the participants' legal guardians/next of kin.

## Author contributions

MI: Writing – review & editing, Writing – original draft, Visualization, Validation, Supervision, Software, Resources, Project administration, Methodology, Investigation, Funding acquisition, Formal analysis, Data curation, Conceptualization. MMR: Writing – review & editing, Validation, Methodology, Funding acquisition. MSR: Writing – review & editing, Validation, Methodology. SA: Writing – review & editing. MA: Writing – review & editing, Methodology. MH: Writing – review & editing, Validation, Resources.

## Footnotes

^1^https://ghbx.healthdata.org

^2^https://www.int/data/gho
