# Supplementary material for: Trends and projections of age-appropriate vaccination coverage in 41 low- and middle- income countries in Asia and Sub-Saharan Africa, 2000–2030
Source: Front Public Health. 2024 May 9;12:1371258. doi: 10.3389/fpubh.2024.1371258 (PMC11111938; doi:10.3389/fpubh.2024.1371258)
Supplement: Supplementary file 1 [file Data_Sheet_1.docx]

# **Supplementary Material**

**Trends and projections of age-appropriate vaccination coverage in 41 low- and middle- income countries in Asia and Sub-Saharan Africa, 2000-2030**


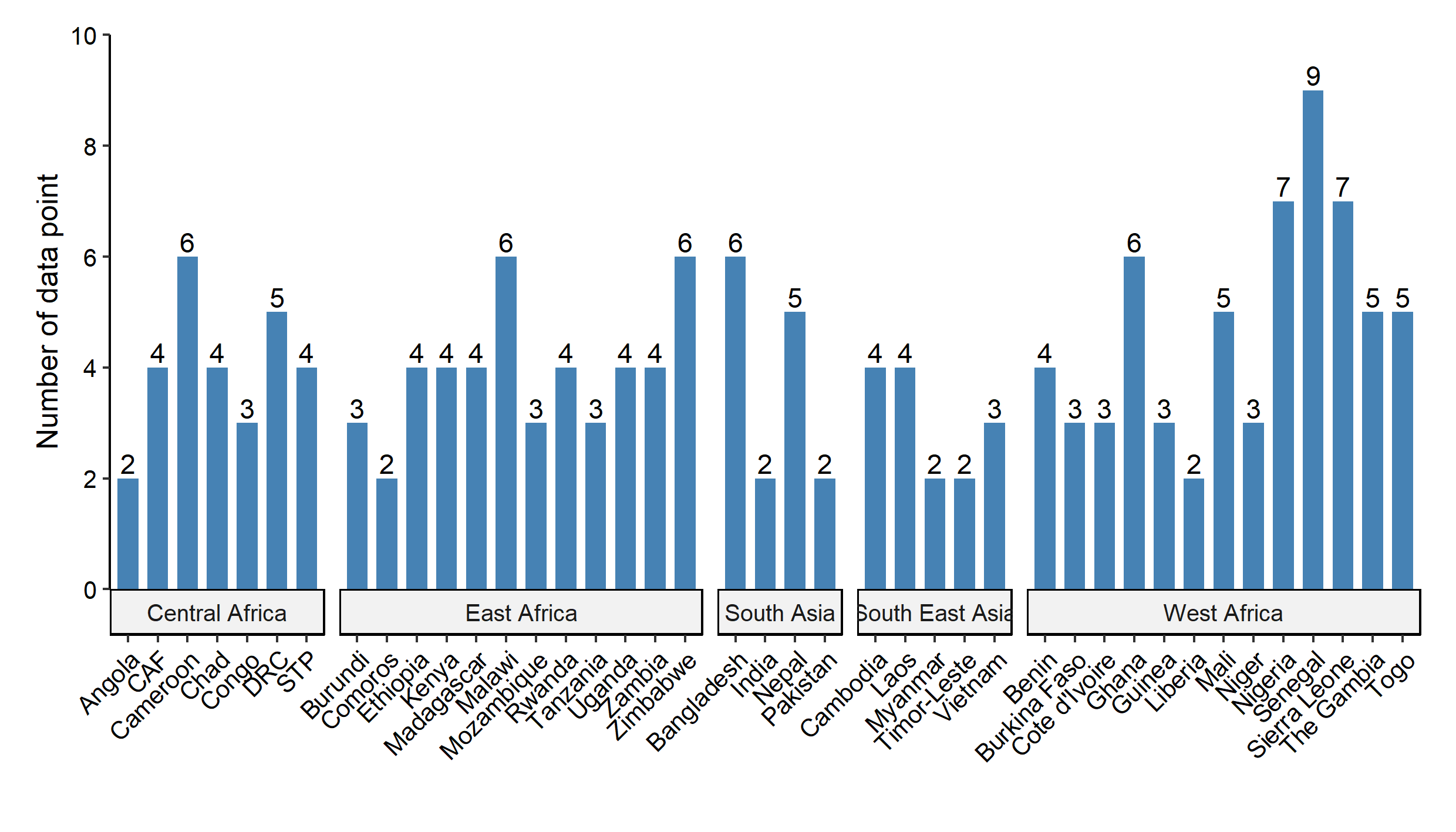


Supplementary Figure S1: Number of data points for this analysis

Supplementary Table S1: Percentage of vaccination card at national and by age group among the 174 included survey

| Country by region | Survey name | Year | Vaccination card seen (%) | | | | | |
| --- | --- | --- | --- | --- | --- | --- | --- | --- |
|  |  |  | **Total** | **0-11** | **12-23** | **24-35** | **36-47** | **48-59** |
| South Asia |  |  |  |  |  |  |  |  |
| Bangladesh | DHS | 2000 | 31.5 | 41.5 | 45.6 | 28.5 | 21.3 | 19.4 |
|  | DHS | 2004 | 38.3 | 57.3 | 52.2 | 34.5 | 25.5 | 22.8 |
|  | DHS | 2007 | 48.4 | 64.4 | 58.9 | 50.6 | 37.8 | 30.7 |
|  | DHS | 2011 | 57.9 | 65.2 | 67.9 | 57.2 | 52.8 | 48.0 |
|  | DHS | 2014 | 63.2 | 68.0 | 74.6 | 66.1 | 37.2 | 49.8 |
|  | DHS | 2017-18 | 68.1 | 63.3 | 74.4 | 67.8 |  |  |
| India | DHS | 2005-06 | 33.1 | 46.9 | 42.1 | 31.8 | 24.6 | 20.8 |
|  | DHS | 2015-16 | 53.1 | 66.2 | 62.6 | 53.9 | 44.9 | 38.6 |
| Nepal | DHS | 2001 | 16.0 | 40.3 | 16.6 | 10.4 | 7.6 | 5.8 |
|  | DHS | 2006 | 23.4 | 60.1 | 28.7 | 16.0 | 9.9 | 6.3 |
|  | DHS | 2011 | 28.0 | 63.9 | 34.8 | 20.1 | 13.6 | 8.9 |
|  | DHS | 2016 | 52.4 | 71.9 | 53.1 | 32.0 |  |  |
|  | MICS | 2019 | 65.6 | 77.3 | 70.8 | 50.2 |  |  |
| Pakistan | DHS | 2012-13 | 25.0 | 45.2 | 33.9 | 21.3 | 14.4 | 12.2 |
|  | DHS | 2017-18 | 53.8 | 61.0 | 58.6 | 41.8 |  |  |
| Southeast Asia |  |  |  |  |  |  |  |  |
| Cambodia | DHS | 2000 | 31.8 | 42.9 | 43.6 | 29.9 | 25.1 | 19.1 |
|  | DHS | 2005 | 51.3 | 70.4 | 62.5 | 49.7 | 38.2 | 33.4 |
|  | DHS | 2010 | 64.9 | 77.1 | 73.2 | 64.6 | 56.8 | 51.9 |
|  | DHS | 2014 | 68.5 | 80.6 | 75.0 | 68.8 | 61.6 | 55.1 |
| Laos | MICS | 2000 | 36.5 | 34.7 | 43.4 | 37.9 | 34.6 | 32.2 |
|  | MICS | 2006 | 36.3 | 45.4 | 49.2 | 37.8 | 26.2 | 22.2 |
|  | MICS | 2012 | 38.3 | 48.9 | 47.8 | 38.7 | 29.8 | 26.6 |
|  | MICS | 2017 | 65.6 | 72.5 | 66.1 | 58.5 |  |  |
| Myanmar | MICS | 2010 | 38.1 | 39.6 | 46.1 | 40.3 | 33.1 | 31.3 |
|  | DHS | 2015-16 | 32.4 | 38.8 | 42.5 | 34.6 | 25.3 | 20.7 |
| Timor-Leste | DHS | 2009-10 | 38.7 | 58.6 | 51.3 | 36.8 | 28.6 | 17.2 |
|  | DHS | 2016 | 49.6 | 57.3 | 50.8 | 39.8 |  |  |
| Vietnam | MICS | 2006 | 35.3 | 62.3 | 41.9 | 29.9 | 22.6 | 21.7 |
|  | MICS | 2011 | 40.5 | 65.3 | 51.4 | 37.6 | 28.1 | 21.7 |
|  | MICS | 2014 | 69.7 | 73.1 | 75.5 | 58.6 |  |  |
| Central Africa |  |  |  |  |  |  |  |  |
| Angola | MICS | 2000 | 29.2 | 44.4 | 33.4 | 23.8 | 22.4 | 19.3 |
|  | DHS | 2015-16 | 44.5 | 53.8 | 45.4 | 32.9 |  |  |
| Cameroon | MICS | 2000 | 41.9 | 55.5 | 50.4 | 39.3 | 33.1 | 28.7 |
|  | DHS | 2004 | 47.9 | 60.8 | 57.5 | 47.7 | 36.3 | 34.2 |
|  | MICS | 2006 | 49.4 | 66.6 | 65.8 | 46.3 | 33.8 | 29.2 |
|  | DHS | 2011 | 50.4 | 66.0 | 58.1 | 46.1 | 40.1 | 37.5 |
|  | MICS | 2014 | 66.0 | 71.8 | 69.5 | 56.4 |  |  |
|  | DHS | 2018 | 69.0 | 75.2 | 71.1 | 60.1 |  |  |
| CAF | MICS | 2000 | 32.6 | 42.6 | 39.8 | 30.5 | 24.0 | 21.9 |
|  | MICS | 2006 | 26.3 | 39.9 | 34.4 | 24.2 | 15.0 | 14.7 |
|  | MICS | 2010 | 23.6 | 36.9 | 29.9 | 18.8 | 15.5 | 13.1 |
|  | MICS | 2018-19 | 32.7 | 44.1 | 32.1 | 21.6 |  |  |
| Chad | MICS | 2000 | 19.1 | 28.8 | 27.6 | 18.3 | 11.5 | 9.6 |
|  | DHS | 2004 | 19.7 | 28.0 | 28.4 | 19.9 | 12.5 | 9.8 |
|  | MICS | 2010 | 17.5 | 26.3 | 21.9 | 15.9 | 13.4 | 10.6 |
|  | DHS | 2014-15 | 18.6 | 27.7 | 27.9 | 17.2 | 11.6 | 10.4 |
| Congo | DHS | 2005 | 55.8 | 70.4 | 62.9 | 51.9 | 45.1 | 44.6 |
|  | DHS | 2011-12 | 43.8 | 65.1 | 51.7 | 40.7 | 30.4 | 26.2 |
|  | MICS | 2014-15 | 42.4 | 58.2 | 40.4 | 27.7 |  |  |
| DRC | MICS | 2000 | 23.1 | 9.1 | 23.6 | 12.5 |  |  |
|  | DHS | 2007 | 19.5 | 31.3 | 24.1 | 17.8 | 12.4 | 9.2 |
|  | MICS | 2010 | 33.3 | 45.7 | 41.4 | 30.1 | 25.5 | 18.7 |
|  | DHS | 2013-14 | 17.5 | 24.8 | 22.3 | 15.8 | 13.1 | 10.1 |
|  | MICS | 2017-18 | 23.1 | 25.9 | 23.4 | 19.9 |  |  |
| Sao TP | MICS | 2000 | 76.8 | 88.7 | 84.2 | 77.3 | 70.1 | 60.9 |
|  | DHS | 2008-09 | 88.6 | 90.9 | 92.8 | 86.8 | 90.1 | 81.0 |
|  | MICS | 2014 | 90.7 | 92.8 | 91.7 | 87.8 |  |  |
|  | MICS | 2019 | 89.3 | 91.8 | 90.8 | 85.9 |  |  |
| East Africa |  |  |  |  |  |  |  |  |
| Burundi | MICS | 2005 | 44.3 | 71.2 | 61.5 | 37.5 | 25.7 | 21.0 |
|  | DHS | 2010 | 53.8 | 79.1 | 60.4 | 51.2 | 42.4 | 34.3 |
|  | DHS | 2016-17 | 78.9 | 92.1 | 82.5 | 60.3 |  |  |
| Comoros | MICS | 2000 | 60.6 | 73.3 | 71.4 | 61.1 | 53.8 | 43.3 |
|  | DHS | 2012 | 58.4 | 74.2 | 71.4 | 55.0 | 46.2 | 42.0 |
| Ethiopia | DHS | 2000 | 22.0 | 26.7 | 29.8 | 22.5 | 16.5 | 14.9 |
|  | DHS | 2005 | 24.4 | 34.0 | 35.7 | 23.5 | 15.9 | 13.1 |
|  | DHS | 2011 | 23.6 | 31.8 | 32.6 | 22.7 | 16.8 | 15.3 |
|  | DHS | 2016 | 34.2 | 41.5 | 37.5 | 22.8 |  |  |
|  | DHS | 2019 | 39.7 | 47.2 | 42.2 | 29.9 |  |  |
| Kenya | MICS | 2000 | 56.8 | 68.7 | 66.6 | 54.8 | 50.2 | 42.5 |
|  | DHS | 2003 | 50.5 | 62.4 | 58.1 | 48.3 | 41.7 | 39.4 |
|  | DHS | 2008-09 | 61.9 | 75.3 | 67.8 | 64.1 | 52.0 | 48.5 |
|  | DHS | 2014 | 68.1 | 83.6 | 75.3 | 66.6 | 60.1 | 55.1 |
| Madagascar | MICS | 2000 | 40.8 | 44.8 | 49.4 | 36.7 | 33.9 | 31.9 |
|  | DHS | 2003-04 | 45.1 | 58.2 | 51.6 | 43.5 | 37.3 | 34.0 |
|  | DHS | 2008-09 | 49.1 | 53.0 | 58.5 | 50.5 | 44.4 | 39.7 |
|  | MICS | 2018 | 50.6 | 54.1 | 52.1 | 45.4 |  |  |
| Malawi | DHS | 2000 | 69.2 | 78.0 | 81.0 | 72.0 | 58.8 | 49.2 |
|  | DHS | 2004 | 64.5 | 70.7 | 75.2 | 68.8 | 58.2 | 45.5 |
|  | MICS | 2006 | 67.0 | 76.2 | 77.0 | 64.9 | 56.6 | 55.3 |
|  | DHS | 2010 | 73.1 | 85.1 | 83.8 | 71.9 | 65.1 | 58.0 |
|  | MICS | 2013-14 | 77.9 | 89.4 | 82.6 | 62.2 |  |  |
|  | DHS | 2015-16 | 78.0 | 89.5 | 78.6 | 65.5 |  |  |
| Mozambique | DHS | 2003 | 66.7 | 76.5 | 80.1 | 68.3 | 55.2 | 50.7 |
|  | MICS | 2008 | 77.0 | 85.1 | 85.9 | 78.2 | 69.6 | 62.6 |
|  | DHS | 2011 | 75.2 | 84.2 | 83.6 | 75.3 | 68.0 | 61.3 |
| Rwanda | DHS | 2000 | 54.6 | 68.8 | 65.9 | 54.3 | 40.9 | 39.6 |
|  | DHS | 2005 | 69.7 | 82.5 | 76.6 | 69.4 | 59.5 | 55.7 |
|  | DHS | 2010 | 75.7 | 92.6 | 82.4 | 76.3 | 68.6 | 61.4 |
|  | DHS | 2014-15 | 90.0 | 95.0 | 94.1 | 89.5 | 87.1 | 82.7 |
| Tanzania | DHS | 2004-05 | 71.3 | 79.4 | 80.9 | 72.0 | 63.4 | 58.0 |
|  | DHS | 2010 | 75.4 | 81.0 | 83.9 | 74.8 | 71.5 | 64.7 |
|  | DHS | 2015-16 | 79.6 | 81.5 | 84.3 | 72.0 |  |  |
| Uganda | DHS | 2000-01 | 40.0 | 47.0 | 47.2 | 38.7 | 34.3 | 30.3 |
|  | DHS | 2006 | 52.2 | 64.0 | 62.6 | 54.5 | 42.9 | 35.1 |
|  | DHS | 2011 | 51.7 | 68.1 | 59.0 | 47.3 | 42.1 | 40.4 |
|  | DHS | 2016 | 70.5 | 81.3 | 70.0 | 59.5 |  |  |
| Zambia | DHS | 2001-02 | 68.2 | 75.1 | 79.8 | 71.4 | 58.3 | 52.7 |
|  | DHS | 2007 | 68.3 | 74.8 | 77.6 | 70.8 | 60.9 | 54.6 |
|  | DHS | 2014 | 69.9 | 78.1 | 80.6 | 70.9 | 63.0 | 57.3 |
|  | DHS | 2018 | 74.9 | 79.8 | 76.6 | 68.0 |  |  |
| Zimbabwe | DHS | 2005-06 | 66.3 | 73.0 | 72.3 | 62.8 | 61.2 | 60.8 |
|  | MICS | 2009 | 67.4 | 73.9 | 74.9 | 69.1 | 63.1 | 56.6 |
|  | DHS | 2010-11 | 61.7 | 68.4 | 68.3 | 60.4 | 55.5 | 51.5 |
|  | MICS | 2014 | 74.5 | 86.6 | 80.8 | 73.6 | 66.5 | 65.2 |
|  | DHS | 2015 | 78.4 | 85.2 | 78.6 | 71.0 |  |  |
|  | MICS | 2019 | 84.1 | 85.5 | 85.5 | 81.5 |  |  |
| West Africa |  |  |  |  |  |  |  |  |
| Benin | DHS | 2001 | 60.6 | 76.1 | 73.2 | 62.4 | 48.7 | 38.7 |
|  | DHS | 2006 | 53.9 | 67.5 | 65.9 | 53.6 | 41.3 | 37.2 |
|  | DHS | 2011-12 | 45.4 | 64.0 | 54.4 | 43.7 | 34.6 | 29.9 |
|  | MICS | 2014 | 70.4 | 80.7 | 72.3 | 57.6 |  |  |
|  | DHS | 2017-18 | 72.3 | 80.8 | 70.7 | 63.3 |  |  |
| Burkina Faso | DHS | 2003 | 53.1 | 56.6 | 69.4 | 58.3 | 43.6 | 37.1 |
|  | MICS | 2006 | 63.0 | 73.1 | 77.5 | 64.1 | 49.9 | 43.7 |
|  | DHS | 2010 | 70.0 | 85.1 | 83.5 | 73.3 | 57.4 | 47.9 |
| Cote d'Ivoire | MICS | 2000 | 69.3 | 75.5 | 76.6 | 69.8 | 62.3 | 58.7 |
|  | MICS | 2006 | 64.2 | 68.2 | 72.6 | 64.1 | 58.3 | 55.2 |
|  | DHS | 2011-12 | 67.5 | 77.8 | 75.6 | 67.8 | 59.2 | 53.9 |
|  | MICS | 2016 | 77.6 | 85.7 | 78.9 | 67.1 |  |  |
| The Gambia | MICS | 2000 | 80.2 | 86.3 | 89.8 | 81.0 | 71.8 | 67.0 |
|  | MICS | 2005-06 | 83.5 | 87.2 | 90.3 | 86.3 | 76.8 | 70.1 |
|  | MICS | 2010 | 87.4 | 91.7 | 94.1 | 86.3 | 82.3 | 79.6 |
|  | DHS | 2013 | 78.7 | 87.6 | 90.4 | 79.5 | 68.8 | 62.2 |
|  | MICS | 2018 | 90.5 | 90.2 | 94.5 | 87.0 |  |  |
|  | DHS | 2019-20 | 90.7 | 91.5 | 93.9 | 86.3 |  |  |
| Ghana | DHS | 2003 | 70.7 | 72.7 | 81.5 | 74.4 | 65.1 | 58.3 |
|  | MICS | 2006 | 73.8 | 83.4 | 86.1 | 78.4 | 61.3 | 58.5 |
|  | DHS | 2008 | 74.4 | 79.4 | 85.9 | 79.9 | 65.5 | 60.1 |
|  | MICS | 2011 | 81.6 | 88.3 | 89.6 | 84.1 | 75.6 | 70.7 |
|  | DHS | 2014 | 79.4 | 86.2 | 89.0 | 81.6 | 70.8 | 67.5 |
|  | MICS | 2017-18 | 68.4 | 70.8 | 68.3 | 66.2 |  |  |
| Guinea | DHS | 2005 | 42.0 | 51.4 | 53.6 | 43.2 | 31.6 | 26.3 |
|  | DHS | 2012 | 37.0 | 49.2 | 45.3 | 39.7 | 28.2 | 20.1 |
|  | MICS | 2016 | 38.9 | 55.0 | 49.5 | 37.7 | 32.9 | 24.8 |
|  | DHS | 2018 | 63.8 | 73.4 | 62.2 | 53.6 |  |  |
| Liberia | DHS | 2007 | 30.4 | 52.1 | 44.3 | 28.8 | 14.6 | 10.0 |
|  | DHS | 2013 | 45.0 | 70.5 | 58.6 | 38.4 | 29.6 | 22.9 |
|  | DHS | 2019-20 | 64.8 | 73.7 | 65.8 | 53.1 |  |  |
| Mali | DHS | 2001 | 36.9 | 41.5 | 47.5 | 37.1 | 29.5 | 26.5 |
|  | DHS | 2006 | 46.6 | 59.0 | 60.1 | 46.5 | 34.4 | 29.4 |
|  | MICS | 2009-10 | 46.4 | 59.9 | 56.9 | 43.2 | 34.2 | 30.5 |
|  | DHS | 2012-13 | 29.3 | 41.9 | 38.8 | 26.7 | 19.9 | 18.8 |
|  | MICS | 2015 | 41.0 | 50.6 | 42.3 | 29.4 |  |  |
|  | DHS | 2018 | 52.7 | 65.0 | 51.6 | 40.0 |  |  |
| Niger | MICS | 2000 | 43.3 | 50.4 | 48.9 | 45.3 | 36.8 | 34.6 |
|  | DHS | 2006 | 39.7 | 47.6 | 50.4 | 40.5 | 30.1 | 27.0 |
|  | DHS | 2012 | 48.0 | 62.1 | 65.6 | 46.5 | 33.7 | 31.2 |
| Nigeria | DHS | 2003 | 17.9 | 25.4 | 23.4 | 17.3 | 11.0 | 9.6 |
|  | MICS | 2007 | 13.3 | 22.6 | 17.6 | 11.0 | 8.0 | 6.8 |
|  | DHS | 2008 | 20.2 | 29.1 | 25.5 | 18.8 | 13.5 | 12.1 |
|  | MICS | 2011 | 21.5 | 33.7 | 28.1 | 19.8 | 14.8 | 10.5 |
|  | DHS | 2013 | 25.1 | 35.7 | 30.2 | 23.6 | 19.1 | 15.2 |
|  | MICS | 2016-17 | 49.0 | 66.6 | 48.4 | 33.3 |  |  |
|  | DHS | 2018 | 40.3 | 50.2 | 40.1 | 29.7 |  |  |
| Senegal | DHS | 2005 | 56.0 | 70.5 | 70.4 | 55.0 | 43.3 | 33.7 |
|  | DHS | 2010-11 | 53.9 | 73.0 | 65.7 | 51.2 | 41.5 | 34.4 |
|  | cDHS | 2012-13 | 58.4 | 80.1 | 69.1 | 56.4 | 44.7 | 39.3 |
|  | cDHS | 2014 | 56.6 | 77.3 | 69.6 | 54.7 | 44.0 | 37.0 |
|  | cDHS | 2015 | 60.5 | 80.6 | 73.1 | 57.2 | 49.1 | 40.2 |
|  | cDHS | 2016 | 57.3 | 77.5 | 71.7 | 54.8 | 43.6 | 38.2 |
|  | cDHS | 2017 | 74.7 | 86.3 | 77.7 | 59.2 |  |  |
|  | cDHS | 2018 | 76.5 | 87.0 | 80.2 | 60.6 |  |  |
|  | cDHS | 2019 | 79.9 | 89.6 | 85.0 | 64.6 |  |  |
| Sierra Leone | MICS | 2000 | 29.6 | 44.5 | 39.0 | 29.9 | 20.7 | 17.0 |
|  | MICS | 2005-06 | 38.8 | 59.5 | 53.7 | 38.1 | 25.6 | 17.3 |
|  | DHS | 2008 | 47.6 | 62.6 | 60.0 | 46.9 | 31.8 | 28.8 |
|  | MICS | 2010 | 49.6 | 74.5 | 67.3 | 48.0 | 36.3 | 25.3 |
|  | DHS | 2013 | 62.5 | 78.6 | 74.3 | 62.5 | 53.2 | 42.0 |
|  | MICS | 2017 | 66.1 | 74.5 | 75.9 | 66.5 | 58.7 | 55.5 |
|  | DHS | 2019 | 74.8 | 84.2 | 76.1 | 62.7 |  |  |
| Togo | MICS | 2000 | 57.5 | 65.1 | 65.8 | 60.0 | 45.8 | 47.1 |
|  | MICS | 2006 | 59.5 | 70.4 | 70.1 | 60.3 | 45.8 | 44.8 |
|  | MICS | 2010 | 49.6 | 74.5 | 67.3 | 48.0 | 36.3 | 25.3 |
|  | DHS | 2014 | 59.8 | 75.6 | 72.1 | 57.6 | 49.8 | 40.9 |
|  | MICS | 2017 | 70.0 | 75.1 | 70.5 | 64.4 |  |  |

Note: CAF, Central African Republic; DRC, Democratic Republic of the Congo; STP, Sao Tome and Principe; cDHS, Continuous Demographic and Health Survey; DHS, Demographic and Health Survey; MICS, Multiple Indicator Cluster Survey

Supplementary Table S2: The recommended age for vaccination according to national immunization schedules by country*

| **Countries by regions** | **Target age (weeks)** | | | |
| --- | --- | --- | --- | --- |
|  | **BCG** | **DTP3** | **polio3** | **MCV** |
| **South Asia** |  |  |  |  |
| Bangladesh | 0 | 14 | 14 | 39 |
| India | 0 | 14 | 14 | 39 |
| Nepal | 0 | 14 | 14 | 39 |
| Pakistan | 0 | 14 | 14 | 39 |
| **Southeast Asia** |  |  |  |  |
| Cambodia | 0 | 14 | 14 | 39 |
| Laos | 0 | 14 | 14 | 39 |
| Myanmar | 0-2 months | 6 months | 6 months | 39 |
| Timor-Leste | 0 | 14 | 14 | 39 |
| Vietnam | 0 | 4 months | 4 months | 39 |
| **Central Africa** |  |  |  |  |
| Angola | 0 | 6 months | 6 months | 39 |
| Cameroon | 0 | 14 | 14 | 39 |
| CAF | 0 | 14 | 14 | 39 |
| Chad | 0 | 14 | 14 | 39 |
| Congo | 0 | 16 | 16 | 39 |
| DRC | 0 | 14 | 14 | 39 |
| Sao TP | 0 | 14 | 14 | 39 |
| **East Africa** |  |  |  |  |
| Burundi | 0 | 14 | 14 | 39 |
| Comoros | 0 | 14 | 14 | 39 |
| Ethiopia | 0 | 14 | 14 | 39 |
| Kenya | 0 | 14 | 14 | 39 |
| Madagascar | 0 | 14 | 14 | 39 |
| Malawi | 0 | 14 | 14 | 39 |
| Mozambique | 0 | 14 | 14 | 39 |
| Rwanda | 0 | 14 | 14 | 39 |
| Tanzania | 0 | 14 | 14 | 39 |
| Uganda | 0 | 14 | 14 | 39 |
| Zambia | 1 | 14 | 14 | 39 |
| Zimbabwe | 0 | 14 | 14 | 39 |
| **West Africa** |  |  |  |  |
| Benin | 0 | 14 | 14 | 39 |
| Burkina Faso | 0 | 16 | 16 | 39 |
| Cote d'Ivoire | 0 | 14 | 14 | 39 |
| The Gambia | 0 | 4 months | 4 months | 39 |
| Ghana | 0 | 14 | 14 | 39 |
| Guinea | 0 | 14 | 14 | 39 |
| Liberia | 0 | 14 | 14 | 39 |
| Mali | 0 | 14 | 14 | 39 |
| Niger | 0 | 14 | 14 | 39 |
| Nigeria | 0 | 14 | 14 | 39 |
| Senegal | 0 | 14 | 14 | 39 |
| Sierra Leone | 0 | 14 | 14 | 39 |
| Togo | 0 | 14 | 14 | 39 |

*Information on vaccination was based on the WHO report on vaccine-preventable diseases: monitoring system 2020 global summary.

Note: CAF, Central African Republic; DRC, Democratic Republic of the Congo; Sao TP, Sao Tome and Principe

Supplementary method S1: Bayesian model

The Bayesian approach was used and favoured with the aim to project probabilities, which would not be possible using a frequentist approach. The essential difference between the two approaches is how probability is used. If we were to use a frequentist approach, the 95% CIs cannot calculate the probability of observing a future value, and hence we would be limited to using probability to *only* model certain processes (using process of “sampling”). For the purpose of our study, we were required to use probability more widely to model both sampling and other kinds of uncertainty which could only be conducted using a Bayesian approach.

As a result of the advantage of producing probabilistic-oriented inferences, Bayesian methods are increasingly being applied to scientific fields, particularly in ecology where many cases outperform deterministic approaches. Since ecological modelling is characterized by high uncertainty due to the complex and often unknown cause-effect relationships among variables, a probabilistic approach is necessary to yield distributions of possible outcomes - in essence, transforming uncertainty into probability thresholds. This unique advantage of Bayesian approach over frequentist approach was key to conducting our study. Another advantage of using Bayesian methods in our study was the ability to combine prior knowledge about parameters with evidence from data. This method is favored for analysis of hierarchical models such as our study, which enables flexibility in specifying hierarchical structures of parameters using priors; ability to handle small samples and model misspecification (overparameterization of the likelihood can be resolved with well-chosen priors); explicit handling of uncertainty; and intuitive and easy interpretation of results (credible interval versus confidence interval).

The hierarchical Bayesian modelling approach was implemented to obtain trend and projection values from 2000 to 2030. The proportion (coverage) of all outcome variables was transformed into logit scales before modeling. We fitted the models using the Bayesian approach, sampling from the posterior distribution of the parameters using Gibbs Monte Carlo, a Markov chain Monte Carlo (MCMC) method, as implemented in the algorithm in the JAGS open-source software (version 4.2). In the MCMC algorithm, 20, 000 iterations with two chains, 20 thinning, and 500 burn-in samples were used. Noninformative priors were used in this study. Trace plots and Gelman-Rubin diagnostic statistics were used to check the convergence status. Statistical analysis was performed using R 3.2.0. The 95% credible intervals (CrI) were constructed from the 2.5th and 97.5th percentiles of the posterior samples. The basic model equation with notation are as follows:

$$y_{ijk}\sim N(X\beta_{ijk},\tau^{2})$$

$$y_{ijk}=\beta_{0,ij}+\beta_{1,ij}{year}_{i}+\beta_{2,ik}{SDI}_{ij}+\beta_{3,ik}{HW}_{ij}+\beta_{4,ik}{GSH}_{ij}+\beta_{5,ik}{DAH}_{ij}+\varepsilon_{ijk}$$

$$\beta_{ij}\sim N(\beta_{i},\sigma_{i}^{2})$$

$$\beta_{i}\sim N(\beta,\sigma^{2})$$

$$\beta\sim N(0,10000)$$

$$\sigma_{i},\sigma,\tau\sim gamma(0.0001,0.001)$$

where $y_{ijk}$ is the logit-transformed probability of outcomes in the ith year for the jth country in the kth region. X is the model matrix consisting of observed sociodemographic index (SDI), skilled health workforce (SHW), Government spending on health (GSH), and Development assistance for health (DAH) values in the kth year for jth country, $\beta_{ij}=[\beta_{0,ij},\beta_{1,ij},\beta_{2,ij},\beta_{3,ij},\beta_{4,ij},\beta_{5,ij}]$ is the year and country-specific linear regression model parameter vector which includes the intercept $\beta_{0,ij}$ and the slops for year ($\beta_{1,ij}$), SDI ($\beta_{2,ij}$) , SHW ($\beta_{3,ij}$), GSH ($\beta_{4,ij}$), and DAH ($\beta_{5,ij}$), $\tau^{2}$ is the model error variance, $\beta_{j}=[\beta_{0,j},\beta_{1,j},\beta_{2,j},\beta_{3,j},\beta_{4,j},\beta_{5,j}]$ is the vector of model parameter means for country in region k, $\sigma_{j}^{2}={[\sigma}_{0,j}^{2}, \sigma_{1,j}^{2}, \sigma_{2,j}^{2}, \sigma_{3,j}^{2}, \sigma_{4,j}^{2}, \sigma_{5,j}^{2}]$ is the vector representing the variance of model parameters among countries belonging to region k, while $\beta=[\beta_{0}, \beta_{1}, \beta_{2}, \beta_{3}, \beta_{4}, \beta_{5}]$ and $\sigma^{2}=[\sigma_{0}^{2}, \sigma_{1}^{2}, \sigma_{2}^{2}, \sigma_{3}^{2}, \sigma_{4}^{2}, \sigma_{5}^{2}]$ are the means and variance among region, respectively. Non-informative prior distributions were assigned to $\beta,\tau,\sigma_{j}, and \sigma$ which represent the hyperparameters follow a normal distribution with mean 0 and variance 10,000; a uniform distribution with lower (0) and upper (1000) limits; and a gamma distribution with shape parameter k (0.001) and scale parameter ($\theta)$(0.0001), respectively. The hyperparameters of $\tau,\sigma_{j}, and \sigma$ $\tau, \sigma_{i}, and \sigma$are considered non-informative as there is no information about their distribution.

Supplementary method S2: sensitivity analysis

Both covariates and the model’s hierarchical structure influence how data from other countries influence predictions for a given country.^1^ We examined the sensitivity of our results by two approaches: (1) the exclusion of country-level covariates (SDI and SHW), and (2) altering priors for the hyperparameters.

*Sensitivity analysis: assessing the role of country-level covariates:*

Excluding country level predictors, the median absolute differences between the two sets of results were compared.

*Altering priors for the hyperparameters:*

Previous studies reported that to borrow strength and facilitate parameters smoothening from each group, the hyperparameters were shared by all intercept coefficients.^2,3^ The key benefit of assigning the hyperparameter is the fact that the resulting model gains the advantages of a complete-pooled model and a no-pooled model. The half-Cauchy is quite heavy tailed and considered fairly weakly informative. Gelman (2006) advocates for half-t priors (including the half-Cauchy) over the inverse gamma. The hyperparameter priors (hyper-priors) were assigned flat non-informative prior distributions for main analysis and weekly informative hyper-priors for sensitivity analysis. In our proposed model, $\beta_{jk}$ are conditionally normally distributed on $\beta_{k}, \sigma_{k}^{2}$ while $\beta_{k}$ is conditionally normally distributed on $\beta and \sigma^{2}.$ In the main analysis, $\beta=[{\beta_{0},\beta}_{1}, \beta_{2}, \beta_{3}, \beta_{4}, \beta_{5}]$ is the normal distribution was specified in terms of mean zero and the standard deviation of 100, $[{\beta_{0},\beta}_{1}, \beta_{2}, \beta_{3}, \beta_{4}, \beta_{5}]\sim$N(0.0.0001). Since the hyperparameter have some influences on all intercept coefficients, the half-Cauchy distribution (weekly informative prior) applied to perform sensitivity analysis instead of gamma distribution, hyperparameters (𝜏, 𝜎_k_, 𝑎𝑛𝑑 𝜎) ~ half-Cauchy (0,25). After altering prior distribution, the median absolute differences between the two sets of results were also compared.

***References:***

1. Danaei G, Finucane MM, Lin JK, et al. National, regional, and global trends in systolic blood pressure since 1980: systematic analysis of health examination surveys and epidemiological studies with 786 country-years and 5· 4 million participants. *The Lancet* 2011; **377**(9765): 568-77.
2. Ntzoufras I. Bayesian modeling using WinBUGS: John Wiley & Sons; 2011.
3. Gelman A. Prior distributions for variance parameters in hierarchical models (comment on article by Browne and Draper). *Bayesian analysis* 2006; **1**(3): 515-34.

Supplementary Table S3: Region-specific coverage of age-appropriate vaccines between 2000 and 2030

| **Vaccine**  **name** | **Region** | **Predicted coverage (95% credible intervals)** | | |  | **Rate of change^a^** | **Required rate of change^b^** |
| --- | --- | --- | --- | --- | --- | --- | --- |
|  |  | **2000** | **2020** | **2030** |  |  |  |
| **BCG** | South Asia | 17.1 (16.5-17.8) | 55.1 (54.3-55.8) | 74.9 (74.0-75.8) |  | 6.0 | 3.0 |
|  | Southeast Asia | 26.2 (25.3-27.1) | 70.0 (69.4-70.6) | 85.7 (85.1-86.2) |  | 5.0 | 5.0 |
|  | Central Africa | 40.1 (38.8-41.1) | 55.5 (54.9-56.2) | 63.1 (62.0-64.2) |  | 1.6 | 2.5 |
|  | East Africa | 42.5 (41.3-43.7) | 74.6 (74.0-75.1) | 85.4 (84.8-86.0) |  | 2.9 | 5.0 |
|  | West Africa | 46.3 (45.0-47.4) | 69.8 (69.2-70.3) | 79.1 (78.3-79.9) |  | 2.1 | 1.9 |
|  | Overall | 39.0 (37.9-40.1) | 67.1 (66.5-67.7) | 78.4 (77.7-79.2) |  | 2.8 | 2.6 |
| **DTP3** | South Asia | 19.2 (18.5-19.9) | 56.3 (55.7-57.0) | 75.0 (74.1-75.9) |  | 4.8 | 8.0 |
|  | Southeast Asia | 16.1 (15.6-16.7) | 41.5 (40.9-42.1) | 57.7 (56.7-58.8) |  | 6.2 | 8.4 |
|  | Central Africa | 14.8 (14.2-15.3) | 34.3 (33.7-34.9) | 47.6 (46.4-48.7) |  | 4.3 | 10.1 |
|  | East Africa | 12.1 (11.6-12.6) | 40.3 (39.6-40.9) | 59.8 (58.8-60.9) |  | 5.5 | 4.8 |
|  | West Africa | 14.4 (13.8-15.0) | 38.2 (37.6-38.7) | 54.2 (53.0-55.2) |  | 5.0 | 8.9 |
|  | Overall | 16.3 (15.7-16.9) | 43.0 (42.4-43.7) | 59.8 (58.8-60.8) |  | 5.0 | 7.7 |
| **polio3** | South Asia | 21.1 (20.7-21.5) | 43.4 (43.1-43.8) | 56.6 (56.0-57.1) |  | 3.7 | 7.6 |
|  | Southeast Asia | 13.3 (13.0-13.5) | 42.4 (42.1-42.8) | 61.8 (61.2-62.4) |  | 6.0 | 7.8 |
|  | Central Africa | 15.1 (14.8-15.4) | 40.1 (39.8-40.5) | 56.6 (56.0-57.2) |  | 5.0 | 8.4 |
|  | East Africa | 20.0 (19.6-20.4) | 54.9 (54.5-55.2) | 72.8 (72.3-73.3) |  | 5.2 | 5.1 |
|  | West Africa | 17.5 (17.1-17.8) | 39.2 (38.9-39.6) | 53.0 (52.4-53.6) |  | 4.1 | 8.7 |
|  | Overall | 17.9 (17.6-18.2) | 44.7 (44.3-45.0) | 60.9 (60.3-61.4) |  | 4.7 | 7.2 |
| **MCV** | South Asia | 30.7 (29.6-31.8) | 51.6 (50.8-52.3) | 62.3 (61.0-63.5) |  | 4.9 | 5.3 |
|  | Southeast Asia | 20.6 (19.8-21.5) | 53.9 (53.2-54.7) | 71.3 (70.2-72.4) |  | 3.5 | 6.1 |
|  | Central Africa | 17.5 (16.8-18.3) | 32.3 (31.6-33.0) | 41.7 (40.3-43.0) |  | 3.1 | 10.8 |
|  | East Africa | 24.8 (23.8-25.8) | 49.6 (48.8-50.3) | 62.9 (61.7-64.1) |  | 2.6 | 5.7 |
|  | West Africa | 25.0 (24.0-26.0) | 42.5 (41.8-43.2) | 52.4 (51.1-53.6) |  | 2.7 | 7.8 |
|  | Overall | 25.2 (24.2-26.1) | 45.2 (44.4-45.9) | 56.3 (55.0-57.6) |  | 3.0 | 7.1 |

Note: ^a^Rate of change during 2000-2020; ^b^Required rate of change during 2020-2030 to achieve 90% vaccination coverage by 2030. It was calculated only for countries that are predicted to have a coverage less than 90% in 2030

Supplementary Table S4: Rate of change in the national coverage of age-appropriate vaccinations

| **Country** | Annual rate of change (%) during 2000-2020 | | | |  | Expected annual rate of change (%) during 2020-2030 | | | |
| --- | --- | --- | --- | --- | --- | --- | --- | --- | --- |
|  | BCG | DTP3 | polio3 | MCV |  | BCG | DTP3 | polio3 | MCV |
| **South Asia** |  |  |  |  |  |  |  |  |  |
| Bangladesh | 5.5 | 3.4 | 3.3 | 1.8 |  | 3.7 | 2.3 | 2.2 | 1.3 |
| India | 5.6 | 2.2 | 0.7 | 3.2 |  | 2.1 | 2.2 | 1.2 | 2.5 |
| Nepal | 6.0 | 5.2 | 2.1 | 6.8 |  | 2.4 | 3.1 | 1.8 | 2.6 |
| Pakistan | 8.5 | 12.0 | 9.3 | 13.4 |  | 3.0 | 7.0 | 5.4 | 5.9 |
| **Southeast Asia** |  |  |  |  |  |  |  |  |  |
| Cambodia | 8.2 | 10.6 | 9.8 | 8.1 |  | 0.7 | 2.1 | 2.1 | 2.2 |
| Laos | 8.3 | 8.2 | 7.9 | 8.4 |  | 2.7 | 5.4 | 5.0 | 4.6 |
| Myanmar | 1.0 | 3.5 | 3.4 | 0.5 |  | 0.9 | 3.2 | 2.9 | 1.1 |
| Timor-Leste | 4.8 | 4.7 | 6.1 | 4.8 |  | 2.5 | 4.4 | 4.6 | 3.3 |
| Vietnam | 4.3 | -0.9 | 0.4 | -0.8 |  | 1.7 | 1.2 | 1.3 | -0.5 |
| **Central Africa** |  |  |  |  |  |  |  |  |  |
| Angola | 4.6 | 8.6 | 6.7 | 7.4 |  | 2.4 | 5.4 | 4.4 | 4.5 |
| Cameroon | 2.0 | 4.2 | 5.0 | 2.0 |  | 0.5 | 1.4 | 0.6 | -0.1 |
| CAR | 2.2 | 3.3 | 5.4 | 2.0 |  | 2.0 | 7.3 | 5.6 | 3.7 |
| Chad | 1.6 | 1.9 | 2.8 | 5.3 |  | 0.2 | 2.1 | 3.2 | 3.6 |
| Congo | 0.7 | 0.2 | 1.8 | 4.3 |  | 0.9 | 2.7 | 1.6 | 1.3 |
| DRC | 11.8 | 7.4 | 11.4 | 6.9 |  | -5 | -7.2 | -2.3 | -7.8 |
| Sao TP | 1.4 | 5.4 | 5.9 | 3.4 |  | 0.3 | 1.4 | 1.5 | 1.6 |
| **East Africa** |  |  |  |  |  |  |  |  |  |
| Burundi | 3.8 | 9.9 | 6.0 | 4.3 |  | 0.4 | 1.4 | 1.4 | 0.8 |
| Comoros | 2.3 | 6.0 | 6.2 | 2.8 |  | 1.0 | 3.3 | 3.4 | 2.1 |
| Ethiopia | 4.6 | 5.5 | 2.9 | 4.4 |  | 4.2 | 5.8 | 4.3 | 5.2 |
| Kenya | 2.0 | 2.5 | 3.3 | 1.5 |  | 1.0 | 1.5 | 1.5 | 1.0 |
| Madagascar | 3.1 | 2.7 | 3.6 | 1.3 |  | 2.0 | 2.0 | 2.3 | 0.7 |
| Malawi | 6.3 | 6.5 | 6 | 3.3 |  | 1.1 | 2.5 | 3.0 | 1.8 |
| Mozambique | 1.7 | 5.8 | 6.1 | 1.8 |  | 0.2 | 3.4 | 3.5 | 0.5 |
| Rwanda | 1.8 | 3.2 | 3.0 | 1.5 |  | 0.3 | 0.8 | 0.8 | 0.5 |
| Tanzania | 1.0 | 4.5 | 4.3 | 2.1 |  | 0.1 | 2.2 | 2.0 | 1.0 |
| Uganda | 5.4 | 6.0 | 4.8 | 4.1 |  | 1.2 | 3.0 | 2.7 | 2.1 |
| Zambia | 2.4 | 8.8 | 7.9 | 2.5 |  | 1.0 | 4.2 | 4.2 | 1.9 |
| Zimbabwe | 8.4 | 17.2 | 14.5 | 9.1 |  | 0.7 | 2.8 | 3.0 | 2.1 |
| **West Africa** |  |  |  |  |  |  |  |  |  |
| Benin | 0.3 | 1.3 | 0.8 | 2.7 |  | -0.6 | 1.9 | 1.3 | 1.0 |
| Burkina Faso | 3.9 | 8.3 | 8.0 | 7.2 |  | 0.6 | 3.0 | 3.1 | 1.5 |
| Cote d'Ivoire | 0.3 | 0.9 | 2.2 | 0.3 |  | 0.4 | 0.4 | 1.0 | -0.2 |
| The Gambia | 1.3 | 6.7 | 5.2 | 1.6 |  | 0.7 | 5.1 | 4.1 | 1.1 |
| Ghana | 2.8 | 6.2 | 4.5 | 3.0 |  | 1.1 | 2.7 | 2.4 | 1.6 |
| Guinea | 2.1 | 0.6 | 2.0 | -0.1 |  | 0.5 | 0.8 | 2.1 | 0.0 |
| Liberia | 5.7 | 8.0 | 6.1 | 6.2 |  | 1.5 | 4.5 | 3.6 | 3.3 |
| Mali | 2.8 | 4.1 | 2.6 | 3.2 |  | 0.6 | 1.4 | 0.9 | -0.5 |
| Niger | 0.9 | 1.1 | 1.7 | 0.6 |  | 1.3 | 2.9 | 2.4 | 1.7 |
| Nigeria | 8.9 | 10.9 | 8.7 | 8.7 |  | 5.1 | 7.9 | 6.6 | 6.8 |
| Senegal | 3.0 | 4.2 | 3.7 | 4.0 |  | 1.4 | 2.5 | 2.5 | 2.0 |
| Sierra Leone | 2.7 | 3.4 | 1.5 | 3.4 |  | 0.9 | 4.5 | 3.0 | 3.3 |
| Togo | 1.0 | 7.4 | 6.2 | 4.4 |  | 0.3 | 3.5 | 3.5 | 2.1 |

Note: CAF, Central African Republic; DRC, Democratic Republic of the Congo; Sao TP, Sao Tome and Principe

Supplementary Table S5: Coverage of age-appropriate BCG vaccine according to the place of residence in 41 LMICs in Asia and Sub-Saharan Africa, 2000-2030

| **Country** | **Predicted coverage (95% credible intervals) in year** | | | | | | | |
| --- | --- | --- | --- | --- | --- | --- | --- | --- |
|  | **2000** | |  | **2020** | |  | **2030** | |
|  | **Urban** | **Rural** |  | **Urban** | **Rural** |  | **Urban** | **Rural** |
| **South Asia** |  |  |  |  |  |  |  |  |
| Bangladesh | 11.1 (8.4-14.3) | 8.6 (6.5-11.1) |  | 36.6 (29.2-44.4) | 30.4 (23.7-37.1) |  | 55.1 (41.6-68.1) | 48.2 (34.8-61.0) |
| India | 32.5 (20.3-46.6) | 18.2 (10.9-28.2) |  | 78.4 (68.1-86.7) | 62.6 (51.2-73.9) |  | 90.5 (80.4-96.3) | 81.5 (66.5-91.9) |
| Nepal | 24.3 (18.5-31.4) | 15.9 (12.0-20.6) |  | 69.0 (61.7-76.4) | 56.8 (49.3-64.0) |  | 85.1 (77.7-91.0) | 77.2 (67.9-85.0) |
| Pakistan | 26.1 (8.3-50.1) | 12.0 (3.5-25.8) |  | 74.3 (64.5-83.2) | 52.0 (40.5-63.4) |  | 88.6 (75.6-96.6) | 75.0 (53.8-91.3) |
| **Southeast Asia** |  |  |  |  |  |  |  |  |
| Cambodia | 31.8 (23.8-39.9) | 17.2 (13.0-22.2) |  | 95.6 (92.8-97.3) | 90.6 (85.8-94.0) |  | 99.3 (98.5-99.7) | 98.4 (96.8-99.3) |
| Laos | 30.5 (22.9-38.0) | 11.7 (8.5-15.1) |  | 84.4 (78.7-89.5) | 62.3 (53.9-70.4) |  | 94.9 (91.2-97.4) | 85.0 (76.1-91.4) |
| Myanmar | 59.2 (48.2-69.0) | 39.3 (30.3-49.3) |  | 70.5 (59.6-80.2) | 51.8 (40.5-63.1) |  | 74.9 (60.2-86.8) | 57.6 (40.3-74.0) |
| Timor-Leste | 23.1 (10.0-40.9) | 12.4 (5.0-24.1) |  | 73.6 (60.4-84.2) | 56.7 (43.8-69.3) |  | 88.5 (72.3-96.8) | 78.7 (56.5-93.1) |
| Vietnam | 40.9 (26.7-56.2) | 26.4 (16.4-38.6) |  | 76.6 (64.6-85.4) | 63.0 (49.0-75.2) |  | 86.9 (71.3-95.4) | 77.8 (56.3-91.4) |
| **Central Africa** |  |  |  |  |  |  |  |  |
| Angola | 32.1 (22.6-42.3) | 14.8 (10.3-20.3) |  | 61.0 (48.3-73.2) | 36.8 (26.8-48.8) |  | 73.2 (57.4-86.3) | 50.8 (33.1-69.5) |
| Cameroon | 64.1 (56.9-70.9) | 38.5 (31.5-45.4) |  | 82.0 (76.4-87.0) | 61.5 (53.6-69.0) |  | 87.6 (80.7-92.8) | 71.4 (59.9-81.3) |
| CAF | 58.2 (49.2-66.4) | 27.1 (21.1-33.8) |  | 78.0 (71.1-83.8) | 48.7 (39.5-57.5) |  | 84.7 (75.8-90.9) | 59.9 (45.3-73.0) |
| Chad | 29.5 (23.0-36.7) | 11.8 (8.9-15.2) |  | 38.5 (27.6-49.9) | 16.9 (11.5-23.4) |  | 43.4 (26.5-61.7) | 20.2 (10.6-32.2) |
| Congo | 71.3 (58.7-81.6) | 42.2 (29.9-55.3) |  | 89.1 (83.3-93.1) | 70.4 (59.6-79.7) |  | 93.2 (86.4-97.3) | 80.3 (64.0-91.6) |
| DRC | 12.4 (9.1-17.0) | 5.0 (3.6-6.7) |  | 76.0 (69.2-82.1) | 54.1 (45.3-62.1) |  | 93.4 (88.8-96.4) | 84.2 (75.1-90.7) |
| Sao TP | 75.3 (68.1-81.8) | 65.8 (57.3-73.7) |  | 95.6 (93.8-96.9) | 93.2 (90.9-95.1) |  | 98.3 (97.1-99.0) | 97.3 (95.5-98.4) |
| **East Africa** |  |  |  |  |  |  |  |  |
| Burundi | 73.6 (61.5-83.1) | 45.5 (32.9-57.3) |  | 97.3 (95.8-98.4) | 91.3 (87.1-94.4) |  | 99.2 (98.3-99.7) | 97.2 (94.3-98.9) |
| Comoros | 56.9 (45.0-67.0) | 39.7 (31.0-49.2) |  | 85.7 (76.2-92.2) | 75.1 (63.0-85.1) |  | 92.3 (82.8-97.2) | 85.9 (71.9-94.6) |
| Ethiopia | 33.8 (26.4-41.5) | 7.2 (5.2-9.5) |  | 60.6 (52.4-68.7) | 18.9 (14.6-24.2) |  | 72.5 (61.6-81.7) | 28.9 (19.6-40.0) |
| Kenya | 67.2 (59.2-74.1) | 43.9 (36.3-51.4) |  | 84.9 (77.2-90.8) | 68.4 (56.8-78.5) |  | 89.6 (79.2-95.8) | 77.0 (59.7-89.4) |
| Madagascar | 40.8 (32.1-48.8) | 22.3 (17.1-27.7) |  | 65.3 (56.2-73.7) | 44.1 (35.0-52.8) |  | 75.1 (63.0-85.0) | 56.0 (41.3-69.4) |
| Malawi | 41.0 (33.3-48.7) | 20.6 (16.3-25.7) |  | 92.2 (89.3-94.7) | 81.5 (76.1-86.3) |  | 97.9 (96.4-98.9) | 94.6 (91.2-97.0) |
| Mozambique | 79.3 (70.8-86.2) | 52.2 (41.3-62.5) |  | 91.6 (85.5-95.8) | 75.7 (60.1-86.3) |  | 94.3 (85.2-98.4) | 83.0 (60.8-94.2) |
| Rwanda | 79.6 (73.8-84.9) | 62.4 (54.6-69.6) |  | 97.6 (96.2-98.5) | 94.5 (91.8-96.3) |  | 99.2 (98.3-99.6) | 98.1 (96.3-99.1) |
| Tanzania | 74.1 (63.0-82.8) | 49.0 (36.2-61.3) |  | 82.2 (74.2-88.3) | 60.6 (49.8-71.2) |  | 84.8 (72.0-93.2) | 65.6 (47.0-81.8) |
| Uganda | 50.0 (40.5-59.7) | 25.5 (19.3-32.5) |  | 90.4 (86.3-93.7) | 76.3 (68.3-83.3) |  | 96.5 (93.7-98.3) | 90.5 (83.9-95.3) |
| Zambia | 72.6 (63.7-80.3) | 41.9 (33.6-51.0) |  | 92.9 (90.0-95.1) | 77.9 (70.8-83.7) |  | 96.5 (94.1-98.1) | 88.4 (81.3-93.5) |
| Zimbabwe | 59.9 (44.6-73.6) | 38.8 (24.9-52.6) |  | 94.6 (92.7-96.3) | 88.0 (83.8-91.4) |  | 98.3 (96.8-99.2) | 96.0 (92.4-98.2) |
| **West Africa** |  |  |  |  |  |  |  |  |
| Benin | 83.8 (75.0-89.8) | 72.4 (61.3-81.7) |  | 86.0 (80.2-90.2) | 75.6 (68.7-81.9) |  | 85.8 (74.9-93.0) | 75.6 (60.3-86.8) |
| Burkina Faso | 70.2 (59.6-79.5) | 41.9 (31.2-54.0) |  | 95.2 (90.7-98.1) | 86.1 (75.1-93.6) |  | 98.1 (94.4-99.6) | 94.1 (84.2-98.7) |
| Cote d'Ivoire | 78.2 (71.1-84.0) | 54.6 (45.7-62.7) |  | 82.3 (74.6-88.4) | 61.1 (50.4-71.5) |  | 83.6 (72.0-91.6) | 63.7 (46.9-78.7) |
| The Gambia | 69.5 (62.2-75.9) | 52.7 (44.6-60.1) |  | 83.4 (78.5-87.2) | 71.1 (65.5-76.4) |  | 87.9 (82.4-92.1) | 78.1 (69.8-85.0) |
| Ghana | 65.1 (57.0-73.6) | 38.0 (30.5-46.0) |  | 89.7 (86.0-92.7) | 74.2 (67.2-80.1) |  | 94.8 (91.0-97.2) | 85.8 (77.0-91.6) |
| Guinea | 66.0 (54.6-76.9) | 35.3 (24.6-47.4) |  | 87.0 (81.1-91.9) | 65.0 (54.8-74.7) |  | 92.1 (84.9-96.7) | 76.8 (61.0-88.3) |
| Liberia | 40.3 (26.6-53.8) | 18.6 (11.2-28.2) |  | 84.8 (79.1-89.6) | 65.2 (57.0-72.5) |  | 93.8 (88.8-96.9) | 83.6 (73.1-90.7) |
| Mali | 58.4 (50.0-66.6) | 27.7 (22.0-34.4) |  | 78.2 (71.7-83.7) | 49.4 (40.4-57.3) |  | 84.7 (75.3-90.8) | 60.4 (45.2-72.5) |
| Niger | 61.8 (53.0-70.3) | 27.5 (21.1-34.8) |  | 73.8 (60.8-84.0) | 40.2 (26.9-54.1) |  | 78.0 (58.2-90.9) | 46.9 (25.7-69.4) |
| Nigeria | 32.5 (24.9-40.3) | 11.8 (8.6-15.3) |  | 68.0 (60.8-74.2) | 37.2 (30.5-43.9) |  | 81.3 (72.3-88.1) | 55.2 (42.4-68.0) |
| Senegal | 55.5 (44.8-65.8) | 34.6 (25.2-45.2) |  | 81.1 (77.2-84.8) | 64.4 (59.2-69.7) |  | 88.6 (83.0-92.8) | 76.6 (67.7-84.1) |
| Sierra Leone | 61.0 (53.4-67.9) | 38.3 (31.9-45.3) |  | 92.1 (89.7-94.2) | 82.2 (78.0-86.0) |  | 96.9 (95.3-98.1) | 92.5 (89.0-95.2) |
| Togo | 75.1 (68.5-80.5) | 54.8 (46.9-62.5) |  | 86.9 (82.0-90.4) | 72.8 (65.4-78.7) |  | 90.5 (84.2-94.4) | 79.5 (69.1-87.0) |

Note: CAF, Central African Republic; DRC, Democratic Republic of the Congo; Sao TP, Sao Tome and Principe

Supplementary Table S6: Coverage of age-appropriate DTP3 vaccine according to the place of residence in 41 LMICs in Asia and Sub-Saharan Africa, 2000-2030

| **Country** | **Predicted coverage (95% credible intervals) in year** | | | | | | | |
| --- | --- | --- | --- | --- | --- | --- | --- | --- |
|  | **2000** | |  | **2020** | |  | **2030** | |
|  | **Urban** | **Rural** |  | **Urban** | **Rural** |  | **Urban** | **Rural** |
| **South Asia** |  |  |  |  |  |  |  |  |
| Bangladesh | 31.8 (25.2-39.6) | 19.9 (15.6-24.8) |  | 59.8 (51.0-67.4) | 44.3 (35.7-52.8) |  | 72.4 (60.2-81.8) | 58.5 (44.3-71.0) |
| India | 26.0 (15.4-40.3) | 15.8 (9.2-26.0) |  | 40.8 (27.6-54.3) | 27.0 (17.2-38.3) |  | 49.1 (26.2-72.0) | 34.7 (16.0-57.5) |
| Nepal | 23.3 (17.5-29.7) | 15.1 (11.2-19.9) |  | 57.6 (49.7-66.2) | 44.3 (36.6-52.3) |  | 73.9 (63.0-83.8) | 62.6 (49.8-73.9) |
| Pakistan | 5.1 (1.1-14.6) | 2.6 (0.5-7.4) |  | 43.8 (30.9-56.4) | 27.7 (18.7-38.0) |  | 74.7 (46.9-91.8) | 60.4 (30.0-84.5) |
| **Southeast Asia** |  |  |  |  |  |  |  |  |
| Cambodia | 19.0 (13.7-24.9) | 8.2 (5.9-10.9) |  | 86.8 (80.4-91.7) | 71.5 (61.6-80.0) |  | 97.1 (94.3-98.8) | 92.8 (86.5-96.7) |
| Laos | 17.1 (12.7-23.0) | 4.5 (3.2-6.2) |  | 58.2 (47.9-68.5) | 24.5 (17.5-32.1) |  | 78.0 (65.6-87.5) | 45.8 (30.4-61.3) |
| Myanmar | 16.7 (11.3-23.7) | 10.2 (7.0-14.0) |  | 32.5 (21.8-43.7) | 21.5 (14.3-30.2) |  | 42.6 (25.6-60.9) | 29.9 (16.6-46.6) |
| Timor-Leste | 10.0 (3.4-22.3) | 5.0 (1.6-11.2) |  | 58.5 (41.6-72.3) | 40.3 (26.2-55.1) |  | 82.3 (58.0-95.1) | 69.8 (40.8-90.3) |
| Vietnam | 33.4 (19.9-49.8) | 22.2 (12.3-35.8) |  | 32.7 (20.0-48.0) | 21.6 (12.7-33.9) |  | 33.2 (12.1-61.6) | 22.5 (7.4-46.6) |
| **Central Africa** |  |  |  |  |  |  |  |  |
| Angola | 9.3 (6.0-13.5) | 3.8 (2.5-5.6) |  | 38.2 (26.9-50.7) | 19.3 (13.2-26.9) |  | 59.5 (40.5-77.0) | 36.9 (21.4-54.5) |
| Cameroon | 36.2 (29.0-43.7) | 18.3 (14.1-22.9) |  | 68.2 (60.0-75.6) | 45.9 (37.7-53.9) |  | 80.3 (70.2-88.0) | 62.0 (48.7-73.6) |
| CAF | 15.2 (10.8-20.2) | 5.5 (4.0-7.4) |  | 29.4 (21.3-38.6) | 12.1 (8.6-16.6) |  | 39.2 (25.4-54.7) | 17.7 (10.3-27.4) |
| Chad | 10.0 (7.0-13.7) | 4.2 (3.0-5.7) |  | 15.3 (9.7-22.0) | 6.7 (4.3-10.2) |  | 19.1 (9.4-32.2) | 8.7 (4.1-16.1) |
| Congo | 39.9 (26.0-54.1) | 19.1 (10.8-29.3) |  | 48.6 (35.0-62.3) | 25.2 (16.5-35.9) |  | 52.7 (28.7-75.8) | 29.2 (13.0-52.1) |
| DRC | 9.9 (6.9-13.8) | 4.0 (2.8-5.5) |  | 48.2 (39.4-57.5) | 26.4 (20.0-33.2) |  | 72.3 (59.1-83.7) | 50.5 (36.3-65.0) |
| Sao TP | 31.6 (23.1-40.5) | 23.7 (17.2-31.5) |  | 83.5 (77.2-88.2) | 77.3 (71.0-82.5) |  | 94.3 (90.2-96.9) | 91.8 (86.9-95.3) |
| **East Africa** |  |  |  |  |  |  |  |  |
| Burundi | 32.2 (20.9-44.0) | 17.3 (11.0-25.0) |  | 86.9 (80.4-91.7) | 74.7 (65.0-82.8) |  | 96.0 (91.8-98.4) | 91.4 (83.1-96.4) |
| Comoros | 20.7 (14.1-28.6) | 10.9 (7.4-14.9) |  | 57.1 (41.6-71.2) | 38.9 (25.5-53.1) |  | 74.3 (54.4-89.2) | 58.5 (35.2-78.4) |
| Ethiopia | 31.4 (24.5-38.9) | 6.3 (4.5-8.6) |  | 63.0 (55.2-70.9) | 20.1 (15.4-25.9) |  | 76.5 (66.6-84.7) | 32.8 (22.7-45.4) |
| Kenya | 56.4 (47.3-64.9) | 36.0 (28.8-43.8) |  | 76.7 (65.2-85.8) | 59.1 (45.9-71.6) |  | 83.6 (67.9-94.0) | 69.6 (47.3-86.4) |
| Madagascar | 35.5 (27.5-43.3) | 18.4 (13.7-23.4) |  | 55.0 (44.6-64.5) | 33.5 (25.4-42.3) |  | 64.1 (48.8-77.3) | 42.7 (28.0-58.2) |
| Malawi | 27.7 (21.3-34.7) | 13.4 (10.1-16.9) |  | 74.7 (67.1-81.4) | 54.4 (45.4-63.2) |  | 88.9 (82.0-93.7) | 76.5 (64.8-85.7) |
| Mozambique | 26.6 (17.2-37.2) | 10.2 (6.3-15.2) |  | 44.2 (26.3-64.5) | 20.3 (9.9-35.4) |  | 53.5 (23.3-82.2) | 28.5 (8.6-58.7) |
| Rwanda | 56.1 (46.7-64.7) | 40.8 (32.9-49.1) |  | 90.4 (85.6-94.1) | 83.5 (76.7-89.4) |  | 96.1 (92.3-98.3) | 92.9 (86.5-97.0) |
| Tanzania | 43.8 (30.1-57.9) | 24.0 (15.3-35.1) |  | 69.9 (57.8-80.1) | 48.5 (36.9-61.2) |  | 79.3 (61.8-91.3) | 61.4 (41.3-80.2) |
| Uganda | 24.8 (18.1-33.3) | 11.8 (8.5-16.3) |  | 66.2 (55.1-76.0) | 44.5 (32.9-54.4) |  | 82.3 (69.8-91.2) | 65.7 (46.9-80.3) |
| Zambia | 16.4 (11.3-22.7) | 7.0 (4.7-10.0) |  | 65.7 (56.3-74.6) | 42.6 (33.8-52.0) |  | 85.5 (75.8-92.2) | 69.7 (55.4-81.7) |
| Zimbabwe | 0.0 (0.0-0.1) | 0.0 (0.0-0.0) |  | 90.8 (87.0-93.6) | 83.8 (78.4-88.2) |  | 99.9 (99.9-100.0) | 99.9 (99.8-100.0) |
| **West Africa** |  |  |  |  |  |  |  |  |
| Benin | 33.8 (20.7-48.3) | 21.9 (13.0-33.3) |  | 46.5 (36.9-57.2) | 32.3 (24.1-41.6) |  | 53.5 (33.8-72.9) | 39.1 (21.7-59.2) |
| Burkina Faso | 16.4 (10.0-23.9) | 9.0 (5.7-13.2) |  | 62.3 (42.0-79.9) | 46.1 (26.4-67.3) |  | 81.0 (53.7-95.4) | 69.5 (36.3-91.7) |
| Cote d'Ivoire | 33.8 (25.8-43.5) | 18.8 (13.9-24.2) |  | 41.8 (29.8-54.1) | 24.6 (16.5-33.9) |  | 45.9 (27.5-64.5) | 28.2 (14.6-44.2) |
| The Gambia | 10.4 (7.6-13.8) | 8.0 (5.8-10.5) |  | 38.8 (31.4-46.3) | 32.1 (25.8-38.4) |  | 59.4 (47.4-70.4) | 52.4 (40.4-63.6) |
| Ghana | 26.1 (18.9-34.2) | 14.1 (10.0-19.2) |  | 74.1 (66.2-81.0) | 57.2 (48.4-65.3) |  | 88.9 (81.1-94.1) | 78.9 (67.0-87.9) |
| Guinea | 28.2 (17.0-39.5) | 12.4 (7.2-19.4) |  | 35.8 (25.1-47.2) | 16.8 (10.9-23.8) |  | 40.4 (20.5-61.0) | 20.1 (8.8-36.3) |
| Liberia | 13.9 (8.0-22.0) | 5.4 (3.1-8.8) |  | 52.2 (42.5-62.0) | 28.0 (21.2-35.6) |  | 73.8 (58.7-85.2) | 50.6 (34.1-66.6) |
| Mali | 26.1 (19.4-33.8) | 11.4 (8.1-15.1) |  | 47.4 (39.1-56.3) | 24.8 (19.2-32.0) |  | 58.8 (45.2-72.2) | 34.6 (22.8-49.5) |
| Niger | 26.8 (19.9-34.6) | 11.3 (8.1-15.5) |  | 38.4 (24.7-53.5) | 18.1 (10.3-28.1) |  | 44.8 (22.3-68.6) | 23.0 (9.3-42.4) |
| Nigeria | 13.4 (9.6-18.1) | 4.9 (3.4-6.7) |  | 45.3 (37.9-53.6) | 21.6 (16.9-27.1) |  | 65.4 (51.7-77.0) | 39.0 (26.4-51.8) |
| Senegal | 30.2 (21.3-40.0) | 14.7 (9.9-20.7) |  | 55.4 (49.2-61.5) | 33.1 (27.7-38.7) |  | 67.7 (56.8-77.4) | 45.7 (34.0-57.4) |
| Sierra Leone | 19.9 (15.2-25.7) | 9.7 (7.3-12.8) |  | 48.3 (40.6-55.6) | 28.9 (23.1-35.0) |  | 64.5 (52.2-75.0) | 44.3 (32.5-55.9) |
| Togo | 22.2 (16.5-29.1) | 11.4 (8.4-15) |  | 66.6 (58.3-74.7) | 47.4 (38.9-55.7) |  | 83.8 (74.8-90.6) | 70.2 (56.9-80.8) |

Note: CAF, Central African Republic; DRC, Democratic Republic of the Congo; Sao TP, Sao Tome and Principe

Supplementary Table S7: Coverage of age-appropriate polio3 vaccine according to the place of residence in 41 LMICs in Asia and Sub-Saharan Africa, 2000-2030

| **Country** | **Predicted coverage (95% credible intervals) in year** | | | | | | | |
| --- | --- | --- | --- | --- | --- | --- | --- | --- |
|  | **2000** | |  | **2020** | |  | **2030** | |
|  | **Urban** | **Rural** |  | **Urban** | **Rural** |  | **Urban** | **Rural** |
| **South Asia** |  |  |  |  |  |  |  |  |
| Bangladesh | 32.7 (26.5-38.8) | 21.0 (16.7-25.9) |  | 59.0 (50.7-66.6) | 44.2 (37.2-51.7) |  | 71.1 (59.5-80.5) | 57.6 (45.4-69.1) |
| India | 34.2 (22.4-48.1) | 22.3 (14.1-32.9) |  | 38.5 (26.6-51.1) | 25.6 (16.7-36.2) |  | 41.1 (21.9-63.3) | 28.1 (13.1-47.2) |
| Nepal | 36.4 (29.4-44.0) | 26.6 (20.9-32.6) |  | 52.9 (45.1-60.5) | 41.6 (34.6-48.5) |  | 61.2 (49.4-71.7) | 50.1 (38.3-61.1) |
| Pakistan | 6.5 (1.5-17.7) | 4.0 (0.8-10.3) |  | 49.5 (37.4-62.1) | 36.6 (26.0-47.9) |  | 78.4 (56.2-93.6) | 68.7 (39.9-89.4) |
| **Southeast Asia** |  |  |  |  |  |  |  |  |
| Cambodia | 20.6 (15.7-27.0) | 9.1 (6.8-12.0) |  | 85.6 (79.4-90.4) | 69.7 (60.6-78.3) |  | 96.5 (93.7-98.3) | 91.5 (85.0-95.8) |
| Laos | 18.3 (13.5-23.9) | 4.9 (3.6-6.5) |  | 62.0 (52.3-70.7) | 27.7 (20.8-35.1) |  | 81.3 (71.2-88.9) | 50.9 (36.7-64.4) |
| Myanmar | 17.7 (12.1-24.6) | 11.6 (8.2-15.9) |  | 33.8 (23.9-44.6) | 23.9 (16.4-32.8) |  | 44.0 (28.1-60.9) | 32.8 (19.2-49.2) |
| Timor-Leste | 11.2 (4.1-23.8) | 6.1 (2.2-13.3) |  | 51.6 (37.5-67.2) | 35.7 (23.2-49.8) |  | 74.8 (47.4-92.6) | 61.7 (31.2-85.8) |
| Vietnam | 36.6 (23.4-50.9) | 26.0 (15.5-38.4) |  | 38.7 (26.0-52.6) | 27.8 (17.5-39.9) |  | 40.3 (18.7-65.4) | 29.6 (12.0-54.6) |
| **Central Africa** |  |  |  |  |  |  |  |  |
| Angola | 11.2 (7.5-15.9) | 5.1 (3.5-7.2) |  | 34.5 (24.4-45.9) | 18.5 (12.5-26.0) |  | 51.5 (33.7-69.1) | 31.7 (18.7-48.7) |
| Cameroon | 34.5 (28.4-40.8) | 19.1 (15.0-23.8) |  | 71.0 (63.5-77.0) | 52.3 (44.7-59.9) |  | 83.9 (75.3-89.7) | 70.1 (58.8-79.4) |
| CAR | 15.0 (11.0-19.7) | 5.9 (4.4-7.7) |  | 38.3 (29.8-47.2) | 18.2 (13.6-23.8) |  | 54.0 (40.3-67.4) | 30.0 (19.8-42.9) |
| Chad | 11.5 (8.4-14.9) | 5.4 (4.0-7.0) |  | 20.6 (14.2-28.1) | 10.2 (7.0-14.3) |  | 27.1 (15.8-42.0) | 14.1 (7.7-23.4) |
| Congo | 44.7 (32.4-57.6) | 25.7 (16.8-36.2) |  | 44.9 (32.2-57.8) | 25.8 (17.7-35.0) |  | 45.2 (24.4-66.8) | 26.5 (12.4-44.8) |
| DRC | 8.3 (6.0-11.3) | 3.9 (2.8-5.2) |  | 62.2 (54.1-69.9) | 42.2 (34.4-50.1) |  | 87.2 (80.1-92.5) | 75.3 (64.6-84.7) |
| Sao TP | 28.6 (21.8-36.6) | 21.0 (15.9-26.6) |  | 83.0 (78.4-87.3) | 76.5 (70.7-81.9) |  | 94.5 (91.5-96.7) | 92.0 (87.4-95.2) |
| **East Africa** |  |  |  |  |  |  |  |  |
| Burundi | 41.2 (29.9-53.0) | 23.5 (15.7-33.2) |  | 84.3 (77.5-89.8) | 70.2 (60.9-78.5) |  | 93.4 (87.6-97.2) | 86.3 (75.6-93.4) |
| Comoros | 19.6 (13.6-26.2) | 10.7 (7.5-14.3) |  | 61.4 (46.6-74.5) | 44.2 (31.6-58.5) |  | 79.6 (60.8-91.4) | 66.4 (45.4-83.8) |
| Ethiopia | 43.5 (35.6-51.3) | 11.0 (8.3-14.2) |  | 61.9 (53.8-69.0) | 20.7 (16.2-25.7) |  | 70.2 (59.2-79.0) | 27.6 (19.2-37.8) |
| Kenya | 48.0 (40.2-55.8) | 30.4 (24.3-36.6) |  | 77.9 (68.2-85.2) | 62.7 (51.2-73.1) |  | 87.3 (76.1-94.5) | 76.8 (59.3-89.5) |
| Madagascar | 33.7 (26.8-41.2) | 17.7 (13.7-22.6) |  | 58.6 (48.8-67.8) | 37.5 (29.4-45.8) |  | 70.0 (56.0-81.0) | 50.1 (36.3-63.8) |
| Malawi | 27.0 (21.1-33.3) | 13.8 (11.0-17.6) |  | 67.4 (59.5-74.6) | 47.4 (39.5-55.5) |  | 82.8 (74.5-89.5) | 67.9 (55.6-78.7) |
| Mozambique | 25.2 (17.3-34.7) | 9.7 (6.4-14.3) |  | 45.7 (28.8-62.8) | 21.6 (11.6-35.5) |  | 56.6 (28.2-81.3) | 31.1 (11.7-59.0) |
| Rwanda | 57.8 (49.1-66.0) | 42.6 (35.0-50.5) |  | 90.0 (85.4-93.5) | 83.1 (76.5-88.5) |  | 95.8 (91.9-98.1) | 92.5 (85.8-96.5) |
| Tanzania | 42.1 (30.8-55.2) | 23.5 (15.6-32.8) |  | 69.2 (59.1-78.3) | 48.7 (37.8-59.2) |  | 79.3 (64.5-90.3) | 62.2 (42.9-78.4) |
| Uganda | 27.5 (21.0-35.3) | 13.7 (10.3-17.8) |  | 62.3 (52.1-71.7) | 41.1 (31.9-50.6) |  | 77.3 (64.4-87.3) | 59.2 (43.2-73.5) |
| Zambia | 17.6 (12.7-23.3) | 7.6 (5.5-10.3) |  | 63.0 (54.2-70.5) | 39.8 (32.3-47.9) |  | 82.6 (73.1-89.3) | 65.0 (51.7-76.2) |
| Zimbabwe | 0.0 (0.0-0.1) | 0.0 (0.0-0.0) |  | 90.5 (86.9-93.1) | 83.3 (78.3-87.6) |  | 99.9 (99.9-100.0) | 99.9 (99.8-99.9) |
| **West Africa** |  |  |  |  |  |  |  |  |
| Benin | 38.3 (25.9-50.8) | 25.5 (16.4-35.8) |  | 46.5 (37.7-55.5) | 32.4 (24.8-40.7) |  | 51.4 (33.9-68.7) | 37.1 (22.1-54.0) |
| Burkina Faso | 16.5 (11.2-24.0) | 9.5 (6.3-13.5) |  | 63.1 (44.1-80.1) | 48.3 (29.7-66.9) |  | 82.1 (58.4-95.3) | 71.9 (42.2-90.7) |
| Cote d'Ivoire | 33.5 (25.9-41.3) | 20.1 (15.4-25.4) |  | 50.1 (39.0-61.9) | 33.5 (24.9-42.5) |  | 58.5 (41.7-73.6) | 41.7 (26.2-57.3) |
| The Gambia | 12.3 (9.3-16.0) | 10.1 (7.7-13.0) |  | 37.2 (31.1-44.0) | 32.2 (26.9-38.1) |  | 55.0 (44.6-65.6) | 49.5 (39.0-60.3) |
| Ghana | 36.7 (29.0-44.8) | 20.5 (15.7-26.7) |  | 70.9 (63.5-77.8) | 52.0 (43.9-60.4) |  | 83.1 (73.3-90.1) | 68.8 (56.0-79.8) |
| Guinea | 29.9 (19.1-42.5) | 13.6 (8.1-20.7) |  | 41.9 (31.2-53.6) | 21.1 (14.0-29.1) |  | 48.6 (29.2-69.8) | 26.5 (13.1-44.8) |
| Liberia | 17.8 (10.8-26.8) | 7.8 (4.9-11.9) |  | 50.3 (41.4-59.2) | 28.4 (21.8-34.7) |  | 68.9 (54.3-80.5) | 46.9 (32.1-61.6) |
| Mali | 28.9 (22.7-35.8) | 13.2 (9.8-16.7) |  | 45.3 (37.3-53.7) | 23.6 (18.3-29.7) |  | 54.2 (40.7-67.0) | 30.9 (20.1-42.2) |
| Niger | 27.2 (20.7-34.5) | 12.8 (9.5-17.1) |  | 40.3 (26.5-54.8) | 21.2 (12.7-31.1) |  | 47.6 (25.4-69.2) | 27.1 (12.1-46.5) |
| Nigeria | 19.2 (14.7-24.5) | 8.2 (6.1-10.9) |  | 47.7 (40.5-54.8) | 25.7 (20.7-30.7) |  | 64.0 (52.4-74.7) | 40.4 (29.4-52.0) |
| Senegal | 30.9 (22.8-40.0) | 15.3 (10.7-21.0) |  | 54.8 (48.8-60.6) | 32.8 (27.9-38.0) |  | 66.6 (56.1-76.0) | 44.7 (34.2-56.1) |
| Sierra Leone | 27.2 (21.6-33.2) | 14.2 (11.2-17.7) |  | 46.8 (39.8-53.9) | 28.0 (23.1-33.5) |  | 57.7 (46.8-67.8) | 37.8 (28.4-48.1) |
| Togo | 26.1 (20.6-32.7) | 13.7 (10.4-17.5) |  | 70.5 (62.6-77.2) | 52.0 (44.1-59.5) |  | 86.0 (78.6-91.5) | 73.7 (62.7-82.6) |

Note: CAF, Central African Republic; DRC, Democratic Republic of the Congo; Sao TP, Sao Tome and Principe

Supplementary Table S8: Coverage of age-appropriate MCV according to the place of residence in 41 LMICs in Asia and Sub-Saharan Africa, 2000-2030

| **Country** | **Predicted coverage (95% credible intervals) in year** | | | | | | | | |
| --- | --- | --- | --- | --- | --- | --- | --- | --- | --- |
|  | **2000** | |  | **2020** | |  | **2030** | |  |
|  | **Urban** | **Rural** |  | **Urban** | **Rural** |  | **Urban** | **Rural** |  |
| **South Asia** |  |  |  |  |  |  |  |  |  |
| Bangladesh | 46.5 (39.2-53.7) | 38.1 (31.8-44.5) |  | 69.6 (62.6-75.8) | 61.9 (54.5-68.8) |  | 78.6 (69.4-85.9) | 72.3 (61.7-80.7) |  |
| India | 28.1 (18.0-40.1) | 18.0 (11.2-26.2) |  | 51.6 (38.9-63.3) | 37.6 (27.0-48.4) |  | 63.4 (43.0-79.7) | 49.9 (30.3-68.2) |  |
| Nepal | 23.6 (18.0-30.1) | 15.5 (11.6-19.9) |  | 72.7 (66.8-78.5) | 61.4 (53.9-68.2) |  | 88.6 (83.5-92.8) | 82.3 (74.1-88.4) |  |
| Pakistan | 10.6 (3.6-22.3) | 5.8 (1.9-13.2) |  | 46.0 (34.9-56.4) | 30.8 (22.9-39.7) |  | 69.6 (47.8-86.0) | 55.1 (32.7-75.6) |  |
| **Southeast Asia** |  |  |  |  |  |  |  |  |  |
| Cambodia | 27.1 (20.9-33.7) | 15.0 (11.4-19.2) |  | 84.9 (78.5-90.1) | 72.9 (64.4-80.5) |  | 95.5 (91.7-97.9) | 91.1 (84.4-95.5) |  |
| Laos | 17.3 (12.8-22.2) | 6.6 (4.9-8.7) |  | 63.6 (54.9-71.4) | 37.6 (29.6-46.3) |  | 83.3 (74.4-90.0) | 63.3 (49.8-76.1) |  |
| Myanmar | 43.4 (34.0-52.8) | 26.9 (20.4-34.2) |  | 50.1 (38.9-60.4) | 32.6 (23.8-42.5) |  | 53.3 (37.6-68.8) | 35.8 (22.3-51.8) |  |
| Timor-Leste | 21.4 (10.9-36.8) | 13.8 (6.8-24.0) |  | 54.8 (42.1-67.1) | 41.7 (30.9-53.0) |  | 71.3 (49.6-87.5) | 60.0 (36.9-80.2) |  |
| Vietnam | 59.0 (45.6-72.1) | 45.0 (32.5-59.1) |  | 59.6 (46.0-71.6) | 45.6 (32.8-57.6) |  | 59.5 (34.8-79.1) | 46.0 (24.0-67.5) |  |
| **Central Africa** |  |  |  |  |  |  |  |  |  |
| Angola | 11.5 (7.9-16.4) | 5.2 (3.5-7.1) |  | 36.2 (26.0-47.2) | 19.4 (13.3-26.5) |  | 53.9 (36.2-69.9) | 33.4 (20.0-48.5) |  |
| Cameroon | 45.5 (38.4-52.4) | 26.0 (21.1-31.0) |  | 62.8 (55.8-69.5) | 41.5 (34.7-48.3) |  | 70.4 (59.8-79.4) | 50.2 (38.6-61.1) |  |
| CAR | 19.5 (14.4-24.5) | 8.1 (6.0-10.6) |  | 29.0 (22.3-36.3) | 13.0 (9.7-17.1) |  | 35.0 (24.0-46.9) | 16.5 (10.6-24.5) |  |
| Chad | 8.6 (6.3-11.7) | 3.6 (2.7-4.9) |  | 22.8 (16.1-30.9) | 10.7 (7.3-15.1) |  | 34.5 (21.3-50.6) | 17.7 (9.8-28.9) |  |
| Congo | 42.3 (30.8-55.1) | 22.8 (15.5-32.9) |  | 50.1 (38.1-61.6) | 28.7 (20.4-38.1) |  | 53.6 (31.5-73.0) | 32.3 (16.7-51.3) |  |
| DRC | 9.7 (7.0-12.7) | 4.7 (3.5-6.3) |  | 42.1 (34.2-50.1) | 25.0 (19.5-30.9) |  | 65.0 (51.4-76.3) | 46.4 (33.6-59.3) |  |
| Sao TP | 38.7 (30.8-46.8) | 32.7 (26.3-40.3) |  | 73.1 (66.1-79.5) | 67.7 (61.0-74.3) |  | 84.8 (77.7-90.8) | 81.2 (73.5-88.0) |  |
| **East Africa** |  |  |  |  |  |  |  |  |  |
| Burundi | 50.9 (39.3-62.0) | 38.0 (28.2-49.2) |  | 82.9 (76.6-88.3) | 74.1 (66.3-80.9) |  | 91.0 (84.1-95.6) | 85.7 (76.0-92.4) |  |
| Comoros | 28.2 (20.8-36.8) | 18.0 (13.3-23.6) |  | 49.5 (35.9-62.9) | 35.6 (24.5-47.8) |  | 60.3 (41.0-78.2) | 46.5 (27.8-65.5) |  |
| Ethiopia | 28.3 (21.7-34.7) | 7.1 (5.3-9.3) |  | 53.6 (45.6-61.4) | 18.4 (14.0-23.3) |  | 66.3 (54.7-75.7) | 27.9 (19.0-38.0) |  |
| Kenya | 51.5 (43.6-59.5) | 34.0 (28.0-40.5) |  | 65.2 (54.1-75.1) | 47.9 (36.1-59.5) |  | 70.7 (52.7-85.0) | 54.6 (35.0-72.8) |  |
| Madagascar | 42.8 (35.0-50.6) | 23.3 (18.6-28.5) |  | 55.1 (46.5-64.1) | 33.4 (25.8-41.2) |  | 60.9 (48.3-73.7) | 39.1 (26.5-52.5) |  |
| Malawi | 33.6 (27.6-40.6) | 24.0 (19.2-29.1) |  | 60.5 (51.8-68.5) | 48.9 (40.6-57.3) |  | 72.5 (60.5-82.0) | 62.3 (49.2-74.0) |  |
| Mozambique | 54.2 (43.2-64.5) | 29.8 (21.8-38.5) |  | 69.0 (54.8-80.7) | 44.7 (30.3-58.1) |  | 74.5 (53.0-89.8) | 52.4 (28.4-73.6) |  |
| Rwanda | 60.0 (51.9-68.3) | 47.3 (39.5-55.4) |  | 83.0 (71.9-90.4) | 74.6 (60.6-84.6) |  | 89.3 (76.4-96.1) | 83.5 (65.9-93.6) |  |
| Tanzania | 50.5 (38.9-62.4) | 32.0 (22.8-41.3) |  | 67.3 (57.0-76.6) | 48.7 (38.9-58.9) |  | 74.1 (59.3-86.7) | 57.2 (39.8-74.3) |  |
| Uganda | 28.2 (21.9-34.9) | 16.7 (12.8-21.2) |  | 60.3 (50.8-69.4) | 43.9 (34.7-52.5) |  | 74.7 (61.6-85.3) | 60.5 (45.4-73.2) |  |
| Zambia | 43.1 (34.5-51.6) | 26.8 (20.6-33.9) |  | 65.8 (57.9-73.5) | 48.1 (40.5-56.0) |  | 75.2 (64.4-84.3) | 59.6 (48.0-71.1) |  |
| Zimbabwe | 41.5 (28.6-55.2) | 24.5 (15.6-35.6) |  | 77.0 (71.0-82.4) | 60.4 (52.2-67.4) |  | 87.7 (79.5-93.4) | 76.5 (63.4-86.7) |  |
| **West Africa** |  |  |  |  |  |  |  |  |  |
| Benin | 38.5 (28.0-50.1) | 26.0 (17.9-35.9) |  | 57.9 (48.8-66.6) | 43.6 (35.5-51.6) |  | 66.6 (50.5-79.6) | 53.1 (35.7-68.2) |  |
| Burkina Faso | 37.3 (27.3-47.2) | 22.3 (15.7-29.8) |  | 80.8 (68.9-90.3) | 67.4 (51.8-81.5) |  | 91.2 (79.5-97.8) | 83.7 (65.1-95.2) |  |
| Cote d'Ivoire | 42.3 (34.1-51.1) | 27.5 (21.3-34.6) |  | 48.0 (36.9-58.1) | 32.4 (24.0-41.6) |  | 50.7 (34.0-65.7) | 35.0 (20.9-50.4) |  |
| The Gambia | 42.9 (35.3-50.7) | 37.5 (30.8-44.8) |  | 60.1 (53.1-66.6) | 54.6 (48.0-60.6) |  | 67.9 (58.0-76.6) | 62.9 (52.8-71.8) |  |
| Ghana | 46.6 (38.4-54.5) | 31.2 (24.5-38.4) |  | 68.5 (61.3-75.1) | 53.1 (45.2-60.7) |  | 77.2 (66.5-85.5) | 64.0 (51.4-75.2) |  |
| Guinea | 29.6 (19.6-40.6) | 15.7 (10.2-22.0) |  | 34.3 (25.3-43.5) | 18.8 (13.2-25.1) |  | 37.1 (21.5-53.6) | 21.0 (10.8-33.7) |  |
| Liberia | 19.7 (12.3-29.0) | 9.3 (5.5-14.3) |  | 53.5 (44.5-62.1) | 32.5 (25.6-39.8) |  | 71.1 (58.5-82.6) | 51.2 (36.7-65.3) |  |
| Mali | 31.4 (24.7-39.1) | 15.4 (11.8-19.6) |  | 51.6 (43.4-59.8) | 29.9 (24.0-37.1) |  | 61.8 (49.3-73.3) | 39.5 (28.6-52.8) |  |
| Niger | 36.5 (28.6-44.8) | 16.9 (12.4-21.8) |  | 49.8 (36.2-62.6) | 26.2 (17.2-37.4) |  | 56.3 (35.7-75.1) | 32.1 (17.0-51.9) |  |
| Nigeria | 15.9 (12.1-20.8) | 5.9 (4.3-7.8) |  | 45.5 (38.3-52.2) | 21.7 (17.3-26.5) |  | 63.6 (51.6-74.0) | 36.9 (26.8-48.5) |  |
| Senegal | 36.2 (27.8-45.5) | 22.9 (16.6-30.1) |  | 62.7 (56.6-68.0) | 46.7 (41.2-52.1) |  | 74.1 (64.7-82.3) | 60.1 (49.3-70.3) |  |
| Sierra Leone | 27.5 (21.9-33.4) | 16.2 (12.8-20.0) |  | 55.5 (48.4-61.9) | 38.9 (32.9-45.1) |  | 69.2 (58.7-77.3) | 53.5 (42.4-63.8) |  |
| Togo | 29.6 (23.7-36.0) | 18.1 (14.0-22.7) |  | 61.5 (53.4-68.9) | 45.7 (37.8-53.5) |  | 75.4 (65.1-83.9) | 62.0 (49.0-72.9) |  |

Note: CAF, Central African Republic; DRC, Democratic Republic of the Congo; Sao TP, Sao Tome and Principe

Supplementary Table S9: Coverage of age-appropriate BCG vaccine across the poorest and richest quintile in 41 LMICs in Asia and Sub-Saharan Africa, 2000-2030

| **Country** | **Predicted coverage (95% credible intervals) in year** | | | | | | | |
| --- | --- | --- | --- | --- | --- | --- | --- | --- |
|  | **2000** | |  | **2020** | |  | **2030** | |
|  | **Poorest** | **Richest** |  | **Poorest** | **Richest** |  | **Poorest** | **Richest** |
| **South Asia** |  |  |  |  |  |  |  |  |
| Bangladesh | 10.9 (8.5-13.7) | 12.0 (9.3-15.2) |  | 31.9 (26.2-37.7) | 34.2 (28.5-40.2) |  | 46.7 (36.7-56.5) | 49.3 (39.4-58.6) |
| India | 9.6 (6.2-14.2) | 34.6 (23.0-46.4) |  | 58.3 (48.1-68.5) | 87.3 (81.6-91.6) |  | 82.0 (70.6-90.6) | 95.7 (91.7-98.0) |
| Nepal | 10.9 (8.2-13.9) | 30.5 (23.8-36.5) |  | 48.2 (41.0-55.8) | 76.9 (71.0-81.9) |  | 68.0 (56.5-78.0) | 88.3 (82.3-92.6) |
| Pakistan | 4.3 (0.0-28.6) | 21.1 (0.5-81.5) |  | 37.3 (27.7-47.6) | 85.7 (79.5-90.4) |  | 70.1 (49.2-86.4) | 95.8 (91.1-98.5) |
| **Southeast Asia** |  |  |  |  |  |  |  |  |
| Cambodia | 10.1 (7.6-13.0) | 33.6 (26.6-40.7) |  | 83.7 (75.7-89.7) | 95.9 (93.3-97.6) |  | 96.2 (92.0-98.4) | 99.1 (98.2-99.6) |
| Laos | 6.4 (4.7-8.5) | 28.4 (22.4-34.9) |  | 52.7 (43.0-61.8) | 86.5 (81.0-90.6) |  | 81.1 (69.6-88.9) | 96.1 (93.1-98.0) |
| Myanmar | 35.0 (27.8-43.3) | 62.1 (52.9-70.4) |  | 48.1 (32.9-63.8) | 73.5 (59.9-83.9) |  | 56.8 (21.5-86.0) | 78.0 (46.7-95.0) |
| Timor-Leste | 15.5 (0.6-68.0) | 40.0 (3.0-93.1) |  | 44.5 (30.9-58.2) | 81.8 (71.7-89.4) |  | 68.5 (37.8-89.8) | 91.9 (78.6-98.2) |
| Vietnam | 13.5 (5.2-24.7) | 37.7 (18.5-57.6) |  | 54.8 (42.5-65.7) | 82.8 (75.6-88.8) |  | 74.7 (55.3-88.2) | 92.0 (84.1-96.9) |
| **Central Africa** |  |  |  |  |  |  |  |  |
| Angola | 8.4 (5.0-12.7) | 43.6 (31.2-56.0) |  | 35.3 (24.2-47.7) | 82.0 (72.8-89.1) |  | 53.9 (35.3-71.2) | 90.5 (82.5-95.6) |
| Cameroon | 23.2 (18.7-28.2) | 69.5 (62.9-74.9) |  | 48.6 (42.6-55.3) | 87.7 (84.6-90.5) |  | 48.7 (31.8-65.0) | 87.3 (78.4-93.3) |
| CAR | 22.0 (17.3-27.1) | 68.7 (62.4-75.3) |  | 37.0 (30.1-44.3) | 82.1 (76.9-86.1) |  | 52.2 (30.3-72.0) | 88.9 (78.0-95.4) |
| Chad | 8.0 (6.0-10.4) | 31.7 (25.2-38.9) |  | 13.1 (8.5-19.6) | 44.1 (32.6-57.8) |  | 12.0 (7.3-18.6) | 41.8 (28.9-54.2) |
| Congo | 39.2 (17.5-66.6) | 85.6 (69.7-95.3) |  | 60.5 (41.1-78.5) | 93.9 (88.2-97.4) |  | 71.0 (36.2-93.4) | 95.7 (86.0-99.3) |
| DRC | 1.7 (1.2-2.2) | 10.2 (7.7-13.1) |  | 21.5 (16.2-27.7) | 64.7 (57.1-71.6) |  | 12.5 (6.6-20.9) | 48.1 (31.8-64.5) |
| Sao TP | 64.3 (57.5-70.9) | 76.6 (70.3-81.7) |  | 93.4 (91.4-95.1) | 96.3 (95.1-97.2) |  | 97.7 (96.6-98.5) | 98.7 (98.0-99.2) |
| **East Africa** |  |  |  |  |  |  |  |  |
| Burundi | 24.9 (13.5-39.2) | 51.0 (32.3-68.8) |  | 91.3 (87.9-94.1) | 97.1 (95.7-98.1) |  | 97.0 (93.2-98.9) | 99.0 (97.7-99.6) |
| Comoros | 31.2 (24.2-39.1) | 60.9 (52.1-69.4) |  | 62.7 (36.5-83.8) | 84.4 (66.2-94.7) |  | 73.3 (34.2-95.3) | 89.1 (62.1-98.6) |
| Ethiopia | 3.6 (2.7-4.7) | 26.7 (21.0-32.7) |  | 14.2 (10.9-17.7) | 61.7 (54.3-68.3) |  | 24.5 (14.9-35.7) | 75.3 (63.1-84.8) |
| Kenya | 32.3 (26.5-38.6) | 73.4 (67.7-78.5) |  | 74.8 (62.1-85.2) | 94.4 (90.5-97.1) |  | 89.8 (73.1-97.8) | 98.0 (94.2-99.6) |
| Madagascar | 15.0 (11.4-19.2) | 51.1 (43.2-58.8) |  | 34.3 (26.4-42.5) | 75.4 (68-81.8.0) |  | 48.3 (33.7-61.5) | 84.4 (75.1-90.7) |
| Malawi | 17.3 (14.0-20.8) | 37.4 (31.5-43.3) |  | 76.9 (69.4-83.1) | 90.4 (86.8-93.5) |  | 89.4 (76.7-96.1) | 96.0 (90.5-98.6) |
| Mozambique | 35.8 (24.2-48.5) | 82.6 (73.9-89.2) |  | 64.9 (35.1-86.8) | 93.6 (82.2-98.4) |  | 68.4 (22.4-95.0) | 93.4 (70.0-99.4) |
| Rwanda | 57.9 (49.8-65.4) | 80.1 (74.9-85.0) |  | 94.9 (92.2-96.7) | 98.2 (97.2-98.9) |  | 98.4 (96.7-99.3) | 99.4 (98.8-99.8) |
| Tanzania | 35.9 (10.6-68.6) | 73.6 (39.9-93.0) |  | 49.3 (38.3-60.7) | 85.2 (78.8-90.4) |  | 52.0 (31.8-71.7) | 86.0 (73.7-93.6) |
| Uganda | 24.3 (17.8-31.5) | 38.9 (30.2-48.1) |  | 79.5 (69.2-87.3) | 88.5 (82.3-93.0) |  | 90.4 (75.8-97.6) | 94.9 (87.0-98.7) |
| Zambia | 36.4 (28.5-44.6) | 80.8 (75.0-86.0) |  | 74.0 (66.9-79.9) | 95.5 (93.8-96.8) |  | 86.4 (77.4-92.7) | 97.9 (96.3-99.0) |
| Zimbabwe | 8.3 (4.2-13.4) | 25.9 (14.8-38.8) |  | 85.0 (80.8-88.3) | 95.7 (94.3-96.8) |  | 95.2 (92.0-97.3) | 98.7 (97.8-99.3) |
| **West Africa** |  |  |  |  |  |  |  |  |
| Benin | 54.1 (35.1-71.7) | 85.4 (73.9-92.5) |  | 65.7 (58.4-72.7) | 90.8 (87.4-93.3) |  | 60.4 (41.7-75.9) | 88.3 (79.0-94.3) |
| Burkina Faso | 22.5 (12.6-35.2) | 58.5 (41.9-73.2) |  | 80.5 (59.0-93.4) | 95.2 (88.1-98.6) |  | 88.5 (61.3-98.7) | 97.3 (89.0-99.7) |
| Cote d'Ivoire | 50.4 (41.9-58.5) | 85.8 (81.3-89.5) |  | 55.9 (43.7-66.6) | 88.2 (82.1-92.5) |  | 61.3 (32.8-82.4) | 89.6 (74.5-96.8) |
| The Gambia | 46.6 (39.6-53.9) | 74.4 (68.6-79.5) |  | 69.9 (64.5-75.3) | 88.6 (85.9-90.9) |  | 78.8 (64.9-88.6) | 92.4 (86.1-96.2) |
| Ghana | 37.0 (20.1-59.2) | 75.2 (59.0-89.0) |  | 72.0 (65.9-77.2) | 93.4 (91.6-95.1) |  | 84.8 (77.9-89.8) | 96.9 (95.3-98.0) |
| Guinea | 24.6 (12.1-40.1) | 68.7 (51.0-82.8) |  | 45.4 (34.4-56.3) | 85.4 (79.0-90.3) |  | 49.4 (25.2-71.2) | 86.4 (71.8-94.8) |
| Liberia | 12.4 (8.3-17.7) | 47.3 (36.0-58.7) |  | 56.6 (48.8-64.1) | 89.3 (86.0-92.2) |  | 69.7 (50.7-84.2) | 93.4 (87.0-97.0) |
| Mali | 14.3 (9.3-21.0) | 55.2 (42.9-67.0) |  | 33.4 (26.2-41.1) | 78.9 (72.4-83.7) |  | 35.9 (22.9-49.3) | 80.3 (69.7-88.1) |
| Niger | 18.9 (14.5-23.8) | 62.2 (54.2-69.7) |  | 26.9 (17.2-39.1) | 71.7 (59.7-82.7) |  | 35.2 (19.1-56.2) | 78.2 (62.3-90.4) |
| Nigeria | 1.9 (1.0-3.4) | 25.0 (15.1-37.1) |  | 22.7 (18.3-27.4) | 83.4 (79.5-86.9) |  | 62.9 (47.1-76.5) | 96.6 (93.9-98.3) |
| Senegal | 25.2 (16.5-34.9) | 56.5 (43.9-67.8) |  | 58.8 (53.3-64.5) | 84.9 (81.9-87.6) |  | 72.5 (63.5-80.0) | 91.1 (87.3-94.0) |
| Sierra Leone | 33.6 (27.3-39.8) | 65.0 (58.3-71.1) |  | 82.6 (78.8-85.8) | 94.6 (93.3-95.8) |  | 94.2 (91.8-96.0) | 98.4 (97.6-98.9) |
| Togo | 49.3 (42.3-56.3) | 80.5 (75.7-84.6) |  | 66.0 (58.6-73.6) | 89.2 (85.4-92.1) |  | 68.4 (48.8-84.0) | 89.9 (80.5-95.8) |

Note: CAF, Central African Republic; DRC, Democratic Republic of the Congo; Sao TP, Sao Tome and Principe

Supplementary Table S10: Coverage of age-appropriate DTP3 vaccine across the poorest and richest quintile in 41 LMICs in Asia and Sub-Saharan Africa, 2000-2030

| **Country** | **Predicted coverage (95% credible intervals) in year** | | | | | | | |
| --- | --- | --- | --- | --- | --- | --- | --- | --- |
|  | **2000** | |  | **2020** | |  | **2030** | |
|  | **Poorest** | **Richest** |  | **Poorest** | **Richest** |  | **Poorest** | **Richest** |
| **South Asia** |  |  |  |  |  |  |  |  |
| Bangladesh | 16.5 (13.0-20.6) | 37.1 (30.1-43.8) |  | 41.5 (35.3-48.5) | 67.9 (62.0-73.7) |  | 57.3 (47.0-66.9) | 79.9 (73.1-86.0) |
| India | 8.0 (5.0-12.9) | 29.1 (19.5-40.6) |  | 20.6 (14.0-27.9) | 54.9 (44.1-64.9) |  | 31.5 (16.6-48.9) | 67.5 (48.2-81.5) |
| Nepal | 9.9 (7.6-12.7) | 24.4 (19.1-30.1) |  | 35.4 (28.9-42.0) | 61.7 (54.2-68.4) |  | 52.9 (39.6-65.5) | 76.5 (65.1-84.8) |
| Pakistan | 2.5 (0.0-22.1) | 8.9 (0.1-61.4) |  | 17.9 (12.3-24.7) | 54.8 (44.7-65.2) |  | 48.7 (24.7-72.4) | 83.1 (66.5-93.8) |
| **Southeast Asia** |  |  |  |  |  |  |  |  |
| Cambodia | 4.8 (3.5-6.3) | 23.2 (17.9-28.9) |  | 57.3 (44.4-69.7) | 88.8 (82.6-93.4) |  | 84.3 (70.7-93.8) | 96.9 (93.4-98.9) |
| Laos | 2.6 (1.9-3.5) | 20.5 (15.6-26.1) |  | 15.3 (10.6-21.1) | 63.1 (53.6-72.3) |  | 32.3 (19.1-47.0) | 81.3 (70.0-89.8) |
| Myanmar | 9.5 (6.8-12.5) | 19.2 (13.8-25.8) |  | 20.7 (10.1-36.4) | 36.7 (19.8-56.6) |  | 32.8 (5.5-76.9) | 49.0 (11.7-87.5) |
| Timor-Leste | 11.4 (0.1-60.2) | 28.3 (0.7-88.0) |  | 27.0 (15.8-41.3) | 62.7 (47.3-77.1) |  | 52.2 (17.9-85.9) | 81.3 (52.6-96.6) |
| Vietnam | 21.9 (9.2-39.6) | 41.9 (20.8-64.8) |  | 16.6 (10.7-23.6) | 34.5 (23.5-46.6) |  | 19.5 (7.6-38.2) | 38.3 (18.1-63.8) |
| **Central Africa** |  |  |  |  |  |  |  |  |
| Angola | 2.2 (1.1-3.6) | 16.0 (9.0-24.6) |  | 18.5 (11.6-27.1) | 65.3 (51.7-76.8) |  | 40.2 (23.7-58.1) | 84.4 (72.0-92.6) |
| Cameroon | 10.3 (8.0-13.0) | 43.6 (36.8-50.9) |  | 33.3 (27.8-40.2) | 77.1 (71.7-81.9) |  | 42.0 (26.3-58.9) | 82.4 (70.1-90.6) |
| CAR | 4.7 (3.5-6.3) | 21.7 (16.7-27.2) |  | 8.5 (6.3-11.1) | 34.0 (26.9-40.9) |  | 19.0 (7.6-36.5) | 54.6 (31.3-76.6) |
| Chad | 2.8 (2.1-3.6) | 10.4 (7.8-13.6) |  | 4.5 (2.7-6.8) | 15.8 (10.1-22.5) |  | 5.9 (3.3-9.4) | 19.9 (12.4-29.4) |
| Congo | 24.1 (7.7-46.2) | 63.7 (36.4-84.6) |  | 24.5 (10.8-43.3) | 65.2 (43.2-82.8) |  | 36.5 (9.2-75.9) | 73.7 (35.1-95.4) |
| DRC | 1.8 (1.3-2.3) | 9.8 (7.2-12.9) |  | 9.9 (7.1-13.4) | 39.6 (31.9-47.8) |  | 5.4 (2.5-9.7) | 25.2 (14.0-39.2) |
| Sao TP | 17.6 (13.4-22.1) | 42.9 (35.4-51.0) |  | 73.7 (67.7-78.9) | 90.8 (87.9-93.0) |  | 91.6 (88.0-94.2) | 97.5 (96.1-98.4) |
| **East Africa** |  |  |  |  |  |  |  |  |
| Burundi | 6.5 (2.7-12.0) | 14.0 (6.3-24.5) |  | 70.5 (62.2-78.0) | 84.8 (78.4-89.5) |  | 85.8 (72.0-94.4) | 93.2 (85.1-97.6) |
| Comoros | 6.8 (4.7-9.4) | 23.9 (17.6-31.1) |  | 31.0 (11.9-61.7) | 63.4 (34.7-86.9) |  | 50.2 (13.5-89.7) | 77.5 (39.0-97.2) |
| Ethiopia | 2.8 (2.1-3.7) | 27.4 (21.8-33.6) |  | 16.1 (12.6-20.1) | 71.2 (64.8-77.0) |  | 41.2 (28.9-53.7) | 89.8 (83.9-94.0) |
| Kenya | 25.9 (20.3-32.1) | 63.4 (56.0-70.1) |  | 66.8 (50.6-81.0) | 90.7 (84.2-95.4) |  | 85.3 (62.1-97.0) | 96.5 (89.2-99.3) |
| Madagascar | 11.2 (8.3-14.6) | 40.4 (32.6-47.6) |  | 23.6 (17.5-30.9) | 62.2 (53.5-70.8) |  | 32.4 (21.4-45.6) | 71.4 (59.0-82.1) |
| Malawi | 11.1 (8.8-14.0) | 27.4 (22.2-33.1) |  | 47.0 (37.3-57.8) | 72.6 (64.2-80.7) |  | 66.1 (42.9-86.8) | 84.9 (69.3-95.2) |
| Mozambique | 5.0 (2.7-8.3) | 31.3 (20.0-45.0) |  | 18.4 (4.3-42.2) | 62.1 (29.4-86.7) |  | 33.5 (3.6-78.9) | 73.6 (26.2-96.9) |
| Rwanda | 37.7 (31.0-45.5) | 57.7 (49.9-65.0) |  | 82.6 (74.8-88.7) | 91.4 (86.8-94.6) |  | 92.1 (83.3-97.0) | 96.3 (91.9-98.6) |
| Tanzania | 16.9 (3.5-43.3) | 40.3 (12.4-73.6) |  | 44.0 (32.3-55.9) | 74.5 (64.4-83.3) |  | 60.7 (38.5-80.0) | 84.8 (70.9-93.7) |
| Uganda | 10.5 (7.3-14.5) | 24.6 (18.2-32.6) |  | 41.7 (29.1-56.6) | 66.3 (52.6-78.4) |  | 62.2 (31.9-88.3) | 80.9 (55.3-95.4) |
| Zambia | 5.2 (3.7-7.2) | 21.6 (16.1-27.8) |  | 38.0 (30.6-45.9) | 75.6 (69.5-81.4) |  | 67.0 (52.9-80.4) | 91.0 (85.3-95.5) |
| Zimbabwe | 1.2 (0.6-2.2) | 6.4 (3.2-11.7) |  | 54.0 (46.5-61.7) | 86.6 (83.0-89.8) |  | 86.1 (78.0-92.4) | 97.2 (95.2-98.5) |
| **West Africa** |  |  |  |  |  |  |  |  |
| Benin | 17.3 (9.0-29.6) | 44.3 (28.0-63.7) |  | 25.6 (19.3-32.6) | 57.3 (48.3-65.5) |  | 33.4 (17.9-51.9) | 65.1 (46.1-80.3) |
| Burkina Faso | 6.3 (3.0-11.9) | 15.0 (7.4-26.3) |  | 46.3 (20.9-73.7) | 68.1 (40.0-87.9) |  | 66.0 (22.3-94.7) | 81.4 (41.9-97.9) |
| Cote d'Ivoire | 13.9 (10.3-18.0) | 42.2 (33.9-50.0) |  | 18.2 (11.3-27.2) | 49.8 (37.2-62.9) |  | 21.0 (6.4-48.1) | 51.8 (23.3-80.7) |
| The Gambia | 8.7 (6.5-11.2) | 12.7 (9.7-16.1) |  | 34.4 (28.9-40.0) | 44.5 (38.2-51.0) |  | 58.6 (41.5-74.5) | 68.2 (52.4-81.5) |
| Ghana | 12.9 (4.8-27.5) | 35.0 (16.4-58.9) |  | 55.3 (48.2-62.5) | 82.5 (78.1-86.5) |  | 78.4 (69.4-85.3) | 93.2 (89.8-95.7) |
| Guinea | 8.4 (3.5-18.0) | 35.7 (19.1-57.7) |  | 8.9 (5.5-13.2) | 38.1 (27.2-50.0) |  | 10.1 (2.8-23.5) | 39.6 (15.0-66.9) |
| Liberia | 3.3 (2.1-5.1) | 17.1 (11.1-24.0) |  | 22.2 (17.0-27.7) | 63.0 (55.0-70.2) |  | 39.9 (21.4-61.7) | 78.8 (61.5-91.0) |
| Mali | 4.7 (2.7-7.5) | 19.7 (11.9-28.0) |  | 16.8 (12.7-21.8) | 50.0 (41.0-59.0) |  | 20.3 (12.1-30.6) | 55.2 (40.5-70.1) |
| Niger | 8.9 (6.5-11.7) | 29.6 (22.3-36.9) |  | 11.9 (6.5-18.3) | 36.2 (23.7-49.7) |  | 18.2 (7.9-32.1) | 47.7 (28.0-67.7) |
| Nigeria | 1.3 (0.7-2.0) | 13.6 (8.5-20.1) |  | 11.9 (9.4-15.0) | 62.4 (55.9-68.5) |  | 36.9 (24.1-50.4) | 87.3 (79.9-92.8) |
| Senegal | 8.7 (5.2-13.7) | 32.6 (22.0-45.1) |  | 26.7 (22.2-31.1) | 65.3 (59.8-70.1) |  | 38.2 (28.3-48.7) | 75.9 (67.5-83.5) |
| Sierra Leone | 11.3 (8.8-14.4) | 32.2 (26.4-38.4) |  | 31.7 (26.9-36.8) | 63.3 (57.5-68.9) |  | 56.8 (47.3-66.9) | 82.9 (76.1-88.3) |
| Togo | 9.1 (7.1-11.6) | 30.4 (24.7-36.2) |  | 59.5 (50.4-68.6) | 86.4 (81.0-90.8) |  | 97.6 (94.1-99.4) | 99.4 (98.5-99.9) |

Note: CAF, Central African Republic; DRC, Democratic Republic of the Congo; Sao TP, Sao Tome and Principe

Supplementary Table S11: Coverage of age-appropriate polio3 vaccine across the poorest and richest quintile in 41 LMICs in Asia and Sub-Saharan Africa, 2000-2030

| **Country** | **Predicted coverage (95% credible intervals) in year** | | | | | | | |
| --- | --- | --- | --- | --- | --- | --- | --- | --- |
|  | **2000** | |  | **2020** | |  | **2030** | |
|  | **Poorest** | **Richest** |  | **Poorest** | **Richest** |  | **Poorest** | **Richest** |
| **South Asia** |  |  |  |  |  |  |  |  |
| Bangladesh | 16.1 (12.7-20.3) | 36.8 (30.4-44.0) |  | 40.6 (33.1-48.4) | 67.3 (60.1-73.7) |  | 56.1 (44.4-66.9) | 79.3 (71.3-86.1) |
| India | 12.1 (7.1-19.1) | 35.6 (22.7-50.7) |  | 20.8 (13.2-28.9) | 51.4 (38.2-63.9) |  | 27.2 (12.9-44.4) | 59.1 (37.1-76.6) |
| Nepal | 21.2 (16.2-26.8) | 39.8 (31.8-47.9) |  | 39.6 (32.5-47.3) | 61.7 (54.4-68.8) |  | 50.5 (39.0-62.2) | 71.4 (61.1-79.8) |
| Pakistan | 2.0 (0.5-5.5) | 7.5 (2.1-18.4) |  | 27.2 (18.1-37.2) | 59.6 (47.7-71.5) |  | 62.1 (35.1-83.6) | 85.9 (68.0-95.3) |
| **Southeast Asia** |  |  |  |  |  |  |  |  |
| Cambodia | 5.8 (4.3-7.7) | 24.8 (18.4-31.7) |  | 62.1 (52.0-71.1) | 89.7 (85.0-93.3) |  | 89.2 (81.7-94.1) | 97.8 (96.0-98.9) |
| Laos | 2.7 (1.9-3.6) | 18.4 (13.5-24.5) |  | 17.9 (13.1-23.8) | 64.0 (54.6-72.4) |  | 38.0 (25.9-51.7) | 83.1 (74.0-89.8) |
| Myanmar | 10.5 (7.3-14.9) | 21.5 (14.9-29.7) |  | 20.5 (13.9-28.9) | 37.6 (26.2-48.7) |  | 27.8 (16.1-42.0) | 47.0 (30.2-63.0) |
| Timor-Leste | 4.7 (1.7-9.9) | 16.0 (6.2-30.8) |  | 24.8 (15.2-36.8) | 56.3 (40.5-70.7) |  | 46.4 (20.7-73.3) | 75.7 (50.1-92.3) |
| Vietnam | 22.2 (13.1-34.0) | 39.6 (26.5-54.5) |  | 23.1 (15.3-33.3) | 40.9 (28.1-54.7) |  | 24.3 (10.5-44.6) | 41.8 (20.3-65.8) |
| **Central Africa** |  |  |  |  |  |  |  |  |
| Angola | 3.6 (2.4-5.3) | 16.8 (11.2-23.6) |  | 17.8 (11.9-25.5) | 53.3 (40.8-64.8) |  | 33.9 (19.0-50.6) | 72.4 (54.5-85.0) |
| Cameroon | 12.5 (9.7-15.9) | 41.4 (34.1-49.5) |  | 42.2 (34.8-49.8) | 78.3 (72.9-83.2) |  | 62.0 (50.9-72.3) | 88.9 (84.0-92.8) |
| CAR | 5.0 (3.6-6.7) | 19.7 (14.4-25.6) |  | 13.3 (9.7-17.6) | 41.7 (32.9-51.3) |  | 21.1 (13.3-30.4) | 55.2 (41.9-68.4) |
| Chad | 4.0 (3.0-5.4) | 12.8 (9.2-17.2) |  | 7.3 (4.8-10.8) | 21.5 (14.8-29.8) |  | 9.9 (5.3-16.7) | 27.4 (15.9-41.5) |
| Congo | 20.3 (12.7-29.2) | 50.0 (36.6-62.3) |  | 23.1 (16.0-31.5) | 54.2 (43.0-65.6) |  | 24.9 (12.7-40.5) | 55.8 (36.9-74.0) |
| DRC | 2.8 (2.0-3.8) | 11.4 (8.1-15.1) |  | 39.0 (31.0-46.7) | 73.5 (65.8-79.7) |  | 73.6 (60.2-82.9) | 92.3 (87.3-95.5) |
| Sao TP | 15.8 (11.7-20.6) | 38.4 (29.8-46.7) |  | 72.8 (66.2-78.8) | 89.9 (86.2-92.9) |  | 91.0 (86.7-94.4) | 97.1 (95.4-98.3) |
| **East Africa** |  |  |  |  |  |  |  |  |
| Burundi | 17.4 (11.8-24.0) | 35.0 (25.2-46.0) |  | 69.8 (60.4-78.0) | 85.6 (79.6-90.6) |  | 88.2 (79.5-93.8) | 95.0 (91.3-97.6) |
| Comoros | 7.0 (4.7-9.8) | 23.2 (15.9-31.8) |  | 37.4 (25.2-50.5) | 69.9 (56.3-81.3) |  | 61.9 (41.1-79.4) | 86.0 (72.5-94.3) |
| Ethiopia | 4.8 (3.5-6.4) | 34.4 (27.6-42.8) |  | 15.0 (11.7-19.1) | 64.8 (57.1-71.7) |  | 24.9 (17.8-33.2) | 77.4 (69.0-84.3) |
| Kenya | 20.7 (15.4-26.2) | 52.1 (43.8-60.1) |  | 60.2 (48.6-70.6) | 86.2 (79.5-91.4) |  | 78.5 (62.5-89.3) | 93.7 (87.3-97.5) |
| Madagascar | 11.8 (8.7-15.2) | 38.5 (30.8-46.6) |  | 29.5 (21.9-36.9) | 65.9 (56.7-74.6) |  | 42.1 (28.6-55.7) | 76.8 (65.3-86.3) |
| Malawi | 10.9 (8.3-13.8) | 26.7 (21.3-33.0) |  | 41.4 (33.9-49.2) | 67.7 (60.2-74.7) |  | 62.6 (50.4-73.1) | 83.2 (75.6-89.3) |
| Mozambique | 5.7 (3.6-8.4) | 30.3 (21.3-40.1) |  | 12.4 (6.3-21.1) | 49.8 (32.3-65.6) |  | 18.5 (6.0-38.4) | 59.4 (30.7-81.8) |
| Rwanda | 37.7 (30.1-45.5) | 59.6 (51.0-67.5) |  | 82.1 (75.5-87.6) | 91.8 (88.2-94.6) |  | 92.5 (87.1-96.3) | 96.8 (94.1-98.5) |
| Tanzania | 19.0 (12.5-27.0) | 43.8 (31.2-56.5) |  | 41.9 (30.5-53.2) | 70.5 (60.5-79.4) |  | 55.6 (35.2-73.0) | 80.2 (65.2-90.3) |
| Uganda | 10.7 (7.8-14.3) | 25.5 (19.1-32.8) |  | 39.1 (30.4-48.6) | 64.5 (55.2-73.1) |  | 59.6 (44.9-72.3) | 80.5 (70.6-88.5) |
| Zambia | 5.8 (4.0-8.0) | 22.2 (16.1-29.0) |  | 35.9 (28.2-43.7) | 72.2 (64.8-79.3) |  | 62.6 (49.1-73.8) | 88.5 (82.7-93.3) |
| Zimbabwe | 0.0 (0.0-0.0) | 0.0 (0.0-0.1) |  | 79.1 (72.8-84.4) | 92.0 (89.3-94.3) |  | 99.8 (99.7-99.9) | 99.9 (99.8-99.9) |
| **West Africa** |  |  |  |  |  |  |  |  |
| Benin | 18.6 (12.2-26.7) | 47.5 (34.3-60.8) |  | 26.3 (19.8-33.7) | 58.6 (48.9-67.3) |  | 31.2 (18.1-46.5) | 63.7 (45.4-78.0) |
| Burkina Faso | 7.2 (4.7-10.2) | 17.1 (11.6-24.6) |  | 49.7 (31.8-67.6) | 71.9 (55.6-85.7) |  | 76.3 (51.2-92.2) | 89.1 (74.0-97.1) |
| Cote d'Ivoire | 16.0 (11.6-21.3) | 42.9 (34.2-52.0) |  | 28.8 (20.8-38.1) | 61.3 (50.6-71.3) |  | 37.0 (23.8-53.2) | 69.3 (55.0-81.7) |
| The Gambia | 9.6 (7.1-12.4) | 14.5 (10.8-18.9) |  | 31.8 (25.8-38.0) | 42.8 (35.3-50.4) |  | 49.3 (39.3-59.6) | 60.9 (50.5-70.9) |
| Ghana | 16.8 (12.6-21.5) | 47.4 (39.5-55.5) |  | 49.1 (41.1-57.4) | 81.1 (75.5-85.8) |  | 67.6 (56.1-78.2) | 90.2 (84.8-94.1) |
| Guinea | 9.6 (5.8-14.7) | 36.2 (25.4-49.3) |  | 13.4 (9.3-19.2) | 45.1 (33.8-56.4) |  | 16.2 (8.4-27.9) | 49.8 (31.9-67.8) |
| Liberia | 5.3 (3.1-8.1) | 21.3 (13.1-31.6) |  | 24.5 (17.8-31.1) | 61.2 (52.0-70.5) |  | 43.9 (29.0-58.6) | 78.9 (67.8-88.0) |
| Mali | 8.3 (6.1-11.0) | 30.1 (22.6-37.6) |  | 20.5 (15.7-26.0) | 54.8 (46.6-63.2) |  | 30.1 (20.4-41.5) | 66.7 (54.7-76.9) |
| Niger | 8.2 (5.9-11.2) | 25.4 (18.7-32.9) |  | 16.8 (10.6-25.5) | 43.1 (30.5-57.1) |  | 23.6 (11.5-40.1) | 52.7 (33.6-73.1) |
| Nigeria | 4.3 (3.2-5.9) | 26.9 (20.8-33.3) |  | 15.0 (11.4-19.0) | 58.8 (51.7-65.6) |  | 25.7 (17.6-35.3) | 73.4 (63.3-81.3) |
| Senegal | 10.6 (7.3-14.7) | 36.4 (28.2-45.7) |  | 27.7 (23.2-32.5) | 65.0 (59.3-70.3) |  | 40.8 (31.1-50.5) | 76.9 (69.3-83.3) |
| Sierra Leone | 10.8 (8.1-13.9) | 29.7 (23.4-35.7) |  | 29.0 (23.3-35.1) | 58.6 (51.3-65.5) |  | 43.0 (33.2-53.5) | 72.1 (63.3-80.1) |
| Togo | 10.3 (7.6-13.6) | 32.2 (25.6-39.7) |  | 47.4 (38.5-56.2) | 78.7 (72.3-84.2) |  | 71.3 (59.5-81.0) | 91.0 (85.8-94.6) |

Note: CAF, Central African Republic; DRC, Democratic Republic of the Congo; Sao TP, Sao Tome and Principe

Supplementary Table S12: Coverage of age-appropriate MCV across the poorest and richest quintile in 41 LMICs in Asia and Sub-Saharan Africa, 2000-2030

| **Country** | **Predicted coverage (95% credible intervals) in year** | | | | | | | |
| --- | --- | --- | --- | --- | --- | --- | --- | --- |
|  | **2000** | |  | **2020** | |  | **2030** | |
|  | **Poorest** | **Richest** |  | **Poorest** | **Richest** |  | **Poorest** | **Richest** |
| **South Asia** |  |  |  |  |  |  |  |  |
| Bangladesh | 30.9 (25.4-37.1) | 52.4 (44.7-59.4) |  | 57.1 (49.8-64.6) | 76.7 (70.8-81.7) |  | 69.6 (59.5-78.3) | 84.9 (78.2-89.9) |
| India | 9.3 (5.4-14.5) | 31.6 (21.2-44.8) |  | 29.9 (21.3-40.0) | 65.6 (54.1-75.6) |  | 46.6 (28.6-64.9) | 79.1 (65.2-89.3) |
| Nepal | 10.3 (7.8-13.4) | 29.2 (23.0-36.0) |  | 54.0 (46.6-61.5) | 80.7 (75.6-85.0) |  | 78.9 (70.3-85.3) | 93.0 (89.7-95.5) |
| Pakistan | 1.7 (0.6-3.9) | 10.4 (3.8-22.8) |  | 19.6 (12.7-27.0) | 61.9 (49.6-71.7) |  | 48.7 (26.3-70.4) | 85.6 (71.2-94.1) |
| **Southeast Asia** |  |  |  |  |  |  |  |  |
| Cambodia | 9.4 (7.1-12.5) | 33.8 (26.5-42.0) |  | 65.7 (56.4-75.2) | 90.3 (85.8-93.8) |  | 88.9 (81.7-94.2) | 97.5 (95.5-98.8) |
| Laos | 3.6 (2.5-4.8) | 17.7 (13.1-23.3) |  | 28.5 (21.3-36.2) | 69.6 (60.9-77.1) |  | 56.5 (42.6-69.4) | 88.1 (81.1-92.8) |
| Myanmar | 20.9 (14.7-27.9) | 47.4 (37.9-58.1) |  | 26.7 (19.0-35.6) | 55.4 (44.4-65.8) |  | 30.1 (19.0-43.4) | 59.2 (43.9-72.2) |
| Timor-Leste | 9.5 (4.4-18.3) | 27.0 (14.7-44.6) |  | 32.5 (22.0-44.0) | 63.0 (48.7-74.5) |  | 51.0 (26.5-73.4) | 77.8 (56.0-90.9) |
| Vietnam | 31.5 (20.8-43.1) | 58.6 (45.8-71.7) |  | 39.0 (27.8-51.8) | 66.3 (53.7-77.2) |  | 43.2 (23.0-64.5) | 69.3 (47.9-85.2) |
| **Central Africa** |  |  |  |  |  |  |  |  |
| Angola | 3.3 (2.2-4.8) | 20.9 (14.1-28.6) |  | 19.0 (12.9-26.0) | 64.1 (52.7-74.8) |  | 37.9 (24.3-52.7) | 82.0 (70.6-90.0) |
| Cameroon | 16.1 (12.6-20.2) | 55.2 (48.0-62.2) |  | 30.7 (24.1-37.2) | 74.0 (67.8-79.6) |  | 40.2 (29.7-50.7) | 81.1 (73.8-87.2) |
| CAR | 6.2 (4.4-8.2) | 28.8 (22.4-35.8) |  | 8.4 (6.1-11.1) | 36.2 (27.9-44.3) |  | 10.0 (6.5-14.9) | 40.4 (28.5-52.4) |
| Chad | 2.5 (1.8-3.3) | 9.8 (7.2-13.1) |  | 8.0 (5.5-11.3) | 26.9 (19.2-35.4) |  | 13.9 (8.2-22.3) | 40.3 (27.5-54.9) |
| Congo | 16.5 (11.0-23.3) | 51.8 (40.6-64.0) |  | 24.7 (17.1-33.1) | 64.1 (53.0-73.9) |  | 29.9 (16.5-45.9) | 69.2 (51.5-82.9) |
| DRC | 3.0 (2.2-4.2) | 14.2 (10.6-18.6) |  | 20.9 (16.4-26.5) | 58.0 (49.8-66.2) |  | 43.1 (31.7-55.3) | 79.6 (70.4-86.9) |
| Sao TP | 26.5 (20.0-33.2) | 45.7 (37.4-54.7) |  | 62.5 (54.6-69.4) | 79.5 (73.1-84.6) |  | 78.1 (69.1-85.2) | 89.2 (83.6-93.2) |
| **East Africa** |  |  |  |  |  |  |  |  |
| Burundi | 31.0 (21.8-41.1) | 48.4 (36.5-60.1) |  | 73.0 (63.4-80.5) | 85.0 (78.6-90.1) |  | 86.6 (76.5-93.2) | 93.1 (87.9-96.7) |
| Comoros | 10.7 (7.6-14.6) | 33.7 (24.8-43.6) |  | 28.6 (18.3-39.5) | 62.7 (49-74.0) |  | 42.3 (23.8-60.4) | 74.9 (58.1-87.3) |
| Ethiopia | 3.1 (2.3-4.2) | 21.9 (16.4-27.5) |  | 13.6 (10.4-17.3) | 57.5 (49.3-65.3) |  | 25.9 (18.8-35.0) | 74.8 (65.6-82.3) |
| Kenya | 23.4 (18.3-29.2) | 56.6 (48.1-64.5) |  | 42.1 (31.5-53.2) | 75.5 (66.0-83.2) |  | 52.7 (34.3-69.3) | 82.1 (69.1-90.8) |
| Madagascar | 15.2 (11.6-19.5) | 52.2 (44.0-60.7) |  | 25.8 (19.4-33.6) | 67.8 (59.3-75.4) |  | 32.7 (22.0-44.9) | 74.3 (63.6-83.4) |
| Malawi | 20.5 (16.2-25.5) | 34.5 (28.6-41.2) |  | 45.5 (37.4-53.3) | 63.1 (55.2-70.4) |  | 59.9 (47.5-71.0) | 75.2 (65.0-83.1) |
| Mozambique | 21.2 (14.7-28.9) | 63.4 (52.8-73.4) |  | 32.8 (20.2-47.0) | 75.4 (62.7-85.8) |  | 39.8 (18.6-63.6) | 79.7 (60.0-92.2) |
| Rwanda | 44.1 (36.1-52.6) | 62.3 (54.0-70.4) |  | 73.0 (60.3-83.3) | 84.9 (76.4-91.3) |  | 82.8 (67.3-92.8) | 90.9 (81.1-96.4) |
| Tanzania | 25.4 (17.5-34.3) | 55.2 (43.8-66.0) |  | 40.1 (30.2-51.2) | 70.8 (60.7-79.5) |  | 48.3 (31.3-65.7) | 76.8 (63.2-87.8) |
| Uganda | 14.0 (10.1-18.2) | 27.8 (20.9-35.5) |  | 42.1 (33.5-51.7) | 63.1 (53.5-72.2) |  | 60.3 (46.8-73.5) | 78.0 (66.5-86.6) |
| Zambia | 23.1 (17.5-29.6) | 52.1 (43.0-60.7) |  | 45.4 (37.4-53.8) | 75.1 (67.8-81.0) |  | 57.9 (46.0-69.6) | 83.2 (74.7-89.2) |
| Zimbabwe | 18.0 (11.8-27.0) | 44.3 (31.7-56.9) |  | 55.0 (47.1-62.8) | 81.7 (75.9-86.5) |  | 74.2 (61.6-84.2) | 91.2 (84.9-95.2) |
| **West Africa** |  |  |  |  |  |  |  |  |
| Benin | 19.6 (13.2-27.4) | 49.6 (37.6-61.5) |  | 35.7 (27.5-44.2) | 69.2 (61.2-76.2) |  | 45.5 (30.4-61.1) | 76.8 (64.5-86.1) |
| Burkina Faso | 15.3 (10.6-20.9) | 36.2 (26.5-46.8) |  | 71.0 (53.7-83.5) | 88.3 (79.3-94.3) |  | 89.2 (73.6-96.7) | 96.2 (90.2-99.0) |
| Cote d’Ivoire | 22.4 (17.2-28.4) | 53.1 (44.1-62.1) |  | 26.4 (18.7-35.4) | 58.3 (47.5-69.0) |  | 28.7 (17.7-42.5) | 60.6 (45.8-74.9) |
| The Gambia | 35.7 (29.0-42.6) | 47.3 (40.0-55.1) |  | 54.4 (47.4-60.8) | 65.7 (59.3-72.3) |  | 63.5 (53.9-71.7) | 73.6 (66.0-81.1) |
| Ghana | 27.1 (21.5-33.6) | 56.2 (47.7-63.9) |  | 50.2 (42.4-57.5) | 77.7 (71.9-82.9) |  | 62.3 (50.6-72.7) | 85.0 (78.1-90.4) |
| Guinea | 11.9 (7.6-16.9) | 38.3 (27.3-49.0) |  | 12.1 (8.2-16.8) | 38.8 (29.0-49.7) |  | 12.5 (6.5-21.1) | 39.2 (23.8-56.3) |
| Liberia | 6.3 (3.9-9.9) | 25.3 (16.3-35.5) |  | 28.2 (21.2-35.7) | 66.4 (57.4-74.3) |  | 48.7 (34.9-62.9) | 82.5 (73.0-89.7) |
| Mali | 10.2 (7.6-13.1) | 34.7 (27.4-41.7) |  | 24.8 (19.3-31.0) | 60.7 (52.7-68.0) |  | 36.0 (26.0-47.0) | 72.3 (62.1-81.3) |
| Niger | 9.0 (6.5-12.1) | 34.1 (26.5-42.6) |  | 19.7 (12.5-28.8) | 55.6 (42.7-68.9) |  | 27.9 (14.8-45.2) | 65.7 (47.4-81.9) |
| Nigeria | 2.6 (1.9-3.5) | 23.7 (18.2-29.3) |  | 11.9 (9.4-14.9) | 61.2 (53.7-68.0) |  | 23.4 (16.9-31.6) | 77.8 (68.8-84.9) |
| Senegal | 17.2 (12.4-22.8) | 42.4 (33.3-52.3) |  | 42.0 (36.8-47.5) | 72.0 (67.1-76.7) |  | 57.4 (47.9-67.0) | 82.6 (76.3-88.2) |
| Sierra Leone | 11.5 (8.8-14.6) | 26.5 (21.4-32.4) |  | 39.1 (33.2-45.6) | 64.0 (57.2-70.4) |  | 58.7 (49.4-68.2) | 79.7 (72.7-86.0) |
| Togo | 14.9 (11.3-19.2) | 35.4 (28.4-42.8) |  | 42.3 (34.7-51.3) | 69.7 (62.3-76.4) |  | 59.9 (47.9-71.8) | 82.3 (74.4-88.6) |

Note: CAF, Central African Republic; DRC, Democratic Republic of the Congo; Sao TP, Sao Tome and Principe

Supplementary Table S13: Projected coverage of age-appropriate BCG vaccine in 2030 with different scenarios

| **Country** | **Reference scenario** | **DAH reduction** | | **SHW reduction** | |
| --- | --- | --- | --- | --- | --- |
|  | **Coverage (95% CrI)** | **Coverage (95% CrI)** | **Coverage changes^1^** | **Coverage (95% CrI)** | **Coverage changes^2^** |
| **South Asia** |  |  |  |  |  |
| Bangladesh | 49.8 (39.6-60.5) | 48.9 (37.8-60.1) | 0.90 | 50.2 (38.0-62.3) | -0.40 |
| India | 85.2 (73.0-92.1) | 84.5 (73.6-92.5) | 0.70 | 85.0 (73.7-92.7) | 0.20 |
| Nepal | 76.4 (65.3-84.8) | 75.5 (63.3-83.8) | 0.90 | 76.1 (61.0-88.0) | 0.30 |
| Pakistan | 80.7 (63.4-91.9) | 80.2 (63.1-92.2) | 0.50 | 80.8 (63.1-92.6) | -0.10 |
| **Southeast Asia** |  |  |  |  |  |
| Cambodia | 97.9 (95.7-99.1) | 97.6 (94.6-99.2) | 0.30 | 97.9 (95.4-99.2) | 0.00 |
| Laos | 86.8 (78.9-92.5) | 86.3 (75.8-93.1) | 0.50 | 86.5 (78.0-92.6) | 0.30 |
| Myanmar | 63.4 (29.4-88.7) | 61.8 (26.3-88.3) | 1.60 | 64.4 (32.2-88.8) | -1.00 |
| Timor-Leste | 79.0 (52.6-93.7) | 79.7 (54.5-94.1) | -0.70 | 79.5 (52.7-94.0) | -0.50 |
| Vietnam | 80.3 (63.4-91.6) | 80.3 (62.9-91.8) | 0.00 | 80.6 (65.1-91.5) | -0.30 |
| **Central Africa** |  |  |  |  |  |
| Angola | 57.4 (41.0-72.5) | 56.9 (39.5-73.0) | 0.50 | 58.8 (42.3-73.7) | -1.40 |
| Cameroon | 72.0 (55.6-83.8) | 71.0 (55.1-83.4) | 1.00 | 71.8 (55.7-83.8) | 0.20 |
| CAF | 69.9 (49.8-85.2) | 71.1 (50.9-86.8) | -1.20 | 70.5 (49.8-84.6) | -0.60 |
| Chad | 21.6 (12.6-33.1) | 23.3 (14.1-34.8) | -1.70 | 21.7 (11.6-34.7) | -0.10 |
| Congo | 81.2 (56.0-94.8) | 81.3 (52.9-95.1) | -0.10 | 81.7 (58.4-94.7) | -0.50 |
| DRC | 19.5 (9.7-32.5) | 16.1 (7.2-29.2) | 3.40 | 20.3 (9.7-34.2) | -0.80 |
| STP | 98.1 (97.1-98.8) | 98.1 (97.2-98.8) | 0.00 | 98.1 (97.1-98.8) | 0.00 |
| **East Africa** |  |  |  |  |  |
| Burundi | 97.0 (93.9-98.7) | 96.7 (92.8-98.8) | 0.30 | 96.9 (93.0-98.9) | 0.10 |
| Comoros | 80.8 (51.1-95.8) | 79.2 (42.7-96.4) | 1.60 | 80.5 (47.4-96.1) | 0.30 |
| Ethiopia | 39.5 (26.8-53.7) | 39.9 (27.2-54.4) | -0.40 | 39.4 (23.7-58.2) | 0.10 |
| Kenya | 85.2 (66.3-96.2) | 86.1 (66.3-96.2) | -0.90 | 86.4 (68.3-95.6) | -1.20 |
| Madagascar | 60.7 (47.2-73.9) | 60.4 (44.8-73.3) | 0.30 | 61.4 (46.4-73.6) | -0.70 |
| Malawi | 92.6 (84.7-96.9) | 92.0 (83.9-96.7) | 0.60 | 91.8 (82.6-96.3) | 0.80 |
| Mozambique | 79.8 (41.4-97.4) | 79.5 (42.2-97.4) | 0.30 | 79.3 (40.0-97.3) | 0.50 |
| Rwanda | 98.2 (96.0-99.3) | 98.2 (95.9-99.3) | 0.00 | 98.2 (96.1-99.3) | 0.00 |
| Tanzania | 64.4 (43.1-81.4) | 63.9 (41.4-81.7) | 0.50 | 64.1 (42.6-82.3) | 0.30 |
| Uganda | 90.1 (76.7-96.7) | 90.0 (75.8-96.9) | 0.10 | 90.0 (76.4-96.9) | 0.10 |
| Zambia | 90.0 (82.8-94.5) | 90.3 (84.0-94.7) | -0.30 | 90.4 (83.2-95.1) | -0.40 |
| Zimbabwe | 96.7 (94.6-98.2) | 96.5 (93.8-98.2) | 0.20 | 96.8 (94.4-98.3) | -0.10 |
| **West Africa** |  |  |  |  |  |
| Benin | 74.2 (59.7-86.2) | 73.1 (56.4-85.4) | 1.10 | 75.2 (60.3-85.5) | -1.00 |
| Burkina Faso | 90.0 (66.6-98.2) | 90.1 (65.9-98.7) | -0.10 | 90.8 (68.9-98.6) | -0.80 |
| Cote d'Ivoire | 70.8 (46.5-87.9) | 69.8 (43.6-87.2) | 1.00 | 71.0 (48.4-87.4) | -0.20 |
| The Gambia | 81.1 (70.7-89.8) | 81.6 (70.8-89.6) | -0.50 | 81.5 (69.9-89.9) | -0.40 |
| Ghana | 89.0 (83.5-93.1) | 88.9 (83.4-93.0) | 0.10 | 88.8 (82.1-93.6) | 0.20 |
| Guinea | 67.9 (44.5-84.6) | 66.6 (42.0-84.1) | 1.30 | 67.3 (45.2-84.7) | 0.60 |
| Liberia | 82.8 (71.2-91.3) | 82.1 (69.0-91.1) | 0.70 | 82.1 (68.8-91.1) | 0.70 |
| Mali | 52.4 (37.2-66.4) | 52.6 (37.9-67.9) | -0.20 | 52.9 (38.1-68.1) | -0.50 |
| Niger | 52.7 (31.4-73.3) | 51.7 (31.6-71.2) | 1.00 | 53.5 (30.8-73.9) | -0.80 |
| Nigeria | 80.1 (68.0-88.5) | 80.8 (68.8-89.6) | -0.70 | 80.6 (68.9-89.7) | -0.50 |
| Senegal | 80.4 (72.5-86.4) | 80.1 (72.0-86.3) | 0.30 | 80.5 (70.5-88.2) | -0.10 |
| Sierra Leone | 94.7 (92.4-96.6) | 94.7 (91.9-96.6) | 0.00 | 94.7 (92.1-96.7) | 0.00 |
| Togo | 78.7 (64.0-89.5) | 78.2 (60.1-89.7) | 0.50 | 79.4 (64.1-89.9) | -0.70 |

Note: ^1^Change in the coverage was estimated as the absolute change in the coverage of age-appropriate BCG from reference scenario to the DAH reduction; ^2^Change in the coverage was estimated as the absolute change in the coverage of age-appropriate BCG from reference scenario to the SHW reduction; DAH, development assistance for health; SHW, Skilled health workforce; CrI, credible interval; CAF, Central African Republic; DRC, Democratic Republic of the Congo; Sao TP, Sao Tome and Principe

Supplementary Table S14: Projected coverage of age-appropriate DTP3 vaccine in 2030 with different scenarios

| **Country** | **Reference scenario** | **DAH reduction** | | **SHW reduction** | |
| --- | --- | --- | --- | --- | --- |
|  | **Coverage (95% CrI)** | **Coverage (95% CrI)** | **Coverage changes^1^** | **Coverage (95% CrI)** | **Coverage changes^2^** |
| **South Asia** |  |  |  |  |  |
| Bangladesh | 63.8 (54.7-72.5) | 63.0 (53.7-72.0) | 0.80 | 63.6 (51.9-74.7) | 0.20 |
| India | 39.1 (23.8-57.4) | 39.1 (22.7-56.5) | 0.00 | 39.5 (22.9-58.3) | -0.40 |
| Nepal | 61.8 (49.7-73.9) | 60.1 (45.3-73.4) | 1.70 | 59.0 (38.9-76.7) | 2.80 |
| Pakistan | 66.2 (42.1-85.4) | 65.1 (41.5-84.5) | 1.10 | 65.6 (40.7-84.4) | 0.60 |
| **Southeast Asia** |  |  |  |  |  |
| Cambodia | 92.1 (84.5-96.7) | 91.0 (80.4-96.6) | 1.10 | 92.0 (84.5-96.8) | 0.10 |
| Laos | 56.6 (42.4-71.5) | 56.5 (38.7-73.1) | 0.10 | 56.3 (40.0-70.9) | 0.30 |
| Myanmar | 35.0 (6.5-75.5) | 41.4 (9.9-83.6) | -6.40 | 38.2 (7.4-78.9) | -3.20 |
| Timor-Leste | 70.0 (35.4-91.5) | 70.9 (37.1-93.1) | -0.90 | 70.2 (39.0-91.8) | -0.20 |
| Vietnam | 29.1 (12.8-49.8) | 28.9 (11.9-51.5) | 0.20 | 28.5 (13.2-49.7) | 0.60 |
| **Central Africa** |  |  |  |  |  |
| Angola | 47.0 (30.6-64.2) | 46.4 (28.7-63.6) | 0.60 | 45.9 (28.9-63.2) | 1.10 |
| Cameroon | 64.2 (46.9-77.8) | 64.4 (47.1-78.8) | -0.20 | 65.1 (49.2-79.4) | -0.90 |
| CAF | 34.7 (15.4-57.2) | 39.6 (17.5-64.6) | -4.90 | 35.9 (16.4-58.9) | -1.20 |
| Chad | 10.5 (6.3-16.2) | 10.6 (6.4-16.2) | -0.10 | 9.6 (3.9-16.5) | 0.90 |
| Congo | 52.7 (19.7-85.7) | 52.8 (18.8-84.2) | -0.10 | 51.5 (19.3-82.7) | 1.20 |
| DRC | 6.9 (3.4-12.1) | 5.7 (2.5-10.5) | 1.20 | 7.2 (3.4-13.8) | -0.30 |
| STP | 94.3 (91.9-96.3) | 94.3 (91.7-96.3) | 0.00 | 94.2 (91.6-96.3) | 0.10 |
| **East Africa** |  |  |  |  |  |
| Burundi | 88.6 (77.1-95.2) | 87.0 (72.6-95.4) | 1.60 | 87.2 (72.8-95.4) | 1.40 |
| Comoros | 62.8 (22.8-92.1) | 63.6 (20.3-94.0) | -0.80 | 61.5 (21.3-90.6) | 1.30 |
| Ethiopia | 54.4 (40.6-68.1) | 54.1 (40.6-66.0) | 0.30 | 54.2 (33.0-74.4) | 0.20 |
| Kenya | 84.5 (61.6-96.0) | 83.2 (58.5-96.0) | 1.30 | 81.2 (52.6-95.0) | 3.30 |
| Madagascar | 46.3 (33.3-59.5) | 45.3 (31.5-59.3) | 1.00 | 46.1 (32.2-59.5) | 0.20 |
| Malawi | 73.6 (52.7-88.0) | 73.6 (52.6-88.6) | 0.00 | 72.6 (50.0-89.4) | 1.00 |
| Mozambique | 54.9 (15.3-91.0) | 52.7 (10.2-89.5) | 2.20 | 50.7 (11.8-90.5) | 4.20 |
| Rwanda | 92.9 (85.4-97.1) | 92.8 (85.0-97.3) | 0.10 | 92.6 (84.6-96.9) | 0.30 |
| Tanzania | 72.0 (52.6-86.2) | 71.9 (52.6-86.5) | 0.10 | 72.3 (53.4-86.7) | -0.30 |
| Uganda | 65.3 (34.6-87.0) | 65.9 (38.3-87.1) | -0.60 | 64.9 (36.4-88.1) | 0.40 |
| Zambia | 76.0 (63.7-85.7) | 76.3 (63.0-86.6) | -0.30 | 75.0 (62.4-85.7) | 1.00 |
| Zimbabwe | 92.0 (87.0-95.6) | 91.1 (85.6-95.1) | 0.90 | 91.5 (86.1-95.3) | 0.50 |
| **West Africa** |  |  |  |  |  |
| Benin | 45.2 (28.1-62.6) | 44.7 (25.1-65.5) | 0.50 | 43.9 (27.1-60.8) | 1.30 |
| Burkina Faso | 69.1 (29.8-94.2) | 68.9 (26.4-94.5) | 0.20 | 66.7 (22.1-94.9) | 2.40 |
| Cote d'Ivoire | 27.7 (9.4-53.5) | 28.7 (8.8-57.7) | -1.00 | 29.8 (9.4-57.7) | -2.10 |
| The Gambia | 62.3 (44.5-76.7) | 60.9 (45.4-75.8) | 1.40 | 61.4 (44.0-78.0) | 0.90 |
| Ghana | 83.7 (76.3-89.1) | 83.4 (76.5-89.2) | 0.30 | 82.4 (73.5-89.6) | 1.30 |
| Guinea | 21.6 (7.7-42.8) | 22.2 (7.4-44.4) | -0.60 | 21.5 (7.6-40.4) | 0.10 |
| Liberia | 56.3 (34.8-75.7) | 57.0 (35.4-75.9) | -0.70 | 56.7 (35.6-76.7) | -0.40 |
| Mali | 30.1 (19.3-43.6) | 30.0 (19.2-42.5) | 0.10 | 29.1 (17.9-43.1) | 1.00 |
| Niger | 30.0 (15.5-48.6) | 28.7 (14.6-46.3) | 1.30 | 29.7 (14.4-49.3) | 0.30 |
| Nigeria | 64.2 (49.9-76.5) | 64.5 (49.0-77.1) | -0.30 | 64.4 (50.1-77.3) | -0.20 |
| Senegal | 51.2 (40.8-61.7) | 50.6 (39.1-61.2) | 0.60 | 50.9 (35.5-65.8) | 0.30 |
| Sierra Leone | 58.8 (48.9-68.5) | 60.0 (49.6-70.0) | -1.20 | 58.9 (48.6-68.4) | -0.10 |
| Togo | 97.6 (93.7-99.4) | 98.1 (94.8-99.5) | -0.50 | 97.7 (93.7-99.4) | -0.10 |

Note: ^1^Change in the coverage was estimated as the absolute change in the coverage of age-appropriate BCG from reference scenario to the DAH reduction; ^2^Change in the coverage was estimated as the absolute change in the coverage of age-appropriate BCG from reference scenario to the SHW reduction; DAH, development assistance for health; SHW, Skilled health workforce; CrI, credible interval; CAF, Central African Republic; DRC, Democratic Republic of the Congo; Sao TP, Sao Tome and Principe

Supplementary Table S15: Projected coverage of age-appropriate polio3 vaccine in 2030 with different scenarios

| **Country** | **Reference scenario** | **DAH reduction** | | **SHW reduction** | |
| --- | --- | --- | --- | --- | --- |
|  | **Coverage (95% CrI)** | **Coverage (95% CrI)** | **Coverage changes^1^** | **Coverage (95% CrI)** | **Coverage changes^2^** |
| **South Asia** |  |  |  |  |  |
| Bangladesh | 62.4 (53.6-70.1) | 62.3 (53.1-70.6) | 0.10 | 63.1 (52.6-72.6) | -0.70 |
| India | 34.0 (21.2-48.8) | 34.4 (21.2-50.4) | -0.40 | 34.6 (21.5-51.7) | -0.60 |
| Nepal | 54.4 (43.2-65.5) | 54.4 (43.4-64.7) | 0.00 | 55.7 (39.3-70.6) | -1.30 |
| Pakistan | 68.2 (49.2-83.6) | 68.4 (48.4-84.1) | -0.20 | 69.0 (50.2-83.9) | -0.80 |
| **Southeast Asia** |  |  |  |  |  |
| Cambodia | 91.0 (84.3-95.6) | 90.4 (82.2-95.5) | 0.60 | 91.0 (84.6-95.3) | 0.00 |
| Laos | 60.8 (48.8-72.4) | 61.4 (46.7-74.6) | -0.60 | 61.9 (49.8-73.0) | -1.10 |
| Myanmar | 37.1 (14.3-63.0) | 37.8 (14.0-69.4) | -0.70 | 38.0 (14.0-64.8) | -0.90 |
| Timor-Leste | 61.5 (34.3-84.2) | 61.0 (34.5-84.2) | 0.50 | 63.2 (37.1-84.2) | -1.70 |
| Vietnam | 37.8 (21.2-55.9) | 37.4 (21.1-55.4) | 0.40 | 37.5 (20.4-56.4) | 0.30 |
| **Central Africa** |  |  |  |  |  |
| Angola | 39.8 (26.0-54.8) | 40.2 (26.6-54.1) | -0.40 | 41.0 (28.0-55.7) | -1.20 |
| Cameroon | 63.7 (48.1-76.4) | 63.1 (47.5-76.7) | 0.60 | 63.7 (49.4-77.1) | 0.00 |
| CAF | 42.8 (26.5-62.0) | 43.4 (25.7-62.2) | -0.60 | 42.9 (25.9-60.9) | -0.10 |
| Chad | 16.9 (10.7-24.6) | 17.4 (11.7-24.6) | -0.50 | 16.5 (9.2-25.8) | 0.40 |
| Congo | 46.6 (19.3-76.1) | 47.5 (21.0-73.9) | -0.90 | 46.8 (20.1-75.0) | -0.20 |
| DRC | 21.9 (12.4-34.7) | 19.6 (10.8-30.9) | 2.30 | 22.9 (12.4-36.2) | -1.00 |
| STP | 94.0 (91.6-95.9) | 94.0 (91.4-95.9) | 0.00 | 94.0 (91.5-96.0) | 0.00 |
| **East Africa** |  |  |  |  |  |
| Burundi | 85.5 (75.3-92.4) | 85.2 (73.2-92.5) | 0.30 | 85.6 (73.7-93.5) | -0.10 |
| Comoros | 64.1 (33.4-88.9) | 63.7 (29.4-89.2) | 0.40 | 65.9 (34.2-89.4) | -1.80 |
| Ethiopia | 48.7 (36.2-61.9) | 47.3 (36.0-58.4) | 1.40 | 49.2 (32.2-65.4) | -0.50 |
| Kenya | 80.4 (62.1-92.9) | 81.2 (63.0-93.0) | -0.80 | 81.5 (64.6-92.4) | -1.10 |
| Madagascar | 52.9 (40.6-65.1) | 52.4 (38.1-65.2) | 0.50 | 53.1 (40.5-64.9) | -0.20 |
| Malawi | 69.3 (52.4-83.3) | 69.5 (52.1-84.0) | -0.20 | 69.0 (51.2-82.0) | 0.30 |
| Mozambique | 56.3 (17.2-88.2) | 57.1 (19.1-89.2) | -0.80 | 56.0 (17.2-88.6) | 0.30 |
| Rwanda | 91.9 (85.2-96.4) | 92.2 (85.5-96.3) | -0.30 | 92.0 (85.7-96.2) | -0.10 |
| Tanzania | 68.0 (49.7-82.7) | 68.0 (51.1-82.8) | 0.00 | 69.0 (50.6-84.0) | -1.00 |
| Uganda | 58.7 (36.6-78.7) | 59.4 (36.7-78.9) | -0.70 | 58.9 (36.3-79.1) | -0.20 |
| Zambia | 71.2 (59.9-80.3) | 71.3 (60.1-80.6) | -0.10 | 71.4 (59.2-81.8) | -0.20 |
| Zimbabwe | 89.8 (84.3-93.8) | 89.1 (83.0-94.0) | 0.70 | 89.6 (84.1-93.7) | 0.20 |
| **West Africa** |  |  |  |  |  |
| Benin | 44.0 (31.1-58.4) | 44.4 (30.7-58.9) | -0.40 | 44.7 (30.3-59.0) | -0.70 |
| Burkina Faso | 70.4 (40.0-90.5) | 71.2 (43.4-90.6) | -0.80 | 69.9 (38.6-90.2) | 0.50 |
| Cote d'Ivoire | 39.2 (20.9-60.0) | 40.5 (22.7-62.5) | -1.30 | 40.8 (21.8-61.9) | -1.60 |
| The Gambia | 55.4 (42.1-69.3) | 55.7 (42.8-68.4) | -0.30 | 56.2 (42.5-69.3) | -0.80 |
| Ghana | 74.2 (65.5-81.9) | 74.1 (65.5-81.5) | 0.10 | 74.3 (64.5-82.1) | -0.10 |
| Guinea | 33.1 (17.4-51.8) | 34.7 (17.0-54.9) | -1.60 | 33.9 (17.6-52.6) | -0.80 |
| Liberia | 50.8 (34.2-66.3) | 51.3 (33.8-67.4) | -0.50 | 51.2 (34.5-66.2) | -0.40 |
| Mali | 26.5 (17.8-36.8) | 27.1 (17.9-38.6) | -0.60 | 27.0 (18.4-36.6) | -0.50 |
| Niger | 33.6 (18.8-50.7) | 32.3 (18.3-49.4) | 1.30 | 33.4 (17.8-53.2) | 0.20 |
| Nigeria | 63.0 (50.7-75.3) | 63.3 (50.1-74.8) | -0.30 | 63.4 (50.7-75.5) | -0.40 |
| Senegal | 50.4 (40.4-59.7) | 50.0 (40.1-60.3) | 0.40 | 50.7 (38.7-63.2) | -0.30 |
| Sierra Leone | 49.5 (40.7-58.1) | 50.1 (41.0-59.9) | -0.60 | 49.6 (40.7-58.2) | -0.10 |
| Togo | 86.6 (76.2-93.9) | 86.9 (77.2-94.6) | -0.30 | 86.8 (76.0-94.2) | -0.20 |

Note: ^1^Change in the coverage was estimated as the absolute change in the coverage of age-appropriate BCG from reference scenario to the DAH reduction; ^2^Change in the coverage was estimated as the absolute change in the coverage of age-appropriate BCG from reference scenario to the SHW reduction; DAH, development assistance for health; SHW, Skilled health workforce; CrI, credible interval; CAF, Central African Republic; DRC, Democratic Republic of the Congo; Sao TP, Sao Tome and Principe

Supplementary Table S16: Projected coverage of age-appropriate MCV in 2030 with different scenarios

| **Country** | **Reference scenario** | **DAH reduction** | | **SHW reduction** | |
| --- | --- | --- | --- | --- | --- |
|  | **Coverage (95% CrI)** | **Coverage (95% CrI)** | **Coverage changes^1^** | **Coverage (95% CrI)** | **Coverage changes^2^** |
| **South Asia** |  |  |  |  |  |
| Bangladesh | 74.8 (66.9-81.4) | 73.7 (65.8-80.8) | 1.10 | 75.3 (66.7-82.5) | -0.50 |
| India | 53.3 (36.8-69.1) | 53.3 (36.6-68.7) | 0.00 | 53.9 (37.1-70.2) | -0.60 |
| Nepal | 81.8 (74.5-88.1) | 81.5 (72.9-88.4) | 0.30 | 80.0 (66.3-89.1) | 1.80 |
| Pakistan | 69.8 (48.9-85.9) | 68.4 (49.1-84.5) | 1.40 | 69.7 (51.3-85.7) | 0.10 |
| **Southeast Asia** |  |  |  |  |  |
| Cambodia | 86.8 (76.1-93.6) | 84.8 (71.7-93.3) | 2.00 | 86.8 (75.7-93.6) | 0.00 |
| Laos | 65.8 (52.2-76.9) | 63.6 (47.7-77.3) | 2.20 | 66.3 (53.7-77.6) | -0.50 |
| Myanmar | 40.6 (15.8-70.6) | 39.3 (14.1-70.7) | 1.30 | 41.9 (15-71.7) | -1.30 |
| Timor-Leste | 65.2 (37.9-86.5) | 64.7 (38.2-86.1) | 0.50 | 66.0 (40.6-86.2) | -0.80 |
| Vietnam | 44.7 (24.1-65.3) | 43.7 (24.9-64.8) | 1.00 | 44.7 (25.2-65.2) | 0.00 |
| **Central Africa** |  |  |  |  |  |
| Angola | 41.7 (27.1-56.7) | 41.1 (26.9-57.7) | 0.60 | 41.9 (26.4-57.4) | -0.20 |
| Cameroon | 49.9 (33.7-66.1) | 49.7 (33.5-64.4) | 0.20 | 50.7 (34.7-66.6) | -0.80 |
| CAF | 24.5 (13.1-39.2) | 24.8 (12.2-41.8) | -0.30 | 24.9 (13.3-39.4) | -0.40 |
| Chad | 20.1 (13.0-28.5) | 20.9 (13.8-30.1) | -0.80 | 20.1 (11.3-30.7) | 0.00 |
| Congo | 43.3 (17.1-73.8) | 41.7 (15.2-74.2) | 1.60 | 42.4 (19.0-70.4) | 0.90 |
| DRC | 6.1 (2.9-11.0) | 5.0 (2.3-9.7) | 1.10 | 6.8 (3.2-14.2) | -0.70 |
| STP | 84.0 (78.2-88.7) | 83.5 (77.5-88.8) | 0.50 | 84.3 (78.1-89.3) | -0.30 |
| **East Africa** |  |  |  |  |  |
| Burundi | 83.4 (69.7-92.1) | 82.2 (68.4-91.9) | 1.20 | 83.3 (69.8-92.3) | 0.10 |
| Comoros | 47.0 (17.5-78.4) | 47.6 (16.3-80.2) | -0.60 | 47.5 (19.7-77.4) | -0.50 |
| Ethiopia | 44.4 (31.1-58.6) | 43.8 (32.7-55.7) | 0.60 | 45.3 (27.0-62.7) | -0.90 |
| Kenya | 58.9 (31.4-81.2) | 57.1 (29.4-82.1) | 1.80 | 58.0 (33.8-79.7) | 0.90 |
| Madagascar | 37.5 (26.0-49.9) | 36.8 (25.0-49.7) | 0.70 | 37.5 (25.3-50.0) | 0.00 |
| Malawi | 62.3 (44.7-79.2) | 62.4 (42.1-78.2) | -0.10 | 62.2 (44.1-78.1) | 0.10 |
| Mozambique | 53.6 (19.0-85.4) | 52.9 (18.3-84.9) | 0.70 | 54.4 (15.8-87.3) | -0.80 |
| Rwanda | 72.4 (37.6-93.4) | 71.6 (38.8-93.5) | 0.80 | 68.5 (34.3-91.4) | 3.90 |
| Tanzania | 58.0 (39.0-75.8) | 57.6 (37.0-74.5) | 0.40 | 58.6 (39.3-75.7) | -0.60 |
| Uganda | 55.7 (30.5-77.8) | 57.2 (33.6-78.4) | -1.50 | 57.8 (34.8-78.3) | -2.10 |
| Zambia | 65.6 (53.3-77.4) | 64.7 (51.6-76.0) | 0.90 | 65.4 (52.3-76.8) | 0.20 |
| Zimbabwe | 84.2 (76.0-90.4) | 83.1 (73.2-90.3) | 1.10 | 84.1 (75.9-90.8) | 0.10 |
| **West Africa** |  |  |  |  |  |
| Benin | 52.4 (36.3-68.2) | 50.9 (34.1-67.0) | 1.50 | 52.5 (36.7-67.4) | -0.10 |
| Burkina Faso | 78.3 (43.1-95.6) | 78.0 (47.1-94.6) | 0.30 | 78.2 (48.9-94.6) | 0.10 |
| Cote d'Ivoire | 33.0 (15.2-54.5) | 32.7 (13.6-54.9) | 0.30 | 33.8 (15.6-54.4) | -0.80 |
| The Gambia | 65.1 (50.1-77.7) | 65.7 (52.6-77.8) | -0.60 | 65.8 (53.1-78.3) | -0.70 |
| Ghana | 68.1 (57.7-77.2) | 67.4 (56.6-76.2) | 0.70 | 68.2 (56.5-78.1) | -0.10 |
| Guinea | 20.5 (9.1-36.5) | 21.0 (8.7-37.4) | -0.50 | 21.0 (9.0-37.9) | -0.50 |
| Liberia | 54.2 (37.1-71.5) | 54.2 (37.4-70.2) | 0.00 | 54.9 (37.4-71.2) | -0.70 |
| Mali | 23.4 (14.3-34.3) | 23.5 (15.1-34.7) | -0.10 | 23.4 (15.1-33.4) | 0.00 |
| Niger | 34.1 (18.1-52.7) | 32.7 (18.2-49.5) | 1.40 | 34.9 (17.2-53.9) | -0.80 |
| Nigeria | 55.6 (40.8-69.1) | 55.7 (40.7-70.2) | -0.10 | 55.4 (41.4-68.7) | 0.20 |
| Senegal | 63.4 (53.7-71.9) | 62.6 (52.6-71.5) | 0.80 | 64.3 (51.7-75.3) | -0.90 |
| Sierra Leone | 63.3 (54.0-71.5) | 63.7 (53.8-73.0) | -0.40 | 62.9 (53.6-71.1) | 0.40 |
| Togo | 62.1 (44.9-78.1) | 61.3 (42.4-78.4) | 0.80 | 62.8 (45.0-78.8) | -0.70 |

Note: ^1^Change in the coverage was estimated as the absolute change in the coverage of age-appropriate BCG from reference scenario to the DAH reduction; ^2^Change in the coverage was estimated as the absolute change in the coverage of age-appropriate BCG from reference scenario to the SHW reduction; DAH, development assistance for health; SHW, Skilled health workforce; CrI, credible interval; CAF, Central African Republic; DRC, Democratic Republic of the Congo; Sao TP, Sao Tome and Principe

Supplementary Table S17: Posterior mean difference by considering with and without country level predictors for age appropriate BCG and DTP3 vaccine

| **country** | **Posterior mean differences by changing predictors (with and without country level predictors)** | | | | | | | | | |
| --- | --- | --- | --- | --- | --- | --- | --- | --- | --- | --- |
|  | **BCG** | | | |  | **DTP3** | | | | |
|  | **2000** | **2010** | **2020** | **2030** |  | **2000** | **2010** | **2020** | **2030** |  |
| **South Asia** |  |  |  |  |  |  |  |  |  |  |
| Bangladesh | 1.3 | 0.8 | -0.9 | -4.2 |  | 1.4 | 0.6 | -0.5 | -1.5 |  |
| India | 0.4 | 0.1 | 0.2 | -0.4 |  | 1.8 | 0.1 | -2.7 | -5.2 |  |
| Nepal | -0.8 | 4.3 | -3.1 | -5.6 |  | -2.2 | 6.8 | -5.1 | -6.4 |  |
| Pakistan | -3.5 | -1.4 | 0 | 0.1 |  | -1.5 | -2.4 | 2.7 | 9.1 |  |
| **Southeast Asia** |  |  |  |  |  |  |  |  |  |  |
| Cambodia | -2.7 | 3.2 | -1.7 | -0.9 |  | -1.5 | 2.5 | 0.0 | -1.1 |  |
| Laos | -1.7 | 4 | -2.9 | -1.9 |  | 0.2 | -0.2 | 1.1 | 1.8 |  |
| Myanmar | 1.5 | 1.1 | -0.7 | -1.2 |  | 0.2 | -0.1 | -1.7 | -2.6 |  |
| Timor-Leste | 8.2 | -0.6 | -1.6 | -4.1 |  | 9.4 | -1.4 | 2.8 | 3.0 |  |
| Vietnam | -1.9 | 0 | -1.6 | -2.6 |  | 8.1 | -0.3 | -5.1 | -7.1 |  |
| **Central Africa** |  |  |  |  |  |  |  |  |  |  |
| Angola | -1.3 | -0.7 | -0.1 | -2.5 |  | -0.2 | -1.6 | 2.5 | 2.2 |  |
| Cameroon | -0.8 | -1 | -0.7 | -6.4 |  | 0.2 | -0.4 | 0.9 | -6.1 |  |
| CAF | 1.9 | 0.2 | -0.4 | 1.7 |  | 1.2 | 0.2 | 0.0 | 9.8 |  |
| Chad | -0.6 | 2.3 | -1.5 | -5.4 |  | 0.2 | 0.2 | -1.3 | -2.5 |  |
| Congo | 13.9 | -3 | -2.4 | -3.9 |  | 15.7 | -2.2 | 5.7 | 10.9 |  |
| DRC | -3.3 | 22.9 | -29.4 | -68.8 |  | -2.2 | 13.9 | -18.2 | -51.0 |  |
| Sao TP | 0.4 | -0.1 | 0.2 | 0.2 |  | -0.4 | -0.7 | 1.4 | 1.3 |  |
| **East Africa** |  |  |  |  |  |  |  |  |  |  |
| Burundi | -8.2 | 0.1 | -0.3 | -1 |  | -11.8 | 3.4 | -0.9 | -3.4 |  |
| Comoros | -0.1 | 1 | -6.4 | -8.1 |  | -0.1 | 0.2 | 0.1 | -1.6 |  |
| Ethiopia | 0.4 | -1.4 | 0.6 | 1.9 |  | 1.4 | -2.6 | 3.1 | 11.4 |  |
| Kenya | 1.8 | -1.7 | 2 | 1.8 |  | 1.8 | -2.6 | 6.6 | 8.8 |  |
| Madagascar | -0.9 | 2.1 | -1.9 | -3.2 |  | -0.9 | 3.9 | -3.8 | -6.3 |  |
| Malawi | -0.6 | -0.4 | -2.1 | -3.1 |  | -0.7 | -0.7 | -2.7 | -6.4 |  |
| Mozambique | -4.2 | -0.7 | -2.7 | -7.1 |  | -0.4 | -1.1 | 10.0 | 14.1 |  |
| Rwanda | -1.3 | 0.2 | -0.2 | -0.4 |  | -2.5 | 1.4 | -0.8 | -1.3 |  |
| Tanzania | -3.1 | 1.1 | -3.2 | -7.4 |  | -4.3 | -1.3 | 1.1 | 2.1 |  |
| Uganda | -3.9 | 0.9 | -1.6 | -2.7 |  | -0.2 | -1.3 | -2.2 | -5.5 |  |
| Zambia | 0.8 | -7.9 | -0.9 | -1.1 |  | -0.3 | 0.2 | 0.8 | 1.2 |  |
| Zimbabwe | -14.2 | 1.9 | -1.2 | -1.3 |  | -5.3 | 0.7 | 1.8 | 1.0 |  |
| **West Africa** |  |  |  |  |  |  |  |  |  |  |
| Benin | -3.7 | 1.5 | -1.7 | -7 |  | 4.0 | 0.9 | -2.3 | -3.4 |  |
| Burkina Faso | -9.8 | 0.5 | -4.3 | -5.6 |  | -1.6 | 0.4 | 1.4 | -2.5 |  |
| Cote d'Ivoire | 1.3 | -1.4 | -0.1 | -0.1 |  | -1.0 | 4.5 | -5.3 | -9.4 |  |
| The Gambia | -0.9 | 0.8 | -0.7 | -1.6 |  | 0.7 | -1.5 | 0.8 | 4.6 |  |
| Ghana | 0.7 | 0.3 | -0.3 | -0.3 |  | 0.7 | -0.1 | 1.0 | 1.3 |  |
| Guinea | -0.4 | -4.7 | -6.9 | -13.5 |  | 2.8 | -0.6 | -3.2 | -7.2 |  |
| Liberia | -1.3 | 5.2 | -0.2 | -4.3 |  | 0.0 | 0.4 | 1.3 | -1.2 |  |
| Mali | -6.6 | 7.5 | -8.4 | -16.1 |  | -3.1 | 5.1 | -5.9 | -13.7 |  |
| Niger | 2.7 | -1.7 | -3.7 | -4.3 |  | 3.1 | -1.9 | -4.2 | -4.6 |  |
| Nigeria | -7.3 | -1.9 | 2.6 | 15.7 |  | -3.1 | -0.7 | 1.9 | 17.0 |  |
| Senegal | -1.5 | -0.2 | -0.3 | -1.2 |  | -1.1 | -0.3 | -0.7 | -3.0 |  |
| Sierra Leone | 5.3 | -2.6 | 0.6 | 0.4 |  | 6.8 | -4.5 | 2.0 | 6.5 |  |
| Togo | 0.2 | 0.8 | -1.9 | -5.1 |  | 1.6 | -11.4 | 14.6 | 22.1 |  |
| Median | -0.8 | 0.2 | -1.2 | -2.6 |  | -0.1 | -0.2 | 0.1 | -1.3 |  |

Note: CAF, Central African Republic; DRC, Democratic Republic of the Congo; Sao TP, Sao Tome and Principe

Supplementary Table S18: Posterior mean difference by considering with and without country level predictors for age appropriate polio3 and MCV vaccines

| **country** | **Posterior mean differences by changing predictors (with and without country level predictors)** | | | | | | | | |
| --- | --- | --- | --- | --- | --- | --- | --- | --- | --- |
|  | **polio3** | | | |  | **MCV** | | | |
|  | **2000** | **2010** | **2020** | **2030** |  | **2000** | **2010** | **2020** | **2030** |
| **South Asia** |  |  |  |  |  |  |  |  |  |
| Bangladesh | 0.5 | 0.1 | -0.4 | -1.3 |  | 3.9 | 1.7 | -0.5 | -1 |
| India | 1.6 | 0.0 | -2.7 | -4.0 |  | 0.7 | 0 | -1.2 | -2.2 |
| Nepal | -1.2 | 1.4 | -2.0 | -1.8 |  | -1.7 | 4.7 | -2.2 | -2.7 |
| Pakistan | -0.8 | -2.2 | 1.1 | 3.4 |  | -3.8 | -2.8 | 2.9 | 8 |
| **Southeast Asia** |  |  |  |  |  |  |  |  |  |
| Cambodia | -1.1 | 1.9 | 0.1 | -1.3 |  | -3.5 | 4.6 | -7.6 | -6 |
| Laos | 0.8 | -0.9 | 1.9 | 1.8 |  | -0.4 | 2.8 | -2.7 | -4.1 |
| Myanmar | 0.1 | 1.1 | -1.4 | -2.3 |  | 0.8 | 3.7 | -1.8 | -1 |
| Timor-Leste | 2.8 | -0.9 | -0.2 | -0.7 |  | 1.2 | -0.7 | 0.8 | 1.6 |
| Vietnam | 3.0 | -0.5 | -3.5 | -4.5 |  | 5.2 | -0.1 | -4.6 | -7.3 |
| **Central Africa** |  |  |  |  |  |  |  |  |  |
| Angola | -0.1 | -2.2 | 1.8 | 0.6 |  | -0.7 | -1.9 | 2.2 | 1.2 |
| Cameroon | -2.0 | -2.3 | -0.3 | -12.7 |  | 0.9 | -0.3 | 0.8 | -8.2 |
| CAF | 0.3 | 0.3 | 0.4 | 4.9 |  | 0.7 | 0.1 | -0.3 | 2.8 |
| Chad | -0.1 | 0.4 | -1.8 | -2.6 |  | -0.1 | 0.7 | 0 | -2.4 |
| Congo | -1.2 | 2.0 | 4.6 | 7.8 |  | -11.9 | 5.5 | 2.9 | 3.9 |
| DRC | -2.3 | 14.1 | -20.9 | -57.1 |  | -2.4 | 14.4 | -16.8 | -47.3 |
| Sao TP | -0.1 | -0.1 | 1.1 | 1.0 |  | 0.2 | -2.1 | 0.2 | 0.4 |
| **East Africa** |  |  |  |  |  |  |  |  |  |
| Burundi | -6.4 | 0.6 | -0.6 | -2.9 |  | -9.4 | 0.8 | -1 | -5 |
| Comoros | -0.5 | 0.5 | -3.6 | -5.9 |  | 0.5 | 0.1 | -2.7 | -5.1 |
| Ethiopia | 2.3 | -2.9 | 3.1 | 11.2 |  | 1.1 | -2.3 | 2.3 | 9.1 |
| Kenya | 0.2 | -0.8 | 1.0 | -0.4 |  | 0.3 | -0.2 | -1 | -2.5 |
| Madagascar | -1.9 | 4.9 | -2.9 | -5.1 |  | -1.4 | 5.2 | -5.5 | -9.5 |
| Malawi | -0.7 | -0.6 | -1.2 | -3.1 |  | -0.5 | -0.5 | -1.4 | -4 |
| Mozambique | -0.9 | -0.9 | 10.2 | 14.9 |  | -1.3 | -0.6 | -1 | -5.4 |
| Rwanda | -2.2 | 1.1 | -1.3 | -2.0 |  | -1.7 | 1.4 | -9.2 | -13.4 |
| Tanzania | -4.4 | 0.0 | 0.1 | -0.6 |  | -2.4 | 0.6 | -2.1 | -4.9 |
| Uganda | 0.1 | -1.5 | -2.9 | -6.6 |  | 0 | -1.7 | -3.7 | -9.3 |
| Zambia | -0.1 | -0.8 | 0.5 | 0.6 |  | 1.4 | -4 | -0.1 | 0.2 |
| Zimbabwe | -4.4 | 0.8 | 0.9 | 0.3 |  | -11 | 0.9 | 0.1 | -0.6 |
| **West Africa** |  |  |  |  |  |  |  |  |  |
| Benin | 3.8 | -0.1 | -1.1 | -1.6 |  | -3.3 | 1.8 | -1.8 | -6.3 |
| Burkina Faso | -1.6 | 0.1 | 0.6 | -2.3 |  | -9.5 | 2.8 | -5.5 | -9.2 |
| Cote d'Ivoire | -1.9 | 5.5 | -5.7 | -11.4 |  | -0.8 | 3.2 | -4.5 | -8.2 |
| The Gambia | 1.4 | -2.4 | 0.7 | 1.5 |  | 1.1 | -1.4 | -0.2 | -1.7 |
| Ghana | -1.4 | 0.4 | -0.7 | -0.7 |  | -4.2 | 1 | -1.1 | -1.3 |
| Guinea | 1.1 | 0.3 | -1.2 | -2.3 |  | 1 | -0.3 | -2.7 | -4.7 |
| Liberia | 0.2 | 1.0 | 0.1 | -3.2 |  | -0.5 | 1.4 | 0.2 | -3.7 |
| Mali | -2.5 | 5.5 | -5.9 | -12.1 |  | -6.3 | 12 | -11.7 | -23.5 |
| Niger | 2.0 | -0.5 | -3.1 | -4.1 |  | 2.9 | -1.1 | -5.3 | -6.6 |
| Nigeria | -5.0 | -0.7 | 0.8 | 14.4 |  | -2.6 | -0.3 | 0.6 | 9.9 |
| Senegal | -0.3 | -0.1 | -0.8 | -2.6 |  | -3.1 | -0.9 | -0.5 | -2.6 |
| Sierra Leone | 9.2 | -4.9 | 2.2 | 4.5 |  | 3.6 | -2.8 | 0.9 | 4 |
| Togo | 0.4 | -3.7 | 2.7 | 8.5 |  | -0.4 | 1.2 | -1.4 | -5.5 |
| Median | -0.1 | 0.0 | -0.3 | -1.6 |  | -0.5 | 0.1 | -1.1 | -3.7 |

Note: CAF, Central African Republic; DRC, Democratic Republic of the Congo; Sao TP, Sao Tome and Principe

Supplementary Table S19: Posterior mean difference by altering prior distribution on hyperparameters for age-appropriate BCG and DTP3 vaccine

| **country** | **Posterior mean differences by changing priors (vague vs weekly)** | | | | | | | | |
| --- | --- | --- | --- | --- | --- | --- | --- | --- | --- |
|  | **BCG** | | | |  | **DTP3** | | | |
|  | **2000** | **2010** | **2020** | **2030** |  | **2000** | **2010** | **2020** | **2030** |
| **South Asia** |  |  |  |  |  |  |  |  |  |
| Bangladesh | -0.1 | -0.1 | -0.2 | -0.3 |  | 0 | -0.1 | -0.1 | -0.1 |
| India | 0.1 | 0.1 | 0 | 0.1 |  | 0.3 | 0.1 | -0.2 | -0.6 |
| Nepal | -0.1 | 0.3 | 0 | 0.2 |  | 0 | -0.3 | 0 | -0.4 |
| Pakistan | -0.5 | 0.1 | -0.1 | 0 |  | -0.5 | -0.1 | -0.2 | -0.1 |
| **Southeast Asia** |  |  |  |  |  |  |  |  |  |
| Cambodia | 0 | -0.1 | -0.3 | -0.1 |  | 0 | 0.1 | 0.1 | 0 |
| Laos | 0 | -0.1 | 0 | 0.1 |  | 0 | 0 | -0.3 | -0.4 |
| Myanmar | 0.3 | -0.1 | 0.3 | 0.5 |  | 0 | 0.3 | -0.4 | -1.3 |
| Timor-Leste | 1.8 | 0.2 | -0.1 | -0.6 |  | 0.9 | 0.2 | -0.4 | -1 |
| Vietnam | 0.4 | -0.1 | 0 | -0.1 |  | 0.6 | 0.1 | 0.2 | 0.9 |
| **Central Africa** |  |  |  |  |  |  |  |  |  |
| Angola | -0.2 | 0.4 | -0.1 | -0.2 |  | 0 | 0 | -0.1 | 0 |
| Cameroon | 0.3 | 0.2 | 0.1 | 0.4 |  | -0.2 | -0.2 | -0.1 | -0.6 |
| CAF | 0.4 | 0.1 | -0.2 | -1.2 |  | 0 | 0 | 0 | -1.4 |
| Chad | 0.1 | 0 | -0.1 | -0.1 |  | -0.1 | 0 | 0 | 0.1 |
| Congo | -0.1 | -0.1 | 0.1 | 0.3 |  | -0.4 | 0.1 | -0.5 | -0.7 |
| DRC | 0 | 0.1 | 0.2 | 0.2 |  | 0 | 0 | 0 | 0.1 |
| Sao TP | -0.1 | -0.1 | 0 | 0 |  | 0 | 0.2 | 0.2 | 0.1 |
| **East Africa** |  |  |  |  |  |  |  |  |  |
| Burundi | -0.3 | 0.2 | 0 | 0.1 |  | 0.2 | 0.1 | 0.3 | 0.8 |
| Comoros | -0.2 | -0.1 | -0.6 | -0.6 |  | 0 | -0.1 | 0.9 | 1.4 |
| Ethiopia | -0.1 | 0.1 | 0.1 | -0.3 |  | -0.1 | 0.2 | -0.1 | -0.6 |
| Kenya | 0 | -0.2 | -0.5 | -0.6 |  | -0.1 | -0.1 | 0.4 | 1 |
| Madagascar | -0.1 | 0.1 | 0.1 | 0.1 |  | 0.1 | 0.1 | 0.1 | 0.2 |
| Malawi | 0.2 | 0.1 | 0.2 | 0.3 |  | 0.1 | 0.1 | -0.2 | -0.3 |
| Mozambique | -0.4 | 0.1 | 0.1 | 0.2 |  | -0.1 | -0.1 | 0.8 | 1.6 |
| Rwanda | -0.2 | 0 | 0 | -0.1 |  | 0.1 | 0 | 0.1 | 0.2 |
| Tanzania | 0.7 | -0.2 | -0.2 | -0.1 |  | -1.5 | 0.1 | -0.2 | -0.4 |
| Uganda | -0.3 | -0.2 | -0.3 | -0.4 |  | 0 | 0 | 0.2 | 0.5 |
| Zambia | -0.5 | -1 | -0.4 | -0.6 |  | 0 | -0.6 | -0.4 | -0.6 |
| Zimbabwe | 0.2 | 0.1 | -0.1 | -0.2 |  | 0 | -0.1 | 0.1 | 0.1 |
| **West Africa** |  |  |  |  |  |  |  |  |  |
| Benin | 0.2 | 0 | -0.2 | -0.3 |  | 0.3 | 0.1 | -0.1 | 0.2 |
| Burkina Faso | -0.4 | -0.2 | -0.5 | -0.6 |  | 0.1 | 0.1 | 0.8 | 1.4 |
| Cote d'Ivoire | -0.1 | 0.1 | 0.1 | -0.2 |  | -0.2 | 0.5 | -0.8 | -2.4 |
| The Gambia | 0.1 | 0 | 0.1 | -0.4 |  | 0 | 0 | 0.1 | 0.3 |
| Ghana | -0.6 | -0.1 | -0.1 | 0 |  | 0.3 | 0.1 | 0.2 | 0.1 |
| Guinea | 0.9 | 0 | -0.1 | -0.6 |  | 0.4 | -0.3 | -0.4 | -0.9 |
| Liberia | -0.1 | -0.1 | 0.2 | 0.5 |  | 0 | 0.3 | 0 | -0.8 |
| Mali | 0.1 | 0.1 | 0.1 | 0.2 |  | 0.3 | 0 | 0 | 0.4 |
| Niger | -0.2 | -0.1 | 0.4 | 0.6 |  | 0.1 | 0 | 0.1 | 0.3 |
| Nigeria | 0.1 | 0.1 | 0 | -0.1 |  | 0.1 | 0 | 0 | -0.1 |
| Senegal | 0.5 | 0.2 | -0.1 | -0.3 |  | -0.2 | 0 | 0.2 | 0.4 |
| Sierra Leone | -0.4 | 0 | 0 | 0 |  | -0.1 | 0 | 0 | 0.1 |
| Togo | 0 | -0.1 | -0.4 | -0.5 |  | 0.1 | 0.4 | -0.4 | -0.2 |
| Median | 0 | 0 | 0 | -0.1 |  | 0 | 0 | 0 | 0 |

Note: CAF, Central African Republic; DRC, Democratic Republic of the Congo; Sao TP, Sao Tome and Principe

Supplementary Table S20: Posterior mean difference by altering prior distribution on hyperparameters for age-appropriate polio3 and MCV vaccines

| **country** | **Posterior mean differences by changing priors (vague vs weekly)** | | | | | | | | |
| --- | --- | --- | --- | --- | --- | --- | --- | --- | --- |
|  | **Polio3** | | | |  | **MCV** | | | |
|  | **2000** | **2010** | **2020** | **2030** |  | **2000** | **2010** | **2020** | **2030** |
| **South Asia** |  |  |  |  |  |  |  |  |  |
| Bangladesh | -0.1 | -0.1 | 0 | 0.1 |  | -0.2 | -0.1 | 0.1 | 0.3 |
| India | -0.1 | 0 | 0 | 0.1 |  | -0.2 | -0.2 | -0.2 | 0 |
| Nepal | -0.3 | -0.1 | 0.1 | 0.5 |  | 0 | -0.1 | -0.2 | -0.4 |
| Pakistan | -0.2 | -0.1 | 0.1 | 0.4 |  | -0.2 | -0.2 | 0.4 | 0.7 |
| **Southeast Asia** |  |  |  |  |  |  |  |  |  |
| Cambodia | 0 | 0.2 | 0.1 | 0.1 |  | 0 | -0.2 | 0 | 0.1 |
| Laos | 0.1 | 0.1 | -0.1 | -0.3 |  | 0.1 | -0.1 | -0.1 | -0.2 |
| Myanmar | -0.1 | 0.8 | -0.3 | 0.3 |  | -0.2 | 0.1 | -0.5 | -0.6 |
| Timor-Leste | -0.1 | -0.1 | 0.2 | 0.6 |  | 0.7 | -0.3 | -0.1 | -0.2 |
| Vietnam | 0 | 0.1 | 0 | -0.1 |  | 0.4 | -0.1 | 0 | 0.2 |
| **Central Africa** |  |  |  |  |  |  |  |  |  |
| Angola | 0 | 0 | 0 | 0.3 |  | 0 | 0 | 0 | 0.2 |
| Cameroon | 0.1 | 0 | -0.1 | 0.2 |  | -0.1 | -0.2 | -0.3 | -0.8 |
| CAF | 0.1 | 0 | 0 | -1 |  | 0.1 | 0.1 | 0.2 | 0.5 |
| Chad | 0 | 0 | 0.2 | 0 |  | 0 | -0.1 | -0.1 | -0.4 |
| Congo | 0.3 | 0.1 | 0.5 | 0.8 |  | 0.3 | -0.1 | 1 | 2 |
| DRC | 0 | 0.2 | 0 | -0.2 |  | 0 | 0 | 0 | 0 |
| Sao TP | 0.3 | 0.1 | -0.1 | -0.1 |  | 0.1 | 0.2 | -0.1 | -0.2 |
| **East Africa** |  |  |  |  |  |  |  |  |  |
| Burundi | -0.1 | 0.1 | 0.1 | 0.1 |  | -0.7 | 0.1 | -0.1 | -0.4 |
| Comoros | 0 | -0.2 | 0.3 | 0.6 |  | -0.1 | -0.2 | -0.4 | -0.7 |
| Ethiopia | 0 | 0 | 0.2 | 0.6 |  | -0.1 | 0 | -0.1 | -0.2 |
| Kenya | -0.1 | -0.1 | 0.4 | 0.8 |  | -0.1 | 0 | 0.4 | 0.6 |
| Madagascar | -0.1 | 0.1 | 0 | 0.2 |  | -0.2 | 0.2 | -0.1 | -0.1 |
| Malawi | -0.2 | -0.1 | 0 | 0 |  | 0.2 | 0 | -0.1 | -0.4 |
| Mozambique | 0.1 | 0 | -0.3 | -0.4 |  | -0.2 | -0.1 | 1 | 1.4 |
| Rwanda | 0 | -0.1 | -0.1 | -0.1 |  | 0.1 | 0 | 1 | 1.4 |
| Tanzania | 0.2 | 0.3 | -0.1 | -0.3 |  | 0.4 | -0.1 | 0 | 0.2 |
| Uganda | 0 | 0 | -0.1 | 0 |  | 0.2 | 0 | 0 | 0 |
| Zambia | 0 | -0.1 | -0.1 | -0.1 |  | 0.4 | 0.1 | 0 | 0.2 |
| Zimbabwe | -0.1 | 0.1 | 0.4 | 0.3 |  | -0.2 | 0 | 0 | -0.1 |
| **West Africa** |  |  |  |  |  |  |  |  |  |
| Benin | 0.1 | -0.1 | -0.1 | 0.1 |  | 0.1 | 0.3 | 0.1 | -0.2 |
| Burkina Faso | 0 | 0 | 0.2 | 0.1 |  | 0.3 | 0 | 0.4 | 0.2 |
| Cote d'Ivoire | -0.1 | -0.1 | 0.1 | 0.3 |  | 0 | -0.1 | 0.1 | 0.2 |
| The Gambia | -0.1 | -0.1 | -0.2 | 0.4 |  | -0.2 | 0 | 0 | -0.4 |
| Ghana | -0.3 | -0.1 | -0.2 | -0.1 |  | -0.1 | 0 | 0.2 | 0.5 |
| Guinea | 0.2 | -0.1 | -0.2 | -0.5 |  | 0.6 | 0.1 | 0 | -0.4 |
| Liberia | 0.2 | 0.2 | -0.2 | -0.6 |  | 0 | -0.2 | -0.2 | -0.3 |
| Mali | -0.1 | -0.1 | -0.1 | -0.4 |  | 0 | 0.2 | -0.1 | 0 |
| Niger | 0 | 0 | 0.5 | 1 |  | 0.1 | -0.1 | -0.3 | -0.4 |
| Nigeria | 0.1 | 0 | 0.1 | -0.1 |  | -0.1 | 0 | -0.1 | 0.5 |
| Senegal | -0.2 | -0.1 | 0 | 0.3 |  | 0.4 | 0.2 | -0.1 | -0.3 |
| Sierra Leone | 0.1 | -0.1 | 0.1 | 0.4 |  | 0 | -0.1 | 0.1 | 0.4 |
| Togo | 0 | -0.3 | -0.1 | 0.3 |  | 0 | 0.1 | 0.1 | 0.2 |
| Median | 0 | 0 | 0 | 0.1 |  | 0 | 0 | 0 | 0 |

Note: CAF, Central African Republic; DRC, Democratic Republic of the Congo; Sao TP, Sao Tome and Principe

Supplementary Table S21: National level estimate of Gelman Rubin Potential scale reduction factors (PSRF) for age-appropriate BCG and DTP3

| **country** | **Potential scale reduction factors (Point estimate; Upper CrI)** | | | | | | | | | |
| --- | --- | --- | --- | --- | --- | --- | --- | --- | --- | --- |
|  | **BCG** | | | |  | | **DTP3** | | | |
|  | **2000** | **2010** | **2020** | **2030** |  | **2000** | | **2010** | **2020** | **2030** |
| **South Asia** |  |  |  |  |  |  | |  |  |  |
| Bangladesh | (1.002;1.012) | (1.002;1.012) | (1.002;1.012) | (1.002;1.012) |  | (1.007;1.027) | | (1.007;1.027) | (1.007;1.027) | (1.007;1.027) |
| India | (1.002;1.012) | (1.002;1.012) | (1.002;1.012) | (1.002;1.012) |  | (1.007;1.027) | | (1.007;1.027) | (1.007;1.027) | (1.007;1.027) |
| Nepal | (1.002;1.012) | (1.002;1.012) | (1.002;1.012) | (1.002;1.012) |  | (1.007;1.027) | | (1.007;1.027) | (1.007;1.027) | (1.007;1.027) |
| Pakistan | (1.002;1.012) | (1.002;1.012) | (1.002;1.012) | (1.002;1.012) |  | (1.007;1.027) | | (1.007;1.027) | (1.007;1.027) | (1.007;1.027) |
| **Southeast Asia** |  |  |  |  |  |  | |  |  |  |
| Cambodia | (1.002;1.012) | (1.002;1.012) | (1.002;1.012) | (1.002;1.012) |  | (1.007;1.027) | | (1.007;1.027) | (1.007;1.027) | (1.007;1.027) |
| Laos | (1.002;1.012) | (1.002;1.012) | (1.002;1.012) | (1.002;1.012) |  | (1.007;1.027) | | (1.007;1.027) | (1.007;1.027) | (1.007;1.027) |
| Myanmar | (1.002;1.012) | (1.002;1.012) | (1.002;1.012) | (1.002;1.012) |  | (1.007;1.027) | | (1.007;1.027) | (1.007;1.027) | (1.007;1.027) |
| Timor-Leste | (1.002;1.012) | (1.002;1.012) | (1.002;1.012) | (1.002;1.012) |  | (1.007;1.027) | | (1.007;1.027) | (1.007;1.027) | (1.007;1.027) |
| Vietnam | (1.002;1.012) | (1.002;1.012) | (1.002;1.012) | (1.002;1.012) |  | (1.007;1.027) | | (1.007;1.027) | (1.007;1.027) | (1.007;1.027) |
| **Central Africa** |  |  |  |  |  |  | |  |  |  |
| Angola | (1.002;1.015) | (1.002;1.012) | (1.002;1.012) | (1.002;1.012) |  | (1.002;1.011) | | (1.007;1.027) | (1.007;1.027) | (1.007;1.027) |
| Cameroon | (1.002;1.012) | (1.002;1.012) | (1.002;1.012) | (1.002;1.012) |  | (1.007;1.027) | | (1.007;1.027) | (1.007;1.027) | (1.007;1.027) |
| CAF | (1.002;1.012) | (1.002;1.012) | (1.002;1.012) | (1.002;1.012) |  | (1.007;1.027) | | (1.007;1.027) | (1.007;1.027) | (1.007;1.027) |
| Chad | (1.002;1.012) | (1.002;1.012) | (1.002;1.012) | (1.002;1.012) |  | (1.007;1.027) | | (1.007;1.027) | (1.007;1.027) | (1.007;1.027) |
| Congo | (1.002;1.012) | (1.002;1.012) | (1.002;1.012) | (1.002;1.012) |  | (1.007;1.027) | | (1.007;1.027) | (1.007;1.027) | (1.007;1.027) |
| DRC | (1.002;1.012) | (1.002;1.012) | (1.002;1.012) | (1.002;1.012) |  | (1.007;1.027) | | (1.007;1.027) | (1.007;1.027) | (1.007;1.027) |
| Sao TP | (1.002;1.012) | (1.002;1.012) | (1.002;1.012) | (1.002;1.012) |  | (1.007;1.027) | | (1.007;1.027) | (1.007;1.027) | (1.007;1.027) |
| **East Africa** |  |  |  |  |  |  | |  |  |  |
| Burundi | (1.002;1.012) | (1.002;1.012) | (1.002;1.012) | (1.002;1.012) |  | (1.007;1.027) | | (1.007;1.027) | (1.007;1.027) | (1.007;1.027) |
| Comoros | (1.002;1.012) | (1.002;1.012) | (1.002;1.012) | (1.002;1.012) |  | (1.007;1.027) | | (1.007;1.027) | (1.007;1.027) | (1.007;1.027) |
| Ethiopia | (1.002;1.012) | (1.002;1.012) | (1.002;1.012) | (1.002;1.012) |  | (1.007;1.027) | | (1.007;1.027) | (1.007;1.027) | (1.007;1.027) |
| Kenya | (1.002;1.012) | (1.002;1.012) | (1.002;1.012) | (1.002;1.012) |  | (1.007;1.027) | | (1.007;1.027) | (1.007;1.027) | (1.007;1.027) |
| Madagascar | (1.002;1.012) | (1.002;1.012) | (1.002;1.012) | (1.002;1.012) |  | (1.007;1.027) | | (1.007;1.027) | (1.007;1.027) | (1.007;1.027) |
| Malawi | (1.002;1.012) | (1.002;1.012) | (1.002;1.012) | (1.002;1.012) |  | (1.007;1.027) | | (1.007;1.027) | (1.007;1.027) | (1.007;1.027) |
| Mozambique | (1.002;1.012) | (1.002;1.012) | (1.002;1.012) | (1.002;1.012) |  | (1.007;1.027) | | (1.007;1.027) | (1.007;1.027) | (1.007;1.027) |
| Rwanda | (1.002;1.012) | (1.002;1.012) | (1.002;1.012) | (1.002;1.012) |  | (1.007;1.027) | | (1.007;1.027) | (1.007;1.027) | (1.007;1.027) |
| Tanzania | (1.002;1.012) | (1.002;1.012) | (1.002;1.012) | (1.002;1.012) |  | (1.007;1.027) | | (1.007;1.027) | (1.007;1.027) | (1.007;1.027) |
| Uganda | (1.002;1.012) | (1.002;1.012) | (1.002;1.012) | (1.002;1.012) |  | (1.007;1.027) | | (1.007;1.027) | (1.007;1.027) | (1.007;1.027) |
| Zambia | (1.002;1.012) | (1.002;1.012) | (1.002;1.012) | (1.002;1.012) |  | (1.007;1.027) | | (1.007;1.027) | (1.007;1.027) | (1.007;1.027) |
| Zimbabwe | (1.002;1.012) | (1.002;1.012) | (1.002;1.012) | (1.002;1.012) |  | (1.007;1.027) | | (1.007;1.027) | (1.007;1.027) | (1.007;1.027) |
| **West Africa** |  |  |  |  |  |  | |  |  |  |
| Benin | (1.002;1.012) | (1.002;1.012) | (1.002;1.012) | (1.002;1.012) |  | (1.007;1.027) | | (1.007;1.027) | (1.007;1.027) | (1.007;1.027) |
| Burkina Faso | (1.002;1.012) | (1.002;1.012) | (1.002;1.012) | (1.002;1.012) |  | (1.007;1.027) | | (1.007;1.027) | (1.007;1.027) | (1.007;1.027) |
| Cote d'Ivoire | (1.002;1.012) | (1.002;1.012) | (1.002;1.012) | (1.002;1.012) |  | (1.007;1.027) | | (1.007;1.027) | (1.007;1.027) | (1.007;1.027) |
| The Gambia | (1.002;1.012) | (1.002;1.012) | (1.002;1.012) | (1.002;1.012) |  | (1.007;1.027) | | (1.007;1.027) | (1.007;1.027) | (1.007;1.027) |
| Ghana | (1.002;1.012) | (1.002;1.012) | (1.002;1.012) | (1.002;1.012) |  | (1.007;1.027) | | (1.007;1.027) | (1.007;1.027) | (1.007;1.027) |
| Guinea | (1.002;1.012) | (1.002;1.012) | (1.002;1.012) | (1.002;1.012) |  | (1.007;1.027) | | (1.007;1.027) | (1.007;1.027) | (1.007;1.027) |
| Liberia | (1.002;1.012) | (1.002;1.012) | (1.002;1.012) | (1.002;1.012) |  | (1.007;1.027) | | (1.007;1.027) | (1.007;1.027) | (1.007;1.027) |
| Mali | (1.002;1.012) | (1.002;1.012) | (1.002;1.012) | (1.002;1.012) |  | (1.007;1.027) | | (1.007;1.027) | (1.007;1.027) | (1.007;1.027) |
| Niger | (1.002;1.012) | (1.002;1.012) | (1.002;1.012) | (1.002;1.012) |  | (1.007;1.027) | | (1.007;1.027) | (1.007;1.027) | (1.007;1.027) |
| Nigeria | (1.002;1.012) | (1.002;1.012) | (1.002;1.012) | (1.002;1.012) |  | (1.007;1.027) | | (1.007;1.027) | (1.007;1.027) | (1.007;1.027) |
| Senegal | (1.002;1.012) | (1.002;1.012) | (1.002;1.012) | (1.002;1.012) |  | (1.007;1.027) | | (1.007;1.027) | (1.007;1.027) | (1.007;1.027) |
| Sierra Leone | (1.002;1.012) | (1.002;1.012) | (1.002;1.012) | (1.002;1.012) |  | (1.007;1.027) | | (1.007;1.027) | (1.007;1.027) | (1.007;1.027) |
| Togo | (1.002;1.012) | (1.002;1.012) | (1.002;1.012) | (1.002;1.012) |  | (1.007;1.027) | | (1.007;1.027) | (1.007;1.027) | (1.007;1.027) |

Note: CAF, Central African Republic; DRC, Democratic Republic of the Congo; Sao TP, Sao Tome and Principe

Supplementary Table S22: National level estimate of Gelman Rubin Potential scale reduction factors (PSRF) for age-appropriate polio3 and MCV

| **country** | **Potential scale reduction factors (Point estimate; Upper CrI)** | | | | | | | | | | |
| --- | --- | --- | --- | --- | --- | --- | --- | --- | --- | --- | --- |
|  | **polio3** | | | | |  | | **MCV** | | | |
|  | **2000** | **2010** | **2020** | **2030** |  | | **2000** | | **2010** | **2020** | **2030** |
| **South Asia** |  |  |  |  |  | |  | |  |  |  |
| Bangladesh | (1.004;1.02) | (1.004;1.02) | (1.004;1.02) | (1.004;1.02) |  | | (1.001;1.002) | | (1.001;1.002) | (1.001;1.002) | (1.001;1.002) |
| India | (1.004;1.02) | (1.004;1.02) | (1.004;1.02) | (1.004;1.02) |  | | (1.001;1.002) | | (1.001;1.002) | (1.001;1.002) | (1.001;1.002) |
| Nepal | (1.004;1.02) | (1.004;1.02) | (1.004;1.02) | (1.004;1.02) |  | | (1.001;1.002) | | (1.001;1.002) | (1.001;1.002) | (1.001;1.002) |
| Pakistan | (1.004;1.02) | (1.004;1.02) | (1.004;1.02) | (1.004;1.02) |  | | (1.001;1.002) | | (1.001;1.002) | (1.001;1.002) | (1.001;1.002) |
| **Southeast Asia** |  |  |  |  |  | |  | |  |  |  |
| Cambodia | (1.004;1.02) | (1.004;1.02) | (1.004;1.02) | (1.004;1.02) |  | | (1.001;1.002) | | (1.001;1.002) | (1.001;1.002) | (1.001;1.002) |
| Laos | (1.004;1.02) | (1.004;1.02) | (1.004;1.02) | (1.004;1.02) |  | | (1.001;1.002) | | (1.001;1.002) | (1.001;1.002) | (1.001;1.002) |
| Myanmar | (1.004;1.02) | (1.004;1.02) | (1.004;1.02) | (1.004;1.02) |  | | (1.001;1.002) | | (1.001;1.002) | (1.001;1.002) | (1.001;1.002) |
| Timor-Leste | (1.004;1.02) | (1.004;1.02) | (1.004;1.02) | (1.004;1.02) |  | | (1.001;1.002) | | (1.001;1.002) | (1.001;1.002) | (1.001;1.002) |
| Vietnam | (1.004;1.02) | (1.004;1.02) | (1.004;1.02) | (1.004;1.02) |  | | (1.001;1.002) | | (1.001;1.002) | (1.001;1.002) | (1.001;1.002) |
| **Central Africa** |  |  |  |  |  | |  | |  |  |  |
| Angola | (1.004;1.022) | (1.004;1.02) | (1.004;1.02) | (1.004;1.02) |  | | (1;1.002) | | (1.001;1.002) | (1.001;1.002) | (1.001;1.002) |
| Cameroon | (1.004;1.02) | (1.004;1.02) | (1.004;1.02) | (1.004;1.02) |  | | (1.001;1.002) | | (1.001;1.002) | (1.001;1.002) | (1.001;1.002) |
| CAF | (1.004;1.02) | (1.004;1.02) | (1.004;1.02) | (1.004;1.02) |  | | (1.001;1.002) | | (1.001;1.002) | (1.001;1.002) | (1.001;1.002) |
| Chad | (1.004;1.02) | (1.004;1.02) | (1.004;1.02) | (1.004;1.02) |  | | (1.001;1.002) | | (1.001;1.002) | (1.001;1.002) | (1.001;1.002) |
| Congo | (1.004;1.02) | (1.004;1.02) | (1.004;1.02) | (1.004;1.02) |  | | (1.001;1.002) | | (1.001;1.002) | (1.001;1.002) | (1.001;1.002) |
| DRC | (1.004;1.02) | (1.004;1.02) | (1.004;1.02) | (1.004;1.02) |  | | (1.001;1.002) | | (1.001;1.002) | (1.001;1.002) | (1.001;1.002) |
| Sao TP | (1.004;1.02) | (1.004;1.02) | (1.004;1.02) | (1.004;1.02) |  | | (1.001;1.002) | | (1.001;1.002) | (1.001;1.002) | (1.001;1.002) |
| **East Africa** |  |  |  |  |  | |  | |  |  |  |
| Burundi | (1.004;1.02) | (1.004;1.02) | (1.004;1.02) | (1.004;1.02) |  | | (1.001;1.002) | | (1.001;1.002) | (1.001;1.002) | (1.001;1.002) |
| Comoros | (1.004;1.02) | (1.004;1.02) | (1.004;1.02) | (1.004;1.02) |  | | (1.001;1.002) | | (1.001;1.002) | (1.001;1.002) | (1.001;1.002) |
| Ethiopia | (1.004;1.02) | (1.004;1.02) | (1.004;1.02) | (1.004;1.02) |  | | (1.001;1.002) | | (1.001;1.002) | (1.001;1.002) | (1.001;1.002) |
| Kenya | (1.004;1.02) | (1.004;1.02) | (1.004;1.02) | (1.004;1.02) |  | | (1.001;1.002) | | (1.001;1.002) | (1.001;1.002) | (1.001;1.002) |
| Madagascar | (1.004;1.02) | (1.004;1.02) | (1.004;1.02) | (1.004;1.02) |  | | (1.001;1.002) | | (1.001;1.002) | (1.001;1.002) | (1.001;1.002) |
| Malawi | (1.004;1.02) | (1.004;1.02) | (1.004;1.02) | (1.004;1.02) |  | | (1.001;1.002) | | (1.001;1.002) | (1.001;1.002) | (1.001;1.002) |
| Mozambique | (1.004;1.02) | (1.004;1.02) | (1.004;1.02) | (1.004;1.02) |  | | (1.001;1.002) | | (1.001;1.002) | (1.001;1.002) | (1.001;1.002) |
| Rwanda | (1.004;1.02) | (1.004;1.02) | (1.004;1.02) | (1.004;1.02) |  | | (1.001;1.002) | | (1.001;1.002) | (1.001;1.002) | (1.001;1.002) |
| Tanzania | (1.004;1.02) | (1.004;1.02) | (1.004;1.02) | (1.004;1.02) |  | | (1.001;1.002) | | (1.001;1.002) | (1.001;1.002) | (1.001;1.002) |
| Uganda | (1.004;1.02) | (1.004;1.02) | (1.004;1.02) | (1.004;1.02) |  | | (1.001;1.002) | | (1.001;1.002) | (1.001;1.002) | (1.001;1.002) |
| Zambia | (1.004;1.02) | (1.004;1.02) | (1.004;1.02) | (1.004;1.02) |  | | (1.001;1.002) | | (1.001;1.002) | (1.001;1.002) | (1.001;1.002) |
| Zimbabwe | (1.004;1.02) | (1.004;1.02) | (1.004;1.02) | (1.004;1.02) |  | | (1.001;1.002) | | (1.001;1.002) | (1.001;1.002) | (1.001;1.002) |
| **West Africa** |  |  |  |  |  | |  | |  |  |  |
| Benin | (1.004;1.02) | (1.004;1.02) | (1.004;1.02) | (1.004;1.02) |  | | (1.001;1.002) | | (1.001;1.002) | (1.001;1.002) | (1.001;1.002) |
| Burkina Faso | (1.004;1.02) | (1.004;1.02) | (1.004;1.02) | (1.004;1.02) |  | | (1.001;1.002) | | (1.001;1.002) | (1.001;1.002) | (1.001;1.002) |
| Cote d'Ivoire | (1.004;1.02) | (1.004;1.02) | (1.004;1.02) | (1.004;1.02) |  | | (1.001;1.002) | | (1.001;1.002) | (1.001;1.002) | (1.001;1.002) |
| The Gambia | (1.004;1.02) | (1.004;1.02) | (1.004;1.02) | (1.004;1.02) |  | | (1.001;1.002) | | (1.001;1.002) | (1.001;1.002) | (1.001;1.002) |
| Ghana | (1.004;1.02) | (1.004;1.02) | (1.004;1.02) | (1.004;1.02) |  | | (1.001;1.002) | | (1.001;1.002) | (1.001;1.002) | (1.001;1.002) |
| Guinea | (1.004;1.02) | (1.004;1.02) | (1.004;1.02) | (1.004;1.02) |  | | (1.001;1.002) | | (1.001;1.002) | (1.001;1.002) | (1.001;1.002) |
| Liberia | (1.004;1.02) | (1.004;1.02) | (1.004;1.02) | (1.004;1.02) |  | | (1.001;1.002) | | (1.001;1.002) | (1.001;1.002) | (1.001;1.002) |
| Mali | (1.004;1.02) | (1.004;1.02) | (1.004;1.02) | (1.004;1.02) |  | | (1.001;1.002) | | (1.001;1.002) | (1.001;1.002) | (1.001;1.002) |
| Niger | (1.004;1.02) | (1.004;1.02) | (1.004;1.02) | (1.004;1.02) |  | | (1.001;1.002) | | (1.001;1.002) | (1.001;1.002) | (1.001;1.002) |
| Nigeria | (1.004;1.02) | (1.004;1.02) | (1.004;1.02) | (1.004;1.02) |  | | (1.001;1.002) | | (1.001;1.002) | (1.001;1.002) | (1.001;1.002) |
| Senegal | (1.004;1.02) | (1.004;1.02) | (1.004;1.02) | (1.004;1.02) |  | | (1.001;1.002) | | (1.001;1.002) | (1.001;1.002) | (1.001;1.002) |
| Sierra Leone | (1.004;1.02) | (1.004;1.02) | (1.004;1.02) | (1.004;1.02) |  | | (1.001;1.002) | | (1.001;1.002) | (1.001;1.002) | (1.001;1.002) |
| Togo | (1.004;1.02) | (1.004;1.02) | (1.004;1.02) | (1.004;1.02) |  | | (1.001;1.002) | | (1.001;1.002) | (1.001;1.002) | (1.001;1.002) |

Note: CAF, Central African Republic; DRC, Democratic Republic of the Congo; Sao TP, Sao Tome and Principe
